# Unsteady MHD flow of tangent hyperbolic ternary hybrid nanofluid in a darcy-forchheimer porous medium over a permeable stretching sheet with variable thermal conductivity

**DOI:** 10.12688/f1000research.158629.2

**Published:** 2025-03-10

**Authors:** Asfaw Tsegaye Moltot, Eshetu Haile Gorfie, Gurju Awgichew Zergaw, Hunegnaw Dessie

**Affiliations:** 1 Department of Mathematics, Bahir Dar University, Bahir Dar, Amhara, Ethiopia

**Keywords:** Tangent hyperbolic fluid, Ternary Hybrid nanofluid, Viscous Dissipation, non-linear thermal radiation, variable thermal conductivity, permeable stretching sheet

## Abstract

**Background:**

This research investigates the unsteady magnetohydrodynamic (MHD) flow, heat, and mass transfer of tangent hyperbolic ternary hybrid nanofluids over a permeable stretching sheet. The study considers three types of nanoparticles—aluminum oxide (Al₂O₃), copper (Cu), and titanium oxide (TiO₂)—dispersed in a base fluid of ethylene glycol (C₂H₆O₂). This ternary hybrid nanofluid (Al₂O₃–Cu–TiO₂/C₂H₆O₂) has potential applications in cooling systems, biomedical uses for targeted drug delivery and hyperthermia treatments, heat exchangers, and polymer processing techniques like extrusion and casting.

**Methods:**

This study will examine the combined effects of Weissenberg number, power law index, nanoparticle volume fraction, viscous dissipation, magnetic field, heat generation, nonlinear thermal radiation, temperature ratio, Joule heating, Brownian motion, thermophoresis, porous permeability, variable thermal conductivity, Eckert number, Prandtl number, Schmidt number, chemical reaction, velocity ratio, and Forchheimer number on the electrical conductivity of unsteady flow in tangent hyperbolic ternary hybrid nanofluids. The governing equations are transformed into similarity equations using appropriate transformations and solved numerically with the MATLAB BVP5C package. The results are validated against data from published articles to ensure reproducibility.

**Results:**

The findings reveal that an increase in the Weissenberg and Forchheimer numbers reduces the velocity profile, while the temperature distribution increases. The variable thermal conductivity parameter (Γ) leads to a higher temperature profile, indicating improved heat transfer. Higher nanoparticle concentrations in the nanofluids and hybrid nanofluids result in enhanced skin friction, Nusselt number, and Sherwood number. Ternary hybrid nanofluids show the most significant improvement in heat transfer and thermal conductivity.

**Conclusions:**

Ternary hybrid nanofluids significantly enhance heat and mass transfer, showing potential for applications in cooling systems, drug delivery, and polymer processing. The numerical results are consistent with previous research, confirming the reliability and reproducibility of the findings.

**Nomenclature and notations**
*x, y*
: Space coordinates [m]
*u, v*
: Velocity components [m/s]
*B*_0_
: Uniform magnetic field [T]
*C _p_*
: Specific heat [J/kg·K]
*T _w_*
: Wall temperature [K]
*T*_∞_
: Temperature of the ambient fluid [K]
*C _w_*
: Concentration at the wall [mol/m ^3^]
*C*_∞_
: Concentration of the ambient fluid
*A*
: unsteady parameter
*C _f_*
: Skin friction
*Sh _x_*
: Local Sherwood number
*We*
: Weissenberg number
*u _w_*
: Velocity of Stretching Sheet along x-axis [m/s]
*κ* ^∗^
: Rosseland mean absorption coefficient [m ^−1^]
*κ*
: Thermal conductivity [W/m·K]
θw: Ratio temperature
Γ: Variable thermal conductivity parameter
θ: Dimensionless temperature
Φ: Dimensionless concentration function
υthnf: Kinematic viscosity of ternary hybrid nanofluid [m ^2^/s]
*K*
: Porous permeability parameter
*C _r_*
: Chemical reaction [s ^−1^]
*Sc*
: Schmidt number
*Ec*
: Eckert number
*D _B_*
: Brownian diffusion coefficient [m ^2^/s]
*D _T_*
: Thermophoresis diffusion coefficient [m ^2^/s]
*I*
: Identity vector
*γ*˙: Shear rate [s ^−1^]
*V*
: Velocity vector [m/s]
*E*
: Shear stress tensor [Pa]
*P*
: Pressure [Pa]
*M*
: Magnetic field parameter
*Nb*
: Brownian motion parameter
*Nt*
: Thermophoresis parameter
*Nu _x_*
: Nusselt number
*S*
: Suction and injection parameter
*Pr*
: Prandtl number
*Q*
: Uniform volumetric heat source/sink [W/m ^3^]
*Q*_0_
: Heat generation ( *Q*_0_ *>* 0)[W/m ^3^]
*Fr*
: Forchheimer number
*R*
: Non-linear thermal radiation parameter
*Re _x_*
: Local Reynolds number
f′: Dimensionless velocity
( *ρC _p_*) *_thnf_*
: Heat capacitance of ternary hybrid nanofluid [J/m ^3^·K]
*ρ _thnf_*
: Density of ternary hybrid nanofluid [kg/m ^3^]
*κ _thnf_*
: Thermal conductivity of ternary hybrid nanofluid [W/m·K]
∞: Ambient condition
*η*
: Similarity variable
*σ _thnf_*
: Electrical conductivity of ternary hybrid nanofluid [S/m]
*μ _f_*
: Dynamic viscosity of base fluid [Pa·s]
*σ* ^∗^
: Stefan-Boltzmann constant [W/m ^2^·K ^4^]
*μ _hnf_*
: Dynamic viscosity of hybrid nanofluid [Pa·s]
*κ _f_*
: Thermal conductivity of base fluid [W/m·K]
*ρ _f_*
: Density of base fluid [kg/m ^3^]
*τ*
: Ratio of heat capacity
( *ρC _p_*) *_p_*
: Heat capacity of a nanoparticle [J/m ^3^·K]
( *ρC _p_*) *_f_*
: Heat capacity of a base fluid [J/m ^3^·K]
*μ*_0_
: Limiting viscosity at zero shear rate [Pa·s]
*μ*_∞_
: Limiting viscosity at infinite [Pa·s]
*σ _f_*
: Electrical conductivity of base fluid [S/m]
ϕ1: Nanoparticle volume fraction of *Al*_2_ *O*_3_
ϕ2: Nanoparticle volume fraction of *Cu*
ϕ3: Nanoparticle volume fraction of *TiO*_2_
_ *f*
: Base fluid
_ *p*
: Nanoparticle
_ *hnf*
: Hybrid nanofluid
_ *thnf*
: Ternary hybrid nanofluid
_ *w*
: Wall condition
_∞: Ambient condition
’: First derivative with respect to *η*
”: Second derivative with respect to *η*
κ*, σ ^*^
: Denotes radiative or electrical properties

## Introduction

Non-Newtonian fluids play a crucial role in many applications due to their diverse behaviors. Non-Newtonian fluids have a viscosity that changes with the shear rate. Their flow behavior can be more complex, exhibiting various properties such as shear-thinning or shear-thickening. A non-Newtonian fluid is a specific type of fluid, like ketchup, toothpaste, and paints, where the relationship between shear and stress is nonlinear. Non-Newtonian fluids have a wide range of applications across various industries due to their unique flow characteristics, like, food industry, cosmetics and personal care, construction, textiles, etc. Tangent hyperbolic fluids are a type of non-Newtonian fluid characterized by their stress-strain relationship involving the hyperbolic tangent function. This model captures complex flow behaviors, making it suitable for describing materials that exhibit both viscous and elastic properties under varying shear rates. Tangent hyperbolic fluids represent a significant category of non-Newtonian fluids that exhibit complex flow behavior, essential for various industrial and scientific applications. Their unique properties enable better modeling and understanding of materials that behave differently than simple fluids, making them valuable in diverse fields such as food science, cosmetics, pharmaceuticals, and biomedical engineering. Hashim et al. ^1^ examined the heat and mass transfer behavior of non-Newtonian nanofluids in a non-parallel vertical enclosure. Rehman et al. ^2^ explored the flow behavior of non-Newtonian nanofluids between intersecting planes, including the effects of slip mechanisms at the boundaries. Nadeem et al. ^3^ examined the unsteady tangent hyperbolic hybrid nanofluid flow across an exponentially stretched sheet. They concluded that analyzing how fuzzy parameters affect the behavior of nanofluids could provide valuable insights into optimal conditions for specific applications. Alqahtani et al. ^4^ numerically examined how the combination of wedge angle and energy transfer during melting affects the flow of ternary hybrid magnetohydrodynamics (MHD) Nano liquid across a permeable wedge. This exploration reveals the intricate effects of wedge angle, heat sources/sinks, and Lorentz forces on the forced convective flow of tangent hyperbolic nanofluids over a permeable wedge. Amjad et al. ^5^ investigated the behavior of a tangent hyperbolic MHD nanofluid in a two-dimensional framework over an exponentially stretched sheet. According to their observations, the boundary layer is reduced by both the magnetic parameter and the Weissenberg number. Alkaoud et al. ^6^ investigated the characteristics of tangent hyperbolic fluid flow across a stretching sheet and discovered that fluid velocity decreases and temperature rises with increasing power-law index, slip velocity parameter, porosity parameter, and magnetic number. Choudhary et al. ^7^ explored the formation of boundary layers in tangent hyperbolic fluid flow through a diverging permeable channel, analyzing the effects of a porous medium, suction or blowing, and a heat source.

Nanofluids are advanced colloidal fluids that consist of a base fluid (such as water, oil, or ethylene glycol) combined with nanoparticles typically ranging in size from 1 to 100 nanometers. These nanoparticles can be made from various materials, including metals (like copper and silver), oxides (such as alumina and silica), and carbon-based materials (like graphene). Nanofluids represent a significant advancement in thermal management technology, offering enhanced heat transfer capabilities and efficiency across various applications. Ongoing research continues to explore their properties, optimize formulations, and expand their applicability in diverse fields. Hybrid nanofluids are advanced fluids that consist of two or more different types of nanoparticles suspended in a base fluid. This combination aims to optimize thermal and physical properties, enhancing heat transfer capabilities beyond what single-component nanofluids can achieve. The presence of multiple types of nanoparticles can lead to a significant increase in thermal conductivity, improving heat transfer efficiency. Hybrid nanofluids offer a promising approach to enhance heat transfer performance in various applications. By leveraging the unique properties of multiple nanoparticles, they provide improved thermal management solutions in diverse fields, from industrial processes to renewable energy systems. Ternary hybrid nanofluids are advanced fluids that incorporate three different types of nanoparticles to enhance thermal and transport properties. By combining multiple nanoparticles, these fluids can achieve superior heat transfer capabilities compared to conventional fluids or even binary hybrid nanofluids. The addition of different types of nanoparticles can optimize properties such as thermal conductivity, viscosity, and stability, making them suitable for a variety of applications, including cooling systems, heat exchangers, and energy storage. Boujelbene et al. ^8^ explored heat transfer and entropy generation in nanofluid flow within an inclined channel. Alqawasmi et al. ^9^ investigated the effects of Cattaneo-Christov heat exchange on the flow of ternary hybrid nanofluids over a spinning disc, excluding non-linear radiant heat. Their findings indicated that an increase in the magnitude of the suction and injection parameters leads to a reduction in the velocity distribution profile. Jat et al. ^10^ investigated the impact of various nanoparticle shapes on the radiating hybrid nanofluid flow, particularly over a nonlinear stretchable porous sheet. Mahboobtosi et al. ^11^ studied the impact of utilizing a ternary hybrid nanofluid in place of a single nanofluid. They concluded that improvements in the curvature parameter, volume fraction, and shape factor resulted in enhanced velocity and temperature profiles. Farooq ^12^ examined the mixed convection of stagnation point flow of ternary hybrid nanofluids toward a vertical Riga plate. He concluded that when the mixed convection parameter is introduced to counteract the flow, the temperature profile decreases while the velocity profile increases. Abbas et al. ^13^ examined the mixed convection of ternary hybrid nanofluids during cilia transport through a curved channel. Their findings indicated that increasing the cilia length parameter enhances the liquid velocity. Nabwey et al. ^14^ investigated the MHD of two-dimensional incompressible boundary layer flow of non-Newtonian Carreau ternary hybrid nanofluids with heat transfer over an exponentially stretched curved surface. They demonstrated that the heat transfer rate and skin friction increase as one moves from the base fluid to mono, hybrid, and then ternary nanofluids. Jamshed et al. ^15^ explored in order to find an efficient heat-transmitting fluid to replace standard fluids and revolutionary nanofluids. Noreen ^16^ utilized heat radiation to study the MHD flow of ternary hybrid nanofluids between double disks. The results indicated that increasing variations in the thermal relaxation parameter lead to a decline in the thermal distribution profile. Al Garalleh ^17^ investigated heat diffusion and mass transport in the flow of ternary hybrid nanofluids ( *TiO*_2_ − *Al*_2_ *O*_3_ − *SiO*_2_) over a stretching/shrinking wedge geometry. The study concluded that the role of ethylene glycol (EG) in heat transport is vital for ensuring the efficiency, safety, and reliability of thermal management systems, making it an essential component in modern engineering solutions for thermal regulation. Priyadharshini et al. ^18^ explored the optimal design and performance estimation of a ternary hybrid nanofluid using advanced machine learning prediction techniques. Their investigation revealed that the ternary hybrid nanofluid exhibits superior thermal performance compared to hybrid nanofluids. Patil et al. ^19^ investigated the flow of tangent hyperbolic ternary hybrid nanofluids over a rough-yawed cylinder, induced by impulsive motion in a mixed convection mechanism with periodic magnetohydrodynamics.

Flow that varies with time is termed unsteady flow, whereas flow that remains constant over time is referred to as steady flow. Engineers and researchers typically prefer steady flow due to its more manageable and predictable nature, allowing for better control in various applications. However, the influence of unsteadiness on flow behavior is significant and cannot be overlooked. Consequently, researchers must account for the effects of unsteady flow in their analyses and designs to ensure accurate modeling and performance optimization. Unsteady flow is a critical concept in fluid dynamics, impacting various engineering fields. Its study allows for the accurate modeling of dynamic systems, ensuring safety, efficiency, and performance in real-world applications. Understanding unsteady flow phenomena is essential for designing and optimizing systems that experience time-dependent changes. Many practical applications experience unsteady flow, making it crucial for accurate modeling and analysis. Ignoring unsteadiness can lead to incomplete or inaccurate results. Unsteady flow captures important dynamic behaviors such as turbulence, flow separation, and transient heat transfer, which are essential in various engineering disciplines.

Kebede et al. ^20^ investigated the time-dependent flow of Williamson nanofluid and found that both the velocity and temperature gradients decrease with increasing unsteadiness parameters within the boundary layer. Jamal et al. ^21^ investigated the unsteady flow of MHD incompressible tangent hyperbolic fluid with nanofluid particles along a stretching surface. Their results indicated that the velocity field decreases as the Weissenberg number and power-law index increase, while the thermal and concentration fields continue to rise under the same conditions. Reddy et al. ^22^ focused on the effects of Lorentz force, Joule heating, and viscous dissipation on the unsteady flow of tangent hyperbolic liquid past a vertical plate. Their findings revealed that an increase in the Weissenberg number and magnetic field weakens the velocity profile. Kumar et al. ^23^ studied unsteady MHD oscillatory flow over a vertically permeable stretching plate in a viscous, incompressible fluid. Their findings indicated that fluid velocity increases over time. Choudhary et al. ^8^ analyzed the unsteady laminar flow, heat, and mass transfer of a hybrid nanofluid over a non-linearly stretchable porous sheet, considering the effects of thermal radiation and gyrotactic microorganisms.

MHD is the study of the behavior of electrically conducting fluids in the presence of a magnetic field. It combines principles from both fluid dynamics and electromagnetism to analyze the motion of plasmas, liquid metals, and other conductive fluids. MHD plays a crucial role in understanding and predicting the behavior of conductive fluids in many natural and industrial processes. MHD has important applications in various fields, including astrophysics (e.g., solar flares and stellar formation), metallurgy (e.g., casting processes), and engineering (e.g., cooling systems for nuclear reactors), biomedical applications (e.g., magneto-fluid dynamics). Krishna and Chamkha ^24^ studied the MHD squeezing flow of a water-based nanofluid through a saturated porous medium situated between two parallel disks, while considering the effects of Hall current. In the squeezing flow of a nanofluid, the Lorentz force, which governs the impact of magnetic fields on fluid motion, is predominantly influenced by the external magnetic field. In many practical applications, the magnetic field strength is typically quite weak. When the applied magnetic field is low, the induced magnetic field can become negligible in comparison to the external magnetic field. As a result, the impact of the induced magnetic field can be ignored without substantially influencing the flow behavior. The Hall Effect, which results from the movement of charged particles in a magnetic field, may be minimal if the fluids conductivity is low or if the fluid’s velocity is relatively slow. In water-based nanofluids, conductivity can vary, meaning the Hall Effect might not be significant enough to be considered in the analysis of the squeezing flow. The investigation provides further information on the magnetohydrodynamics tangent hyperbolic nanofluid flow as provided in these references. ^25–^ ^32^

A porous medium, or porous material, is a substance that contains pores (voids) capable of being filled with fluids (liquids or gases). These materials are distinguished by their ability to facilitate fluid flow through their structure, influenced by factors such as pore size, shape, distribution, and connectivity. The study of porous media is essential across various fields due to their role in fluid dynamics, resource management, and environmental sustainability. In many practical applications involving porous media, particularly at low flow rates, flow behavior is primarily governed by Darcy’s law, which assumes that flow resistance is proportional to the flow rate. The Forchheimer equation, on the other hand, incorporates nonlinear inertial effects that become significant only at high velocities or in highly permeable media. Chamkha ^33^ examined non-Darcy fully developed mixed convection flow in a channel embedded in a porous medium (referred to as a porous medium channel), considering the effects of heat generation/absorption and hydromagnetic influences.

The Darcy-Forchheimer equation is a model that describes fluid flow through porous media by combining Darcy’s law, which addresses laminar flow, with Forchheimer extension, which accounts for inertial effects occurring in higher velocity flows. The Darcy-Forchheimer equation serves as a crucial tool for understanding and predicting the behavior of fluids in porous media, particularly under varying flow conditions. The Darcy-Forchheimer equation has various applications, including modeling fluid flow in reservoir rocks for oil and gas extraction to enhance extraction techniques. In hydrology, it is used to analyze groundwater movement and contaminant transport. In chemical engineering, it aids in designing reactors and filtration systems that involve flow through porous materials. Jawad et al. ^34^ studied the effects of heat and mass transfer on the convective Darcy-Forchheimer flow of a Maxwell nanofluid over a linearly stretched porous sheet. Their findings indicated that increased thermal radiation and thermophoresis enhance the temperature distribution. Saeed et al. ^35^ investigated heat transfer and the effects of electromagnetic forces on the MHD flow of couple-stress hybrid nanofluids over a Darcy-Forchheimer model in a symmetric flow scenario with variable viscosity. Alessa et al. ^36^ studied the Darcy-Forchheimer flow of water-based *Al* − *Al*_2_ *O*_3_ */Cu* − *Al*_2_ *O*_3_ hybrid nanofluids past a heated stretchable plate, incorporating heat consumption/generation and non-linear radiation effects. Their results indicated that the velocity of the hybrid nanofluid decreased as the magnetic field parameter increased.

Viscous dissipation is the process where uneven forces in adjacent fluid layers convert work into heat. Viscous dissipation in tangent hyperbolic fluid flow is a crucial factor that affects thermal behavior, flow dynamics, and overall system efficiency. Understanding its implications helps in optimizing processes involving non-Newtonian fluids, ensuring better performance in various industrial applications. Using convective boundary conditions and viscous dissipation, Hussain et al. ^37^ concentrated on the thermo-physical characteristics of MHD tangent hyperbolic fluid flow across a non-linear stretched sheet. Nandi and Kumbhakar ^38^ studied the MHD boundary layer flow of a TH nanofluid past a stretched wedge with velocity slip boundary conditions.

Numerous research teams have explored unsteady hybrid nanofluid flow over stretching sheets using various geometries and approaches. The literature includes diverse techniques employed to analyze the heat and mass fluxes associated with this type of flow. Of these researchers, Jamrus et al. ^39^ performed a numerical investigation to examine the flow characteristics and heat transfer of unsteady flow over a permeable. Stretching sheet, utilizing a ternary hybrid nanofluid ( *Al*_2_ *O*_3_ − *Cu* − *TiO*_2_ */H*_2_ *O*) under the influence of suction and a magnetic field but the important concepts like viscous dissipation, porous medium, Darcy- Forchheimer flow, non-linear thermal radiation, variable thermal conductance, non-Newtonian like tangent hyperbolic fluid, thermophoresis diffusion, Brownian diffusion, joule heating, chemical reaction, and heat generation/absorption are not considered. Jamal et al. ^21^ investigated the unsteady flow of an MHD tangent hyperbolic fluid influenced by the presence of nanoparticles over a stretching sheet. On their study, they have not included viscous dissipation, Darcy-Forchheimer flow, variable thermal conductance, joule heating, non-linear thermal radiation, chemical reaction, and heat generation/absorption.

Thus, to the authors’ knowledge, the combined effects of viscous dissipation, Darcy-Forchheimer flow, variable thermal conductance, non-Newtonian fluid, nonlinear thermal radiation, Joule heating, chemical reactions, heat generation/absorption, Brownian motion, and thermophoresis on the electrical conductivity of unsteady flow in tangent hyperbolic ternary hybrid nanofluids over a stretching sheet have not been addressed in the existing literature. Therefore, this study will examine the combined effects of viscous dissipation, Darcy-Forchheimer flow, variable thermal conductance, nonlinear thermal radiation, Joule heating, chemical reactions, heat generation/absorption, Brownian motion, and thermophoresis on the electrical conductivity of unsteady flow in tangent hyperbolic ternary hybrid nanofluids. These nanofluids consist of *Al*_2_ *O*_3_, *Cu*, and *TiO*_2_ nanoparticles suspended in ethylene glycol, flowing over a stretching sheet. This study is significant as it combines theoretical analysis and practical application, providing valuable insights into the behavior of tangent hyperbolic ternary hybrid nanofluids under complex flow conditions. Such nanofluids have a wide range of applications in industries including cooling systems, heat exchangers, energy systems, biomedical applications, etc. The outcomes will not only advance scientific knowledge but also have meaningful implications in various engineering fields. The governing partial differential equations are transformed into ordinary differential equations using suitable variable transformations. We employed the BVP5C algorithm in MATLAB to solve these transformed equations. The effects of various phenomena on temperature, concentration, velocity profiles, rates of heat and mass transfer, and skin friction coefficients are illustrated through graphical representations and tabular data.

## Methods

### Mathematical formulation of the flow problem

**Figure 1.**
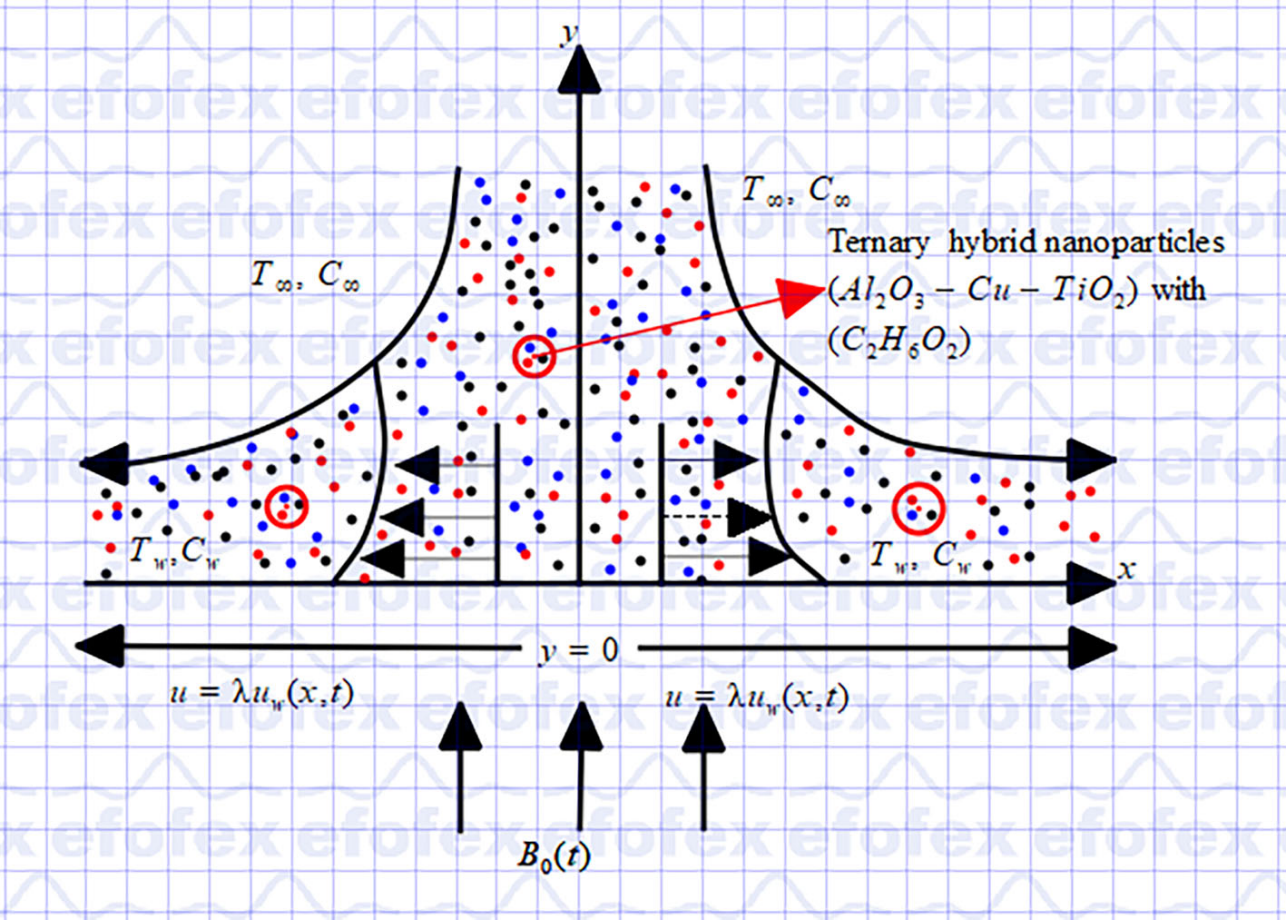
Flow configuration and coordinate system.

We examine the ternary hybrid nanofluid is laminar boundary layer, two dimensional, time-dependent, incompressible flow of an electrically conducting Tangent hyperbolic fluid over a permeable stretching sheet, surface with velocity $U_{w}\left(x,t\right)=\mathrm{ax}/\left(1-\mathrm{ct}\right)$ , where *a* is a positive constant, t is time, and c is the time-dependent parameter of this problem. The wall mass suction velocity is assumed to be $,V_{w}\left(x,t\right)$ . The *x*-axis is oriented along the direction of the stretching sheet, whereas the *y*-axis is perpendicular to it. The velocity components are represented as u and v , corresponding to the *x*- and *y*-axes, respectively. The well temperature, $T_{w}$ , ambient temperature $,T_{\infty }$ , well concentration, $C_{w}$ , and ambient concentration, $C_{\infty }$ are considered constants. Figure 1 graphically illustrate the physical flow model in this study, using Cartesian coordinate. The model for momentum, mass, and heat transfer incorporate thermophoresis, Brownian motion, viscous dissipation, chemical reactions, variable thermal conductivity, non-linear thermal radiation, heat generation, magnetic dissipation, and wall mass suction. When a constant magnetic field is applied perpendicular to the surface in the positive y-direction, the induced magnetic field is considered negligible and ignored. Assuming that the porosity of the porous material remains constant, we also take Darcy-Forchheimer flow into account in our investigation. The energy calculations are made simpler by treating all fluid parameters as constants and assuming that the system enters thermal equilibrium. The energy equation takes into consideration the effects of viscous dissipation, in which viscosity causes kinetic energy to be transformed into thermal energy. Through the dispersion of *Al*_2_ *O*_3_, *Cu*, and *TiO*_2_ nanoparticles in *C*_2_ *H*_6_ *O*_2_ medium, the ternary hybrid nanofluid is created. According to Refs. 40, the heat conductivity of the ternary hybrid nanomaterial are proportional and linearly connected to the distributed temperature.

$$\kappa_{\text{thnf}}\left(T\right)=\left[1+\Gamma \frac{T-T_{\infty }}{T_{w}-T_{\infty }}\right]\kappa_{\text{thnf}}=\kappa_{\text{thnf}}\left(1+\Gamma \theta \right)$$ (1)

where Γ is variable thermal conductivity.

**Table 1.** Thermophysical properties of utilized nanoparticles and base fluid (EG). ^3, 18, 39^

Physical Properties	( *C*_2_ *H*_6_ *O*_2_)( *f* )	( *Al*_2_ *O*_3_( $\phi_{1}$ ))	( *Cu*( $\phi_{2}$ ))	*TiO*_2_( $\phi_{3}$ )
*ρ*	1115	3970	8933	4250
*C _p_*	2430	765	385	686.2
*κ*	0.253	40	400	8.9538
*σ*	0.107	3.5×10 ^7^	5.96×10 ^7^	1.0×10 ^−12^

### Constitutive equation of tangential hyperbolic

The tangential hyperbolic model is used to describe the flow behavior of shear-thinning fluids. In this model, the relationship between the shear stress *tau* and shear rate $\dot{\gamma }$ is given by a hyperbolic tangent function. The following formulas provide the basic equation for the tangential hyperbolic fluid ^4, 7, 26, 27^:

$$\tau =-\mathrm{PI}+E,E=\left[\mu_{\infty }+\left(\mu_{0}+\mu_{\infty }\right)\tanh{\left(N\dot{\gamma }\right)}^{n}\right]\dot{\gamma }$$ (2)

The shear rate *γ*˙ is assumed

$$\dot{\gamma }={\left(\frac{1}{2}\sum_{i}\;\;\sum_{j}\;\;{\dot{\gamma }}_{\mathrm{ij}}{\dot{\gamma }}_{\mathrm{ji}}\right)}^{\frac{1}{2}}={\left(\frac{1}{2}\pi \right)}^{\frac{1}{2}}$$ (3)

where N is material constant, π is the second invariant strain rate tensor and is specified by $\pi =\frac{1}{2}\mathrm{tr}[\nabla V+$ ${{\left(\nabla V\right)}^{t}]}^{2}$ , and t is transpose. We assuming $\mu_{\infty }=0$ , the shear stress tensor E for $\left(N\dot{\gamma }\right)<1$ gives to

$$E=\mu_{0}\left[{\left(N\dot{\gamma }\right)}^{n}\right]\dot{\gamma }=\mu_{0}\left[{\left(1+N\dot{\gamma }-1\right)}^{n}\right]\dot{\gamma }=\mu_{0}\left[1+n\left(N\dot{\gamma }-1\right)\right]\dot{\gamma }$$ (4)

The governing equations for the flow problem are: ^7, 21, 39^

$$\frac{\partial u}{\partial x}+\frac{\partial v}{\partial y}=0$$ (5)

$$\frac{\partial u}{\partial t}+u\frac{\partial u}{\partial x}+v\frac{\partial u}{\partial y}=\frac{\mu_{\text{thnf}}}{\rho_{\text{thnf}}}\left[\left(1-n\right)+n\Gamma \sqrt{2}\frac{\partial u}{\partial y}\right]\frac{\partial^{2}u}{\partial y^{2}}-\frac{\sigma_{t\mathrm{hnf}}B^{2}}{\rho_{t\mathrm{hnf}}}u-\frac{\mu_{t\mathrm{hnf}}}{\rho_{t\mathrm{hnf}}K_{p}}u-\frac{1}{\rho_{\text{thnf}}}\frac{W_{c}}{\sqrt{W}}u^{2}$$ (6)

$$\begin{array}{c} \frac{\partial T}{\partial t}+u\frac{\partial T}{\partial x}+v\frac{\partial T}{\partial y}=\frac{1}{{\left(\rho C_{p}\right)}_{\text{thnf}}}\frac{\partial }{\partial y}\left[\kappa_{\text{thnf}}\left(T\right)\frac{\partial T}{\partial y}\right]+\frac{1}{{\left(\rho C_{p}\right)}_{\text{thnf}}}\frac{16\sigma^{*}}{3\kappa^{*}}\frac{\partial }{\partial y}\left(T^{3}\frac{\partial T}{\partial y}\right)+\frac{Q_{0}\left(T-T_{\infty }\right)}{{\left(\mathrm{\rho Cp}\right)}_{\text{thnf}}}\\ +\frac{\mu_{\text{thnf}}}{{\left(\rho C_{p}\right)}_{\text{thnf}}}\frac{n\Gamma }{\sqrt{2}}\frac{\partial u}{\partial y}{\left(\frac{\partial u}{\partial y}\right)}^{2}+\frac{\mu_{\text{thnf}}}{{\left(\rho C_{p}\right)}_{\text{thnf}}}\left(1-n\right){\left(\frac{\partial u}{\partial y}\right)}^{2}+\frac{\mu_{\text{thnf}}}{{\left(\rho C_{p}\right)}_{\text{thnf}}}\frac{1}{K_{p}}u^{2}+\frac{\sigma_{\text{thnf}}B_{0}^{2}}{{\left(\mathrm{\rho Cp}\right)}_{\text{thnf}}}u^{2}\\ +\frac{1}{{\left(\rho C_{p}\right)}_{\text{thnf}}}\frac{W_{c}}{\sqrt{W}}u^{3}+\frac{{\left(\rho C_{p}\right)}_{\mathrm{np}}}{{\left(\rho C_{p}\right)}_{\text{thnf}}}\left[D_{B}\frac{\partial C}{\partial z}\frac{\partial T}{\partial z}+\frac{D_{T}}{T_{\infty }}{\left(\frac{\partial T}{\partial z}\right)}^{2}\right] \end{array}$$ (7)

$$\frac{\partial C}{\partial t}+u\frac{\partial C}{\partial x}+v\frac{\partial C}{\partial y}=D_{B}\frac{\partial^{2}C}{\partial y^{2}}+\frac{D_{T}}{T_{\infty }}\left(\frac{\partial^{2}T}{\partial y^{2}}\right)-C_{0}\left(C-C_{\infty }\right)$$ (8)

Subject to boundary condition given by ^39^

$$\left\{ \begin{array}{l} v=V_{w}\left(x,t\right),u=\lambda u_{w}\left(x,t\right),T=T_{w},C=C_{w},\mathrm{at}\;y\to 0\\ u\to 0,T\to T_{\infty },C\to C_{\infty },\mathrm{as}\;y\to \infty \end{array}\right.$$ (9)

where (u,v) are the velocity components in the axes(x,y),T and C are the temperature and concentration of the THNs, respectively, σ is the electrical conductivity, and a and c represent positive constants, $\mu_{\text{thnf}},\rho_{\text{thnf}},{\left(\rho C_{p}\right)}_{\text{thnf}},\sigma_{t\mathrm{hnf}}$ , are the effective ternary hybrid nanofluids (THNs) viscosity, density and THNs heat capacitance, electrical conductivity of THNs, respectively.

The ternary hybrid nanofluids thermophysical characteristics are described as ^14, 18, 39^:

$$\begin{array}{c} \frac{\rho_{\text{thnf}}}{\rho_{f}}=\left(1-\phi_{1}\right)\left(1-\phi_{2}\right)\left(1-\phi_{3}\right)+\phi_{3}\frac{\rho_{s3}}{\rho_{f}}+\phi_{2}\frac{\rho_{s2}}{\rho_{f}}+\phi_{1}\frac{\rho_{s1}}{\rho_{f}}\\ \frac{\mu_{\text{thnf}}}{\mu_{f}}=\frac{1}{{\left(1-\phi_{1}\right)}^{2.5}{\left(1-\phi_{2}\right)}^{2.5}{\left(1-\phi_{3}\right)}^{2.5}}\\ \frac{{\left(\rho C_{p}\right)}_{\text{thnf}}}{{\left(\rho C_{p}\right)}_{f}}=\left(1-\left(\phi_{1}+\phi_{2}+\phi_{3}\right)\right)+\phi_{1}\frac{{\left(\rho C_{p}\right)}_{s1}}{{\left(\rho C_{p}\right)}_{f}}+\phi_{2}\frac{{\left(\rho C_{p}\right)}_{s2}}{{\left(\rho C_{p}\right)}_{f}}+\phi_{3}\frac{{\left(\rho C_{p}\right)}_{s3}}{{\left(\rho C_{p}\right)}_{f}} \end{array}$$

$$\begin{array}{c} \frac{\kappa_{\mathrm{nf}}}{\kappa_{f}}=\frac{\kappa_{s3}+2\kappa_{f}-2\phi_{3}(\kappa_{f}-\kappa_{s3})}{\kappa_{s3}+2\kappa_{f}+\phi_{3}(\kappa_{f}-\kappa_{s3})};\frac{\kappa_{\mathrm{hnf}}}{\kappa_{\mathrm{nf}}}=\frac{\kappa_{s2}+2\kappa_{nf}-2\phi_{2}(\kappa_{nf}-\kappa_{s2})}{\kappa_{s2}+2\kappa_{nf}+\phi_{2}(\kappa_{nf}-\kappa_{s2})}\\ \frac{\kappa_{\mathrm{thnf}}}{\kappa_{\mathrm{hnf}}}=\frac{\kappa_{s1}+2\kappa_{hnf}-2\phi_{1}(\kappa_{hnf}-\kappa_{s1})}{\kappa_{s1}+2\kappa_{hnf}+\phi_{1}(\kappa_{hnf}-\kappa_{s1})}\\ \frac{\sigma_{\mathrm{nf}}}{\sigma_{f}}=\frac{\sigma_{s3}+2\sigma_{f}-2\phi_{3}(\sigma_{f}-\sigma_{s3})}{\sigma_{s3}+2\sigma_{f}+\phi_{3}(\sigma_{f}-\sigma_{s3})};\frac{\sigma_{\mathrm{hnf}}}{\sigma_{\mathrm{nf}}}=\frac{\sigma_{s2}+2\sigma_{nf}-2\phi_{2}(\sigma_{nf}-\sigma_{s2})}{\sigma_{s2}+2\sigma_{nf}+\phi_{2}(\sigma_{nf}-\sigma_{s2})}\\ \frac{\sigma_{\mathrm{thnf}}}{\sigma_{\mathrm{hnf}}}=\frac{\sigma_{s1}+2\sigma_{hnf}-2\phi_{1}(\sigma_{hnf}-\sigma_{s1})}{\sigma_{s1}+2\sigma_{hnf}+\phi_{1}(\sigma_{hnf}-\sigma_{s1})} \end{array}$$

Here, the subscripts f,nf,hnf , and thnf stand for traditional fluid, NF, HNF, and THNs, respectively, and the letters 1,2, and 3 stand for the three selected nanoparticles.

Since the governing equations are in partial differential form, they are reduced to a system of ordinary differential equations through the use of similarity transformations. Further, to obtain similarity solutions of Eq. (5) to Eq. (8), the unsteady magnetic field *B*_0_ is in the form *B*_0_= *B*/(1− *ct*) ^1/2^ where *B* is a constant. The unsteady magnetic field *B*_0_= *B*/(1− *ct*) ^1/2^ can induce an electric field according to Maxwell’s equations. Specifically, Faraday’s law of electromagnetic induction states that a time-dependent magnetic field generates a circulating electric field. Since *B*_0_ varies with time due to the presence of (1− *ct*) ^1/2^ its time derivative is nonzero, leading to an induced electric field. ^2^ This induced electric field can influence charge transport, modify current distributions, and potentially alter the overall nanofluid dynamics, making it an important factor in magnetohydrodynamic (MHD) applications.

The similarity transformations are given by ^21, 39^

$$\left\{ \begin{array}{l} \eta =y{\left(\frac{a}{\nu_{f}\left(1-\mathrm{ct}\right)}\right)}^{\frac{1}{2}},u=\frac{\mathrm{ax}}{1-\mathrm{ct}}f^{\prime }\left(\eta \right),v=-{\left(\frac{a\nu_{f}}{1-\mathrm{ct}}\right)}^{\frac{1}{2}}f\left(\eta \right)\\ V_{w}=-\sqrt{\frac{a\nu_{f}}{1-\mathrm{ct}}}S,\theta =\frac{T-T_{\infty }}{T_{w}-T_{\infty }},\Phi =\frac{C-C_{\infty }}{C_{w}-C_{\infty }} \end{array}\right.$$ (10)

where S is the constant wall mass transfer parameter. S=0 and S<0 denote the impermeable sheet and injection case, respectively. But in this study, only suction case is considered where S>0 . Equations (6) to (8) are simplified accordingly:

$$\begin{array}{c} \frac{A_{1}}{A_{2}}\left[\left(1-n\right)+\mathrm{nWe}f^{\prime \prime }\right]f^{\prime \prime \prime }-\left(A+\frac{A_{3}}{A_{2}}M+\frac{A_{1}}{A_{2}}K\right)f^{\prime }+\left(f-\frac{\mathrm{A\eta}}{2}\right)f^{\prime \prime }-\left(1+\frac{1}{A_{2}}\mathrm{Fr}\right){\left(f^{\prime }\right)}^{2}=0 \end{array}$$ (11)

$$\begin{array}{c} \frac{A_{4}}{A_{5}\mathrm{Pr}}\left(1+\Gamma \theta \right)\theta^{\prime \prime }+\frac{R}{A_{5}\mathrm{Pr}}\left[{\left(1+\left(\theta_{w}-1\right)\right)}^{2}\times 3\left(\theta_{w}-1\right)\theta^{\prime 2}+{\left(1+\left(\theta_{w}-1\right)\theta \right)}^{3}\theta^{\prime \prime }\right]\\ +(f-\frac{\mathrm{A\eta}}{2})\theta^{\prime }+\frac{1}{A_{5}}\mathrm{Q\theta}+\frac{1}{A_{5}}\left[\mathrm{Nb}\theta^{\prime }\Phi^{\prime }+\mathrm{Nt}\theta^{\prime 2}\right]+\mathrm{Ec}\left[\frac{A_{1}}{A_{5}}K+\frac{A_{3}}{A_{5}}M\right]{\left(f^{\prime }\right)}^{2}\\ +\frac{A_{1}}{A_{5}}\frac{n}{2}\text{WeEc}{\left(f^{\prime \prime }\right)}^{3}+\frac{A_{1}}{A_{5}}\left(1-n\right)\mathrm{Ec}{\left(f^{\prime \prime }\right)}^{2}+\frac{1}{A_{5}}\text{FrEc}{\left(f^{\prime }\right)}^{3}=0 \end{array}$$ (12)

$$\Phi^{\prime \prime }+\frac{\mathrm{Nt}}{\mathrm{Nb}}\theta^{\prime \prime }+\mathrm{Scf}\Phi^{\prime }-\mathrm{Sc}\frac{\mathrm{A\eta}}{2}\Phi^{\prime }-\mathrm{Sc}C_{r}\Phi =0.$$ (13)

with subject to boundary conditions

$$\left\{ \begin{array}{l} f\left(0\right)=S,f^{\prime }\left(0\right)=\lambda ,\theta \left(0\right)=1,\Phi \left(0\right)=1,\mathrm{at}\;\eta =0\\ f^{\prime }\left(\eta \right)\to 0,\theta \left(\eta \right)\to 0,\Phi \left(\eta \right)\to 0,\mathrm{as}\;\eta \to \infty \end{array}\right.$$ (14)

where, the Weissenberg number ( *We*), magnetic field parameter ( *M*), porosity parameter ( *K*), Forchheimer number ( *Fr*), Prandtl number ( *Pr*), non-linear thermal radiation ( *R*), ratio of temperature $\left(\theta_{w}\right),Q\;$ is the heat generation ( *Q >* 0) or absorption parameter ( *Q <* 0), Brownian motion parameter ( *Nb*), Thermophoresis parameter ( *Nt*), Eckert number ( *Ec*), Schmidt number ( *Sc*), Chemical reaction ( *C _r_*), unsteady parameter( *A*), and *A*_1_ *, A*_2_ *, A*_3_ *, A*_4_, and *A*_5_ are dynamic viscosity, density, electrical conductivity, thermal conductivity, and heat capacitance of the ternary hybrid nanofluid, respectively can be described as follows:

$$\begin{array}{c} \text{We}=\frac{x\Gamma }{1-\mathrm{ct}}\sqrt{\frac{2a^{3}}{\nu_{f}\left(1-\mathrm{ct}\right)}},M=\frac{\sigma_{f}B^{2}}{\rho_{f}a},K=\frac{\nu_{f}}{aK_{p}},\mathrm{Fr}=\frac{W_{c}}{\sqrt{W}}\frac{x}{\left(1-\mathrm{ct}\right)\rho_{f}},\mathrm{Pr}=\frac{\nu_{f}{\left(\mathrm{\rho Cp}\right)}_{f}}{\kappa_{f}},\\ R=\frac{16\sigma^{*}T_{\infty }^{3}}{3\kappa \kappa^{*}},\theta_{w}=\frac{T_{w}}{T_{\infty }},Q=\frac{Q_{0}\left(1-\mathrm{ct}\right)}{a{\left(\mathrm{\rho Cp}\right)}_{f}},\mathrm{Nb}=\frac{D_{B}\tau \left(C_{w}-C_{\infty }\right)}{\nu_{f}},\mathrm{Nt}=\frac{D_{T}\tau \left(T_{w}-T_{\infty }\right)}{\nu_{f}T_{\infty }},\\ \mathrm{Ec}=\frac{u_{w}^{2}}{\left(T_{w}-T_{\infty }\right){\left(\mathrm{Cp}\right)}_{f}\left(1-\mathrm{ct}\right)},\mathrm{Sc}=\frac{\nu_{f}}{D_{B}},C_{r}=\frac{C_{0}\left(1-\mathrm{ct}\right)}{a},A=\frac{c}{a},\\ A_{1}=\frac{\mu_{\text{thnf}}}{\mu_{f}},A_{2}=\frac{\rho_{\text{thnf}}}{\rho_{f}},A_{3}=\frac{\sigma_{\text{thnf}}}{\sigma_{f}},A_{4}=\frac{\kappa_{t}\mathrm{hnf}}{\kappa_{f}},A_{5}=\frac{{\left(\mathrm{\rho Cp}\right)}_{\text{thnf}}}{{\left(\mathrm{\rho Cp}\right)}_{f}} \end{array}$$

In this context, the parameter λ, representing the velocity ratio, assumes various values: λ=0 corresponds to the static sheet, λ<0 signifies the shrinking sheet, and λ>0 denotes the stretching sheet. The Skin friction coefficient $\left(C_{f}\right)$ , local Nusselt number $\left({\mathrm{Nu}}_{x}\right)$ , and local Sherwood number $\left({\mathrm{Sh}}_{x}\right)$ are given by ^21^:

$$C_{\mathrm{fx}}=\frac{\tau_{w}}{\rho_{f}u_{w}^{2}},Nu_{x}=\frac{xq_{w}}{\kappa_{f}\left(T_{w}-T_{\infty }\right)},Sh_{x}=\frac{xj_{m}}{D_{B}\left(C_{w}-C_{\infty }\right)},$$ (15)

where $\tau_{w},q_{w}$ , and $j_{m}$ represents the wall shear stress, the wall heat flux, and mass flux from the stretching sheets, which is given by:

$$\left\{ \begin{array}{l} \tau_{\mathrm{xz}}=\mu_{\text{thnf}}{\left[\left(1-n\right)\frac{\partial u}{\partial y}+\frac{n\Gamma }{\sqrt{2}}{\left(\frac{\partial u}{\partial y}\right)}^{2}\right]}_{y=0}\\ q_{w}=-\left[\kappa_{\text{thnf}}+\frac{16\sigma^{*}T_{\infty }^{3}}{3\kappa^{*}}\right]{\left(\frac{\partial T}{\partial y}\right)}_{y=0},j_{m}=-D_{B}{\left(\frac{\partial C}{\partial y}\right)}_{y=0}. \end{array}\right.$$ (16)

The dimensionless skin friction coefficient, Nusselt number, and the Sherwood number are given as follows:

$$\left\{ \begin{array}{l} {\left(Re_{x}\right)}^{\frac{1}{2}}C_{\mathrm{fx}}=A_{1}\left[\left(1-n\right)f^{\prime \prime }\left(0\right)+\frac{\mathrm{nWe}}{2}{\left(f^{\prime \prime }\left(0\right)\right)}^{2}\right]\\ {\left(Re_{x}\right)}^{-\frac{1}{2}}Nu_{x}=-\left(A_{4}+R\right)\theta^{\prime }\left(0\right),{\left(Re_{x}\right)}^{-\frac{1}{2}}Sh_{x}=-\Phi^{\prime }\left(0\right) \end{array}\right.$$ (17)

where $Re_{x}=\frac{xu_{w}}{\nu_{f}}$ is the local Reynolds number.

### Numerical method

Equations (11) to (13) and with the boundary condition (14) are numerically solved by using the MAT LAB software ( https://github.com/asfawmat/Tan_22BVP) utilizing the bvp5c algorithm. ^39^ BVP5C is a numerical algorithm in MATLAB designed to solve boundary value problems (BVPs) for ordinary differential equations. It extends the capabilities of previous algorithms, allowing for the efficient handling of complex systems that require multiple boundary conditions. BVP5C is a powerful tool for solving boundary value problems in various scientific and engineering applications. Its versatility, efficiency, and user-friendly nature make it an essential component in numerical analysis. BVP5C offers high accuracy for stiff boundary value problems, adaptive mesh refinement, and robustness in handling nonlinearities. Compared to finite difference methods, it achieves faster convergence with minimal grid dependence. BVP5C is widely used in structural analysis, heat transfer problems, and fluid dynamics to model systems governed by differential equations with boundary conditions. The algorithm is applicable in fields such as quantum mechanics and thermodynamics, where BVPs arise naturally in modeling physical phenomena. In order to apply the BVP5C with the shooting technique, the boundary value problems must be reduced to a system of first-order initial value problems (IVPs), as explained below. We use the following relations to convert the nonlinear higher-order boundary value problems into a system of first-order initial value problems.

Now let us defined the new variable by the equation

$$\left\{ \begin{array}{l} y_{1}=f,y_{2}=f^{\prime },y_{3}=f^{\prime \prime },y_{4}=\theta \\ y_{5}=\theta^{\prime },y_{6}=\Phi ,y_{7}=\Phi^{\prime } \end{array}\right.\;$$ (18)

Using Eq.(18), the three coupled higher order differential equations Eq.(11) to Eq. (13) can be written as

$$\left\{ \begin{array}{c} y_{1}^{\prime }=y_{2};\\ y_{2}^{\prime }=y_{3};\\ y_{3}^{\prime }=\frac{\left(A+\left(A_{3}/A_{2}\right)M+\left(A_{1}/A_{2}\right)K\right)y_{2}+\left(1+\left(\mathrm{Fr}/A_{2}\right)\right){\left(y_{2}\right)}^{2}-\left(y_{1}-\left(\mathrm{A\eta}\right)/2\right)y_{3}}{\left(1-n\right)+\mathrm{nWe}y_{3}}\\ y_{4}^{\prime }=y_{5};\\ \begin{array}{c} y_{5}^{\prime }=(-[\left(R/A_{5}\mathrm{Pr}\right)\left(3{\left(1+\left(\theta_{w}-1\right)y_{4}\right)}^{2}\left(\theta_{w}-1\right){\left(y_{5}\right)}^{2}\right)+\left(f-\left(\mathrm{A\eta}\right)/2\right)y_{5}+\left(1/A_{5}\right)Qy_{4}+\left(A_{1}\text{nWeEc}\right)/\left(2A_{5}\right){\left(y_{3}\right)}^{3}+\left(A_{1}/A_{5}\right)\left(1-n\right)\mathrm{Ec}{\left(y_{3}\right)}^{2}+\mathrm{Ec}(\left(A_{1}/A_{5}\right)K+(A_{3}/A_{5})M){\left(y_{2}\right)}^{2}+\left(A_{1}/A_{5}\right)\text{FrEc}{\left(y_{2}\right)}^{3}+\left(1/A_{5}\right)\left(\mathrm{Nb}y_{7}y_{5}+\mathrm{Nt}{\left(y_{5}\right)}^{2}\right)])/\left(\left(\frac{A_{4}}{A_{5}\mathrm{Pr}}\right)\left(1+\Gamma y_{4}\right)+\left(\frac{R}{A_{5}\mathrm{Pr}}\right){\left(1+\left(\theta_{w}-1\right)\theta \right)}^{3}\right):\\ y_{6}^{\prime }=y_{7};\\ y_{7}^{\prime }=\left(\text{AηSc}/2\right)y_{7}+\mathrm{Sc}C_{r}y_{6}-\mathrm{Sc}y_{1}y_{7}-\frac{\mathrm{Nt}}{\mathrm{Nb}}y_{5}^{\prime }; \end{array} \end{array}\right.$$ (19)

with corresponding initial conditions

$$\{y_{1}\left(0\right)=S,y_{2}\left(0\right)=\lambda ,y_{3}\left(0\right)=\beta_{1},y_{4}\left(0\right)=1,y_{5}\left(0\right)=\beta_{2},y_{6}\left(0\right)=1,y_{7}\left(0\right)=\beta_{3}$$ (20)

where $\beta_{1},\beta_{2}$ , and $\beta_{3}$ are the missing initial conditions and prime denote the differentiation with respect to η .

The system is solved in MATLAB using the BVP5C function, with a step size of 0.1 and a mesh size of 101. The shooting method employed provides greater precision, achieving a tolerance of $10^{-7}$ compared to earlier numerical methods. A comparison of our results with those from previous research is presented in Table 2, and the findings indicate a generally excellent agreement with earlier studies.

**Table 2.** A comparison between the numerical results of Gorla and Sidawi, ^41^ Waini et al., ^42^ Priyadlarshtni et al., ^18^ and Jamrus et al. ^39^ and the findings of the current investigation by evaluating the values of *θ*′(0) for different values of Pr under the conditions of *Nb* = 1 × 10 ^− 25^, when *Γ* = *M* = *K* = *A* = *n* = *We* = *Fr* = *R* = *θ*_*w*_ = *Q* = *Nt* = *Ec* = *Sc* = *C*_*r*_ = *S* = *H* = *ϕ*_1_ = *ϕ*_2_ = *ϕ*_3_ = 0.

Pr	Gorla and Sidawi ^41^	Waini et al. ^42^	Priyadlarshtni et al. ^18^	Jamrus et al. ^39^	Present study
2	0.9114	0.911353	0.9113	0.911358	0.911352771
6.13	-	1.759682	-	1.759685	1.759681702
7	1.8954	1.8954	1.8954	1.895403	1.8954
20	3.3539	3.353902	3.3539	3.353904	3.353901836

## Results and Discussion

In this section, we discuss the numerical solution for the time-dependent MHD hyperbolic tangent ternary hybrid nanofluid flowing over stretched surface. For temperature, velocity, concentration, local skin friction, Nusselt number, and Sherwood number under different physical parameter effects such as Weissenberg number (We) , magnetic field parameter (M) , spherical-shaped ${\mathrm{Al}}_{2}O_{3},\mathrm{Cu}$ , and ${\mathrm{TiO}}_{2}$ nanoparticle volume fraction $\left(\phi_{1},\phi_{2},\phi_{3}\right)$ , heat generation (Q) , Forchheimer number (Fr) , Prandtl number (Pr) , nonlinear thermal radiation(R) , unsteady parameter (A) , temperature ratio parameter $\left(\theta_{w}\right)$ , Eckert number (Ec) , Schmidt number (Sc) , chemical reaction $\left(C_{r}\right)$ , Porosity parameter (K) , Brownian (Nb) and thermophoresis (Nt) diffusion parameter are displays via graphical and table illustrations. Using Table 1, to present the results in the form of figures and tables, the following fixed numerical values for the parameters are used in this study: Pr= $7,\mathrm{Sc}=2,\mathrm{Nb}=0.5,\mathrm{Nt}=0.1,\lambda =1,\Gamma =0.3,\text{We}=0.5,Q=0.01,\mathrm{Fr}=0.3,K=0.1,S=0.1,n=0.1,M=0.2,R=1.5,\theta_{w}=1.2,A=0.1,\mathrm{Ec}=0.2,C_{r}=0.1$ , and $\phi_{1}=\phi_{2}=\phi_{3}=0.05$ , unless explicitly stated otherwise in the corresponding graphs. The selection of parametric values in this study is guided by a combination of physical relevance, material properties, flow regime characteristics, heat transfer conditions, and insights from prior empirical research. By aligning these values with specific physical scenarios, the model’s accuracy and applicability are enhanced, enabling more reliable predictions and deeper understanding of the system’s behavior.

### Velocity characteristics

**Figure 2.**
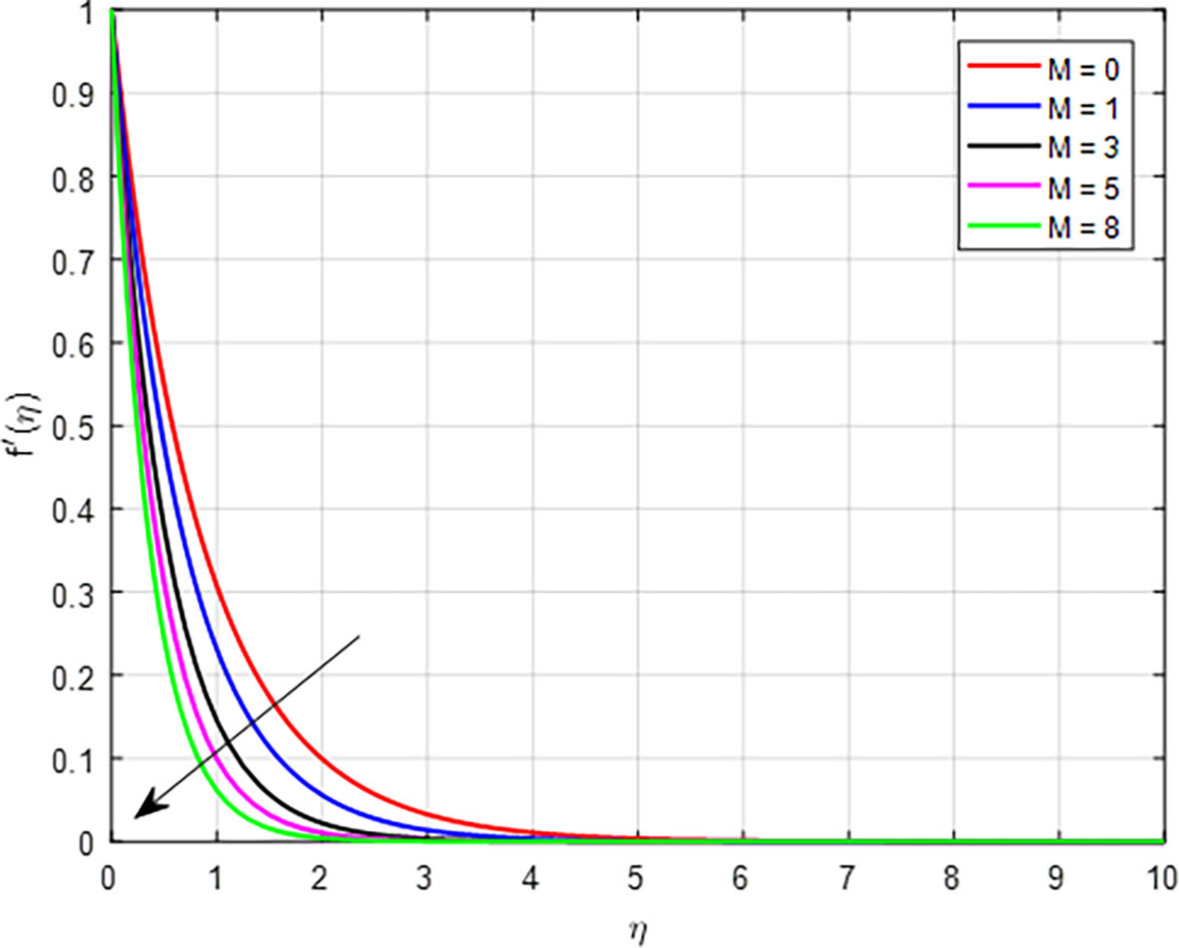
Velocity with *M*.

**Figure 3.**
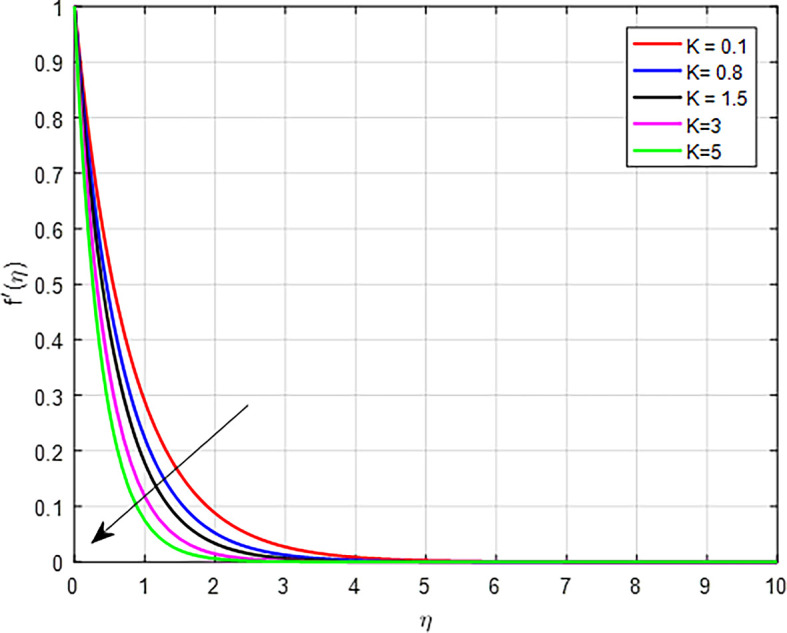
Velocity with *K*.

**Figure 4.**
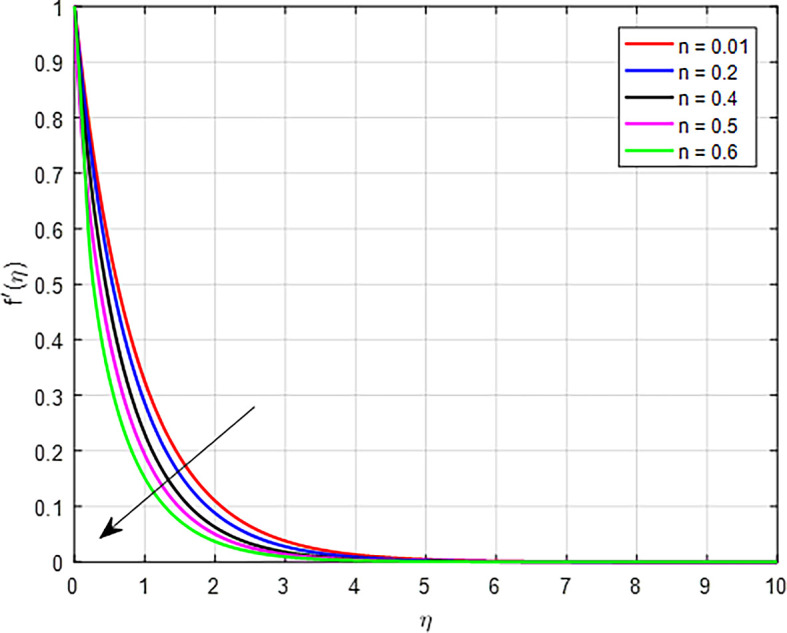
Velocity with *n*.

**Figure 5.**
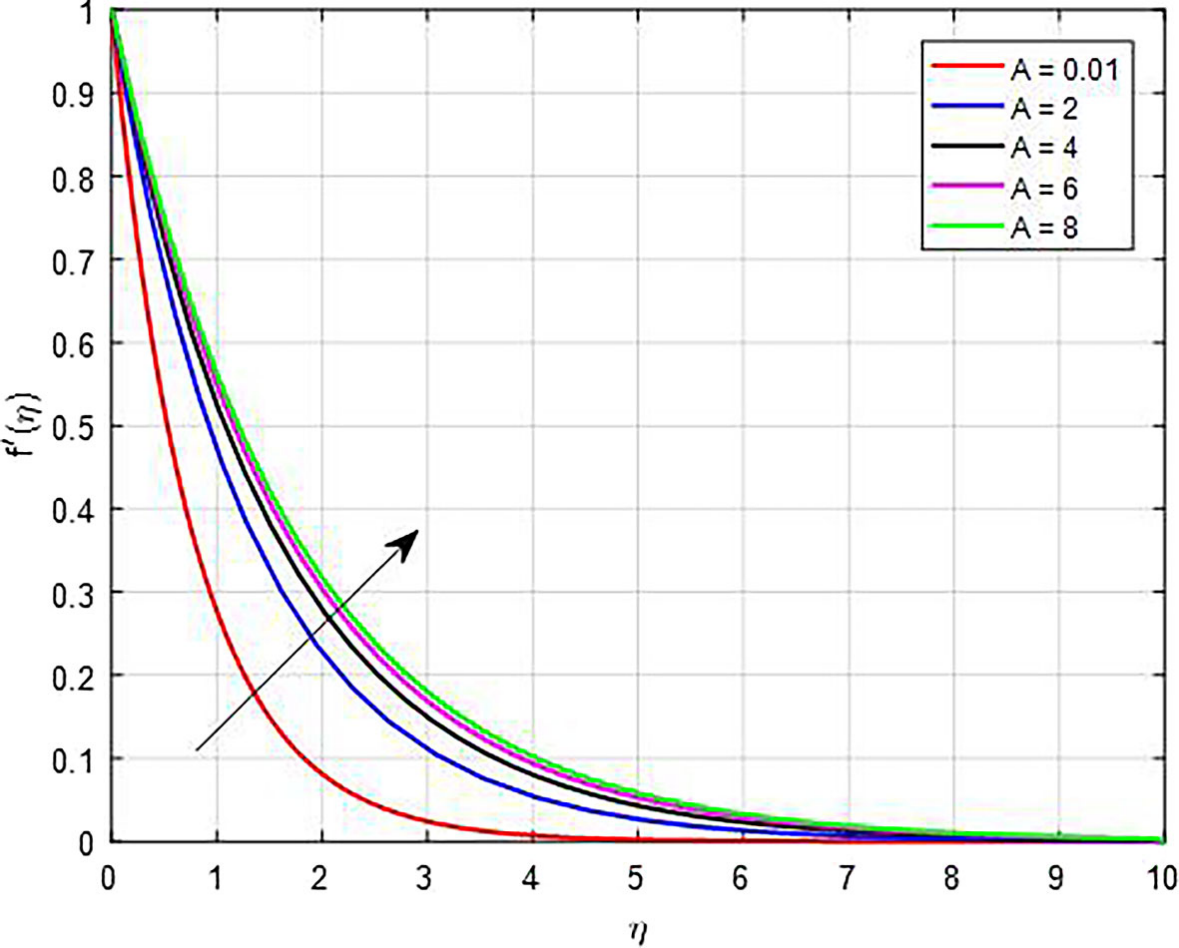
Velocity with *A*.

**Figure 6.**
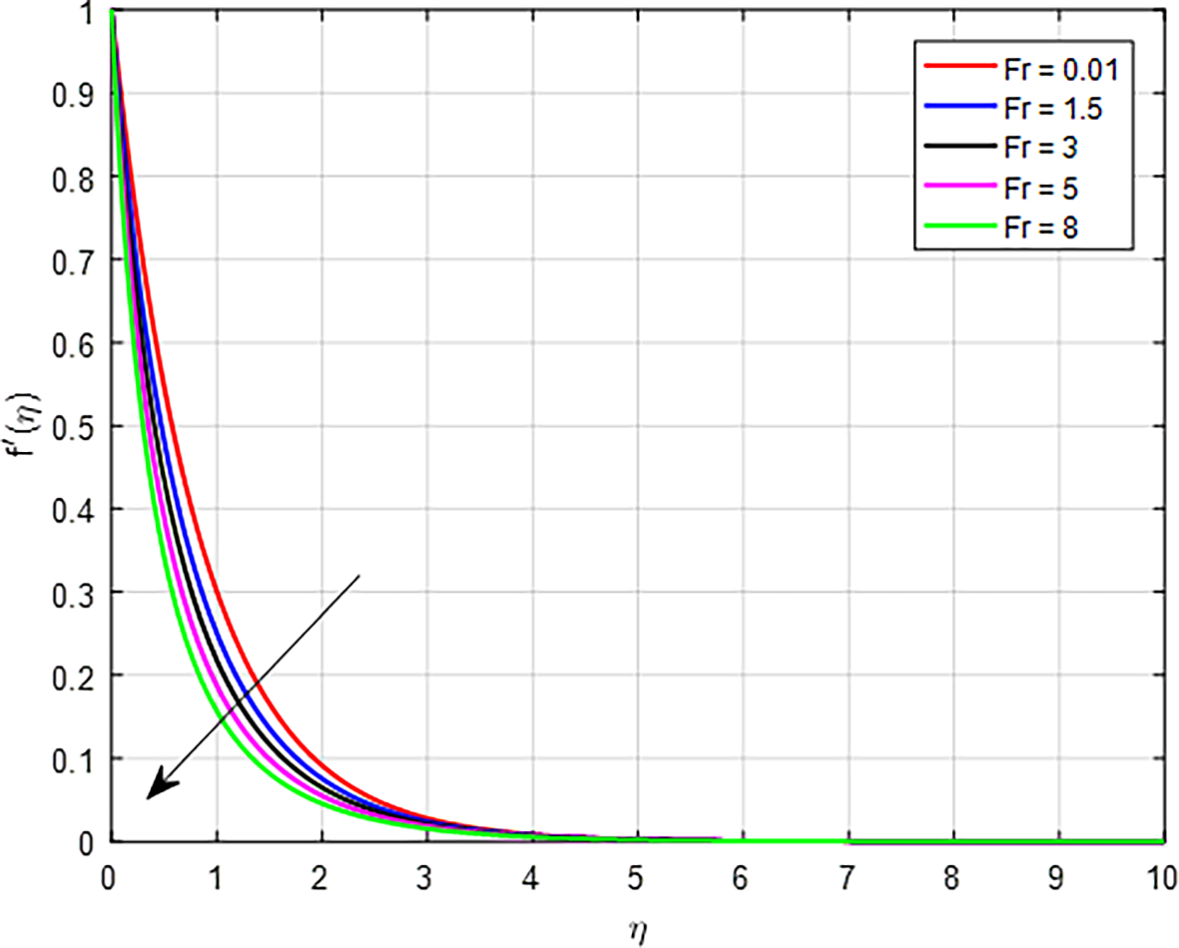
Velocity with *Fr*.

**Figure 7.**
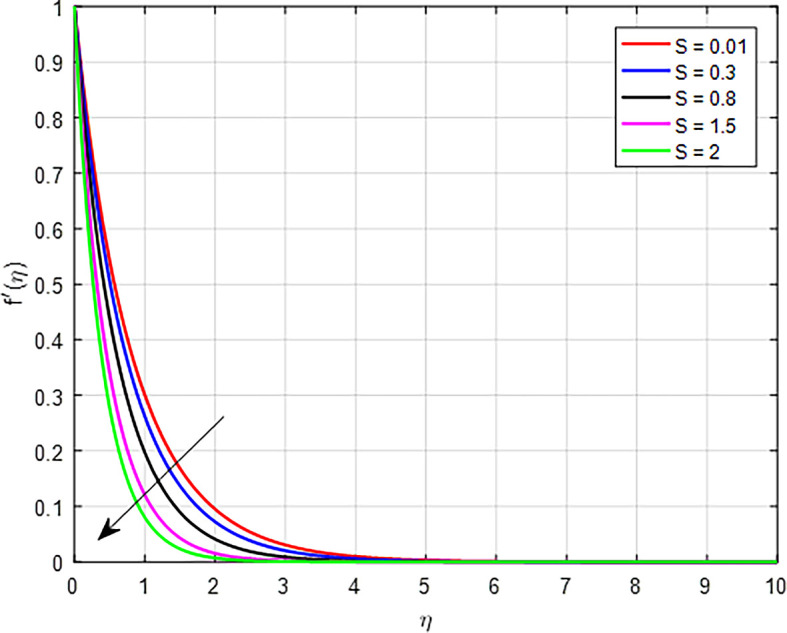
Velocity with *S*.

**Figure 8.**
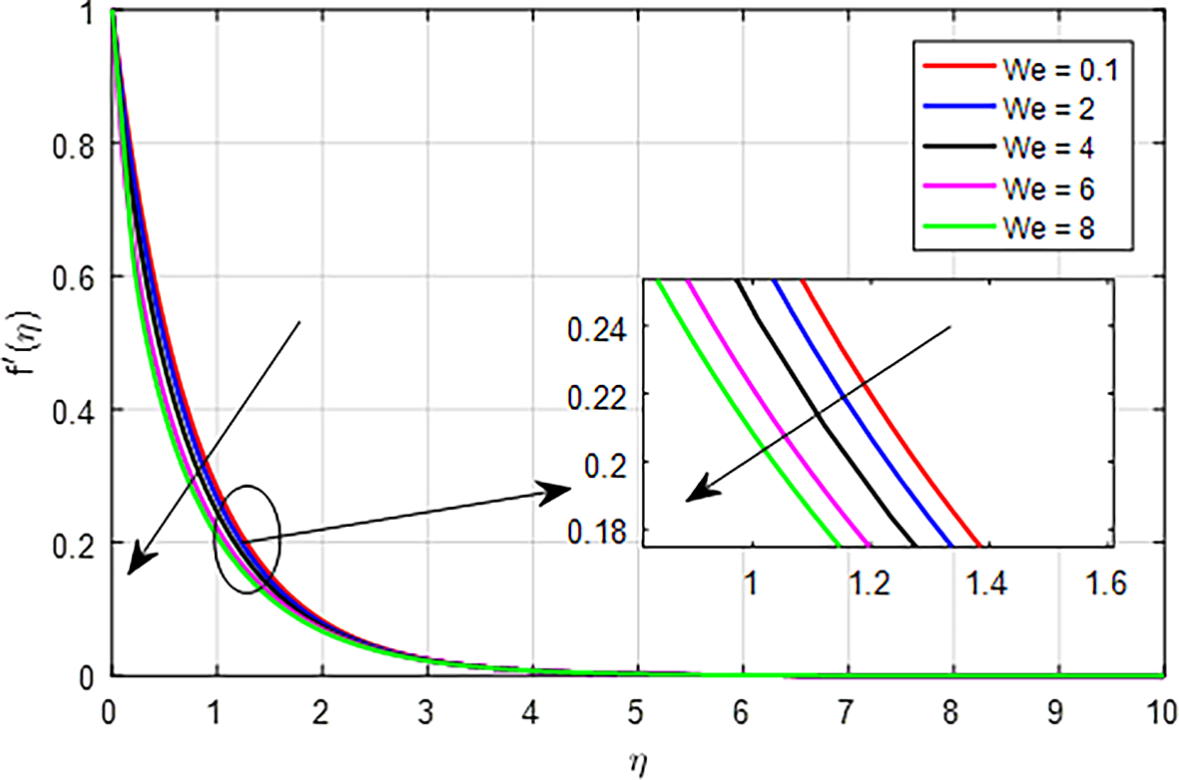
Velocity with *We*.

**Figure 9.**
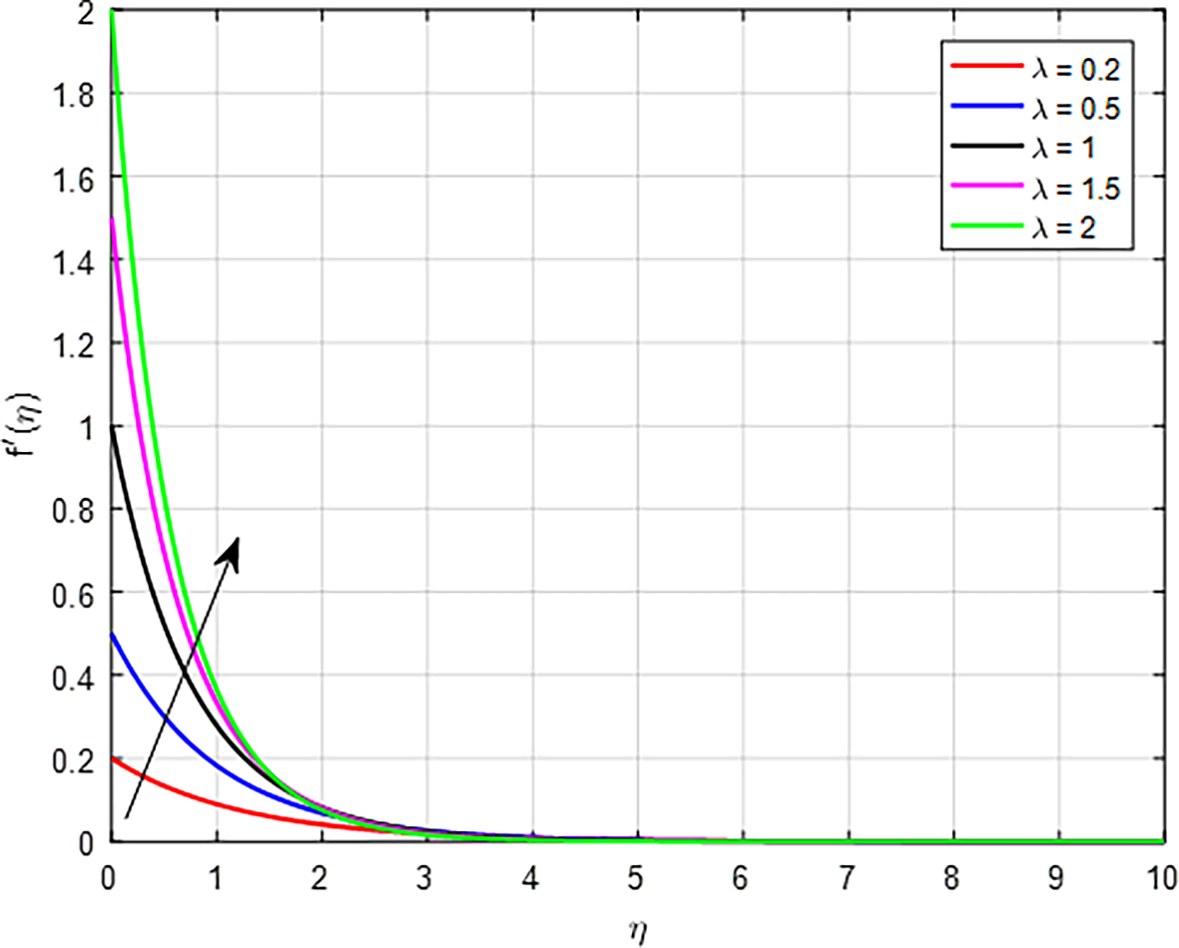
Velocity with *λ*.

**Figure 10.**
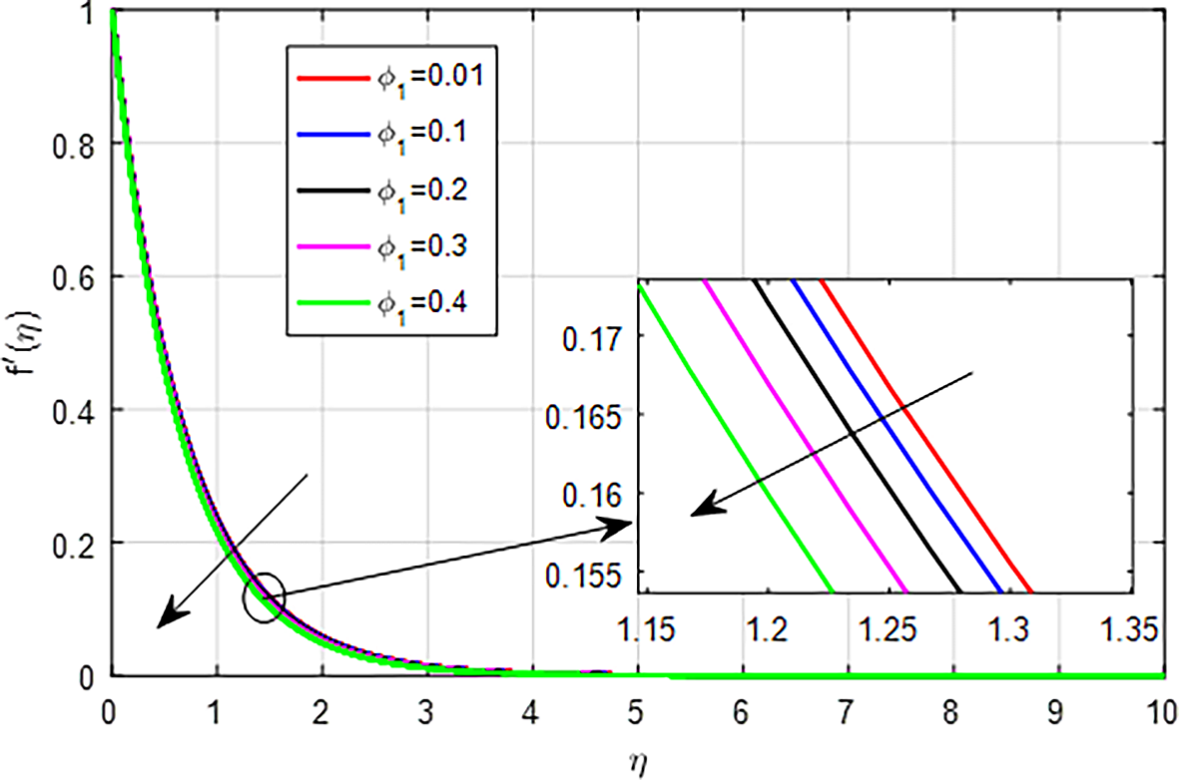
Velocity with *ϕ*_1_.

**Figure 11.**
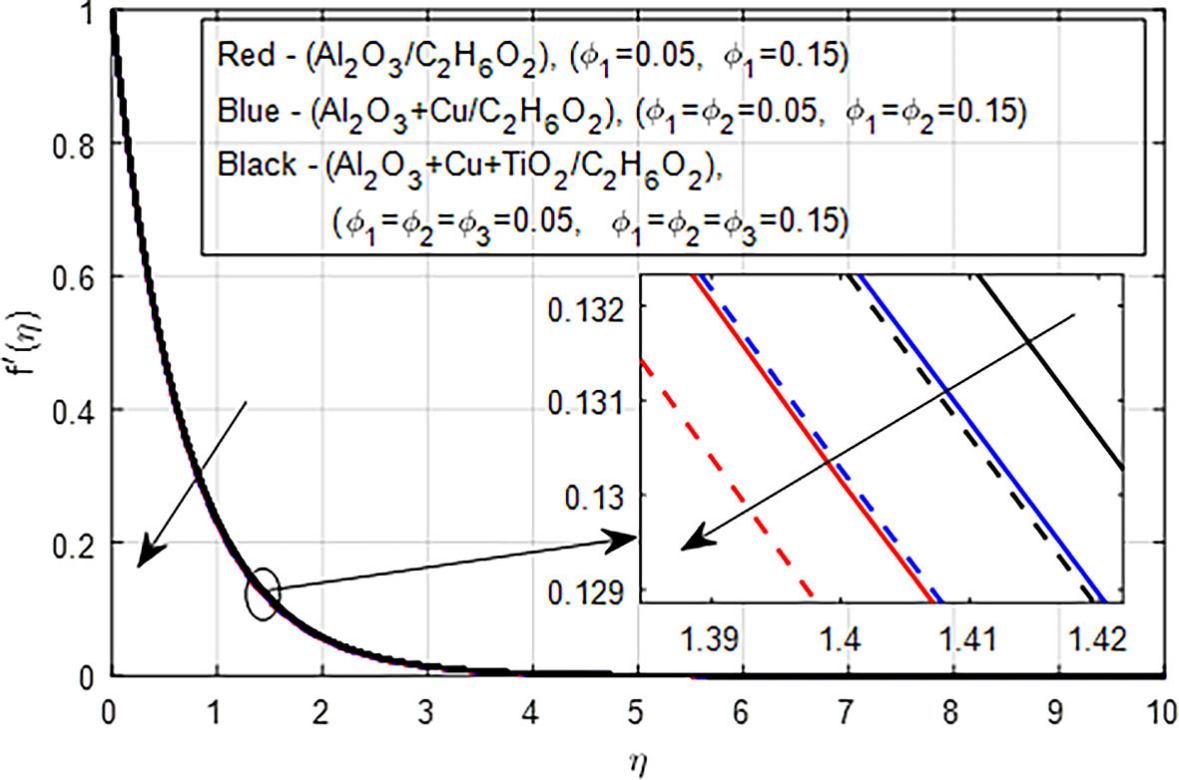
Velocity with *ϕ*_1_, *ϕ*_2_, *ϕ*_3_.

Figures 2- 10 depict the influence of several factors, like: magnetic field parameter (M) , porous permeability parameter (K) , Weissenberg number (We) , unsteady parameter (A) , power law index (n) , Forchheimer number (Fr) , suction parameter (S) , velocity ratio parameter (λ) , and nanoparticle volume fraction on scaled velocity $\left(f^{\prime }\left(\eta \right)\right)$ . Figure 2 shows how the scaled velocity profile is affected by the magnetic field parameter (M) . Physically, as the magnetic field effect increases, the interplay between the Lorentz force and the fluid’s motion leads to a decline in the velocity distribution, primarily due to increased resistance, damping effects, and changes in the dynamics of the flow. Figure 3 depict the influence of the porous permeability parameter (K) , on the velocity profile. When the value of the porous permeability parameter increases, the velocity distribution in a fluid flow can declines. Physically, in porous media, as the permeability increases, the inertial effects may become more pronounced, particularly in non-Darcy flow regimes. This can lead to a situation where the increased inertia counteracts the driving forces, resulting in a decline in the overall velocity. Higher permeability typically allows for easier flow through the porous medium. However, if the permeability increases too much, it can lead to a decrease in the pressure gradient needed to drive the flow. This results in reduced fluid velocity as the flow becomes less confined and more dispersed within the pores. Figure 4 illustrate the impact of power law index (n) on the velocity profile. As the power law index (n) , increases in a tangent hyperbolic fluid, the resulting increase in viscosity and flow resistance, combined with the fluid’s non-Newtonian characteristics, leads to a decline in the velocity distribution. Figure 5 display the effect of unsteady parameter on $\left(f^{\prime }\left(\eta \right)\right)$ . As the unsteady parameter increases, the balance between inertial and viscous forces shifts, leading to higher velocities due to enhanced acceleration, reduced damping, potential turbulence, and increased momentum transfer. The impact of Forchheimer parameter ( *Fr*) on the velocity profile illustrated in Figure 6. As the Forchheimer parameter increases, the combination of heightened inertial resistance, nonlinear flow behavior, increased energy losses, and complex flow distributions contributes to a diminished velocity profile in porous media. This highlights the interplay between viscous and inertial effects in determining flow behavior. The effect of suction parameter on the velocity distribution demonstrated in Figure 7. When suction is applied to the stretching sheet, the fluid will be pulled closer to the sheet, resulting in the thinning of the boundary layer. As the suction parameter increases, the combined effects of increased resistance to flow, enhanced viscous effects, flow redistribution, boundary layer dynamics, and reduced kinetic energy contribute to a diminished velocity profile. These factors illustrate how suction influences the overall flow behavior and velocity distribution in the system. The Weissenberg number ( *We*) is a dimensionless parameter that characterizes the relative importance of elastic (viscoelastic) effects compared to viscous effects in a fluid flow, particularly for non-Newtonian fluids such as tangent hyperbolic fluids. As the Weissenberg number increases, it indicates that the elastic effects become more significant, as depicted in Figure 8. As the Weissenberg number increases in a tangent hyperbolic fluid, the combined effects of increased elastic resistance, nonlinear flow behavior, thicker boundary layers, increased energy dissipation, and flow stabilization contribute to a diminished velocity profile. These factors highlight the complex interplay between elasticity and viscosity in determining the flow characteristics of non-Newtonian fluids. Figure 9 show the effect of the velocity ratio parameter ( *λ*) on the velocity distribution. When the velocity ratio ( *λ*) is high, the interactions between fluid layers (or between phases in a multiphase flow) become more dynamic. This enhances the momentum exchange, leading to higher velocities being transmitted through the flow field, thus increasing the overall velocity profile. As in Figure 10, as nanoparticle volume fraction ( *ϕ*_1_) increases, the interplay of increased viscosity, enhanced drag, altered fluid structure, and boundary effects collectively results in a decrease in the velocity profile of the fluid. Figure 11 depict the comparison of nanofluid ( *Al*_2_ *O*_3_ */C*_2_ *H*_6_ *O*_2_), hybrid nanofluid ( *Al*_2_ *O*_3_ − *Cu/C*_2_ *H*_6_ *O*_2_), and ternary hybrid nanofluid ( *Al*_2_ *O*_3_ − *Cu* − *TiO*_2_ */C*_2_ *H*_6_ *O*_2_) on the velocity distribution. When the concentration of nanoparticles in nanofluids, hybrid nanofluids, and ternary hybrid nanofluids increases, the velocity pro- file typically decreases due to increased viscosity, enhanced drag forces, complex microstructural changes, potential shear-thickening effects, and shifts in flow regime. These factors collectively hinder the smooth flow of the fluid, resulting in lower velocities. Ternary hybrid nanofluids often achieve the best velocity distribution among the three categories. The synergistic effects of multiple nanoparticles can enhance dispersion stability and thermal performance while managing viscosity better. This results in improved flow characteristics and potentially higher velocities compared to the other two types.

### Thermal characteristics

**Figure 12.**
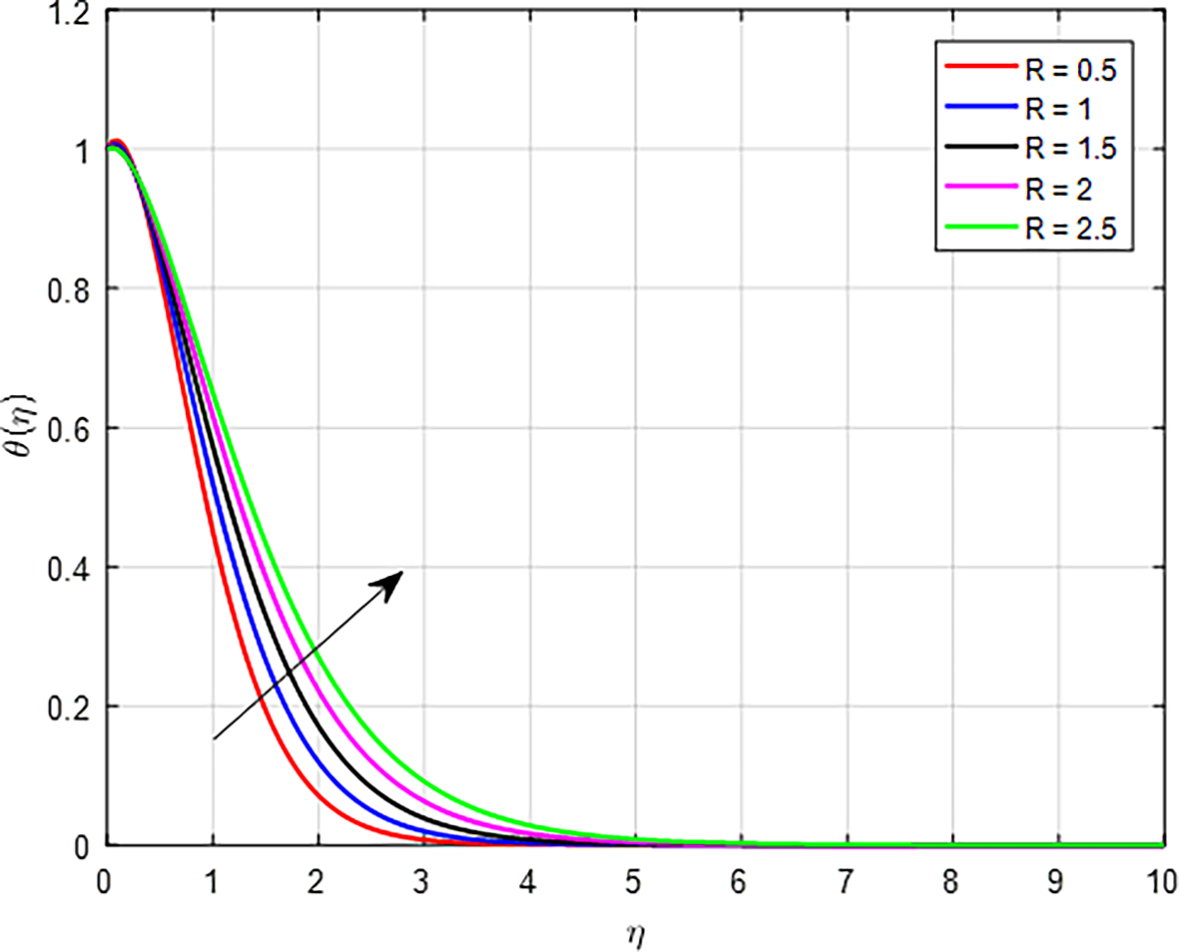
Temperature with *R*.

**Figure 13.**
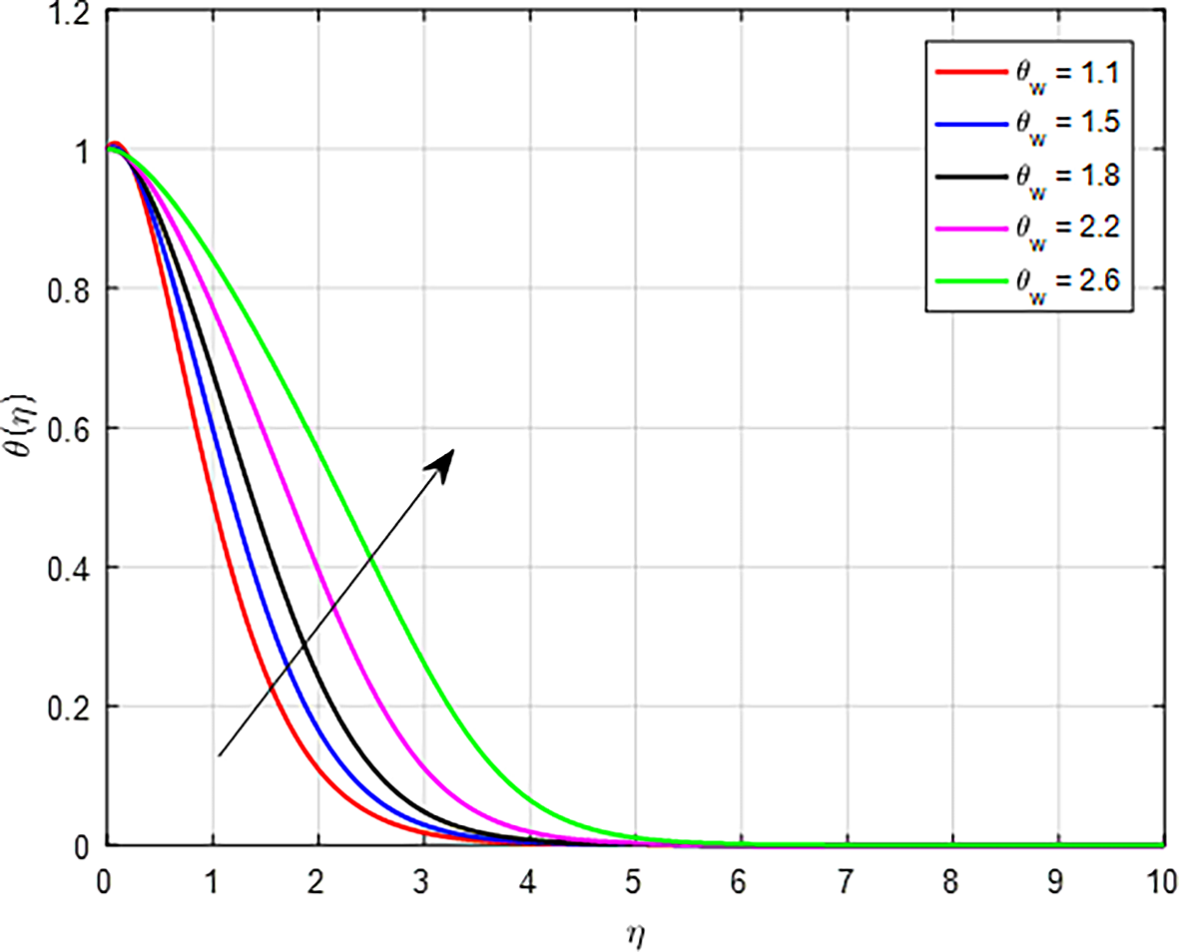
Temperature with *θ*_*w*_.

**Figure 14.**
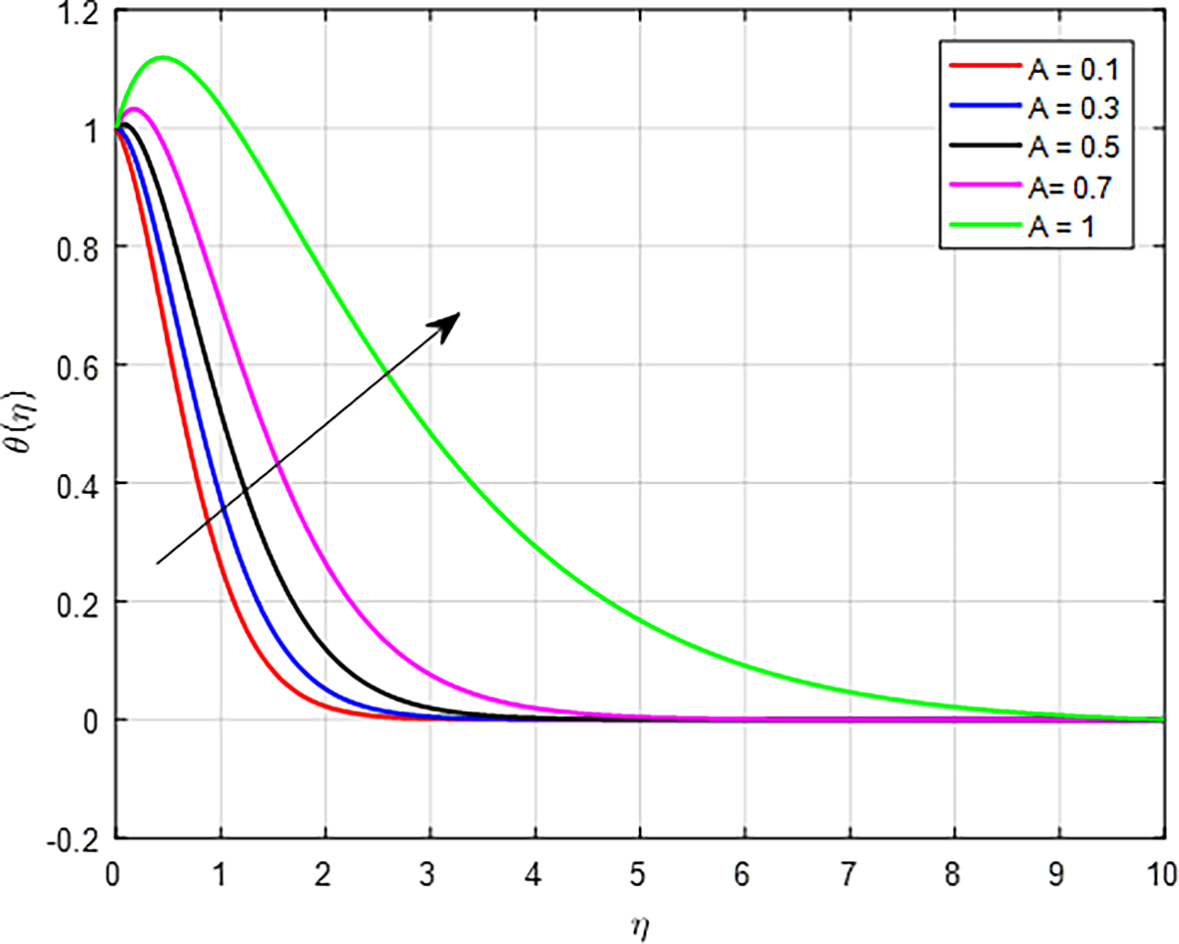
Temperature with *A*.

**Figure 15.**
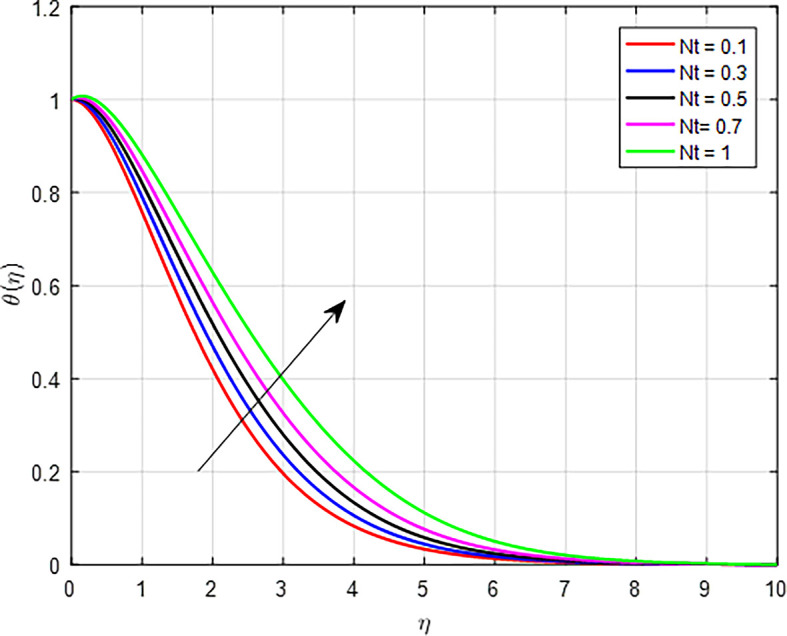
Temperature with *Nt*.

**Figure 16.**
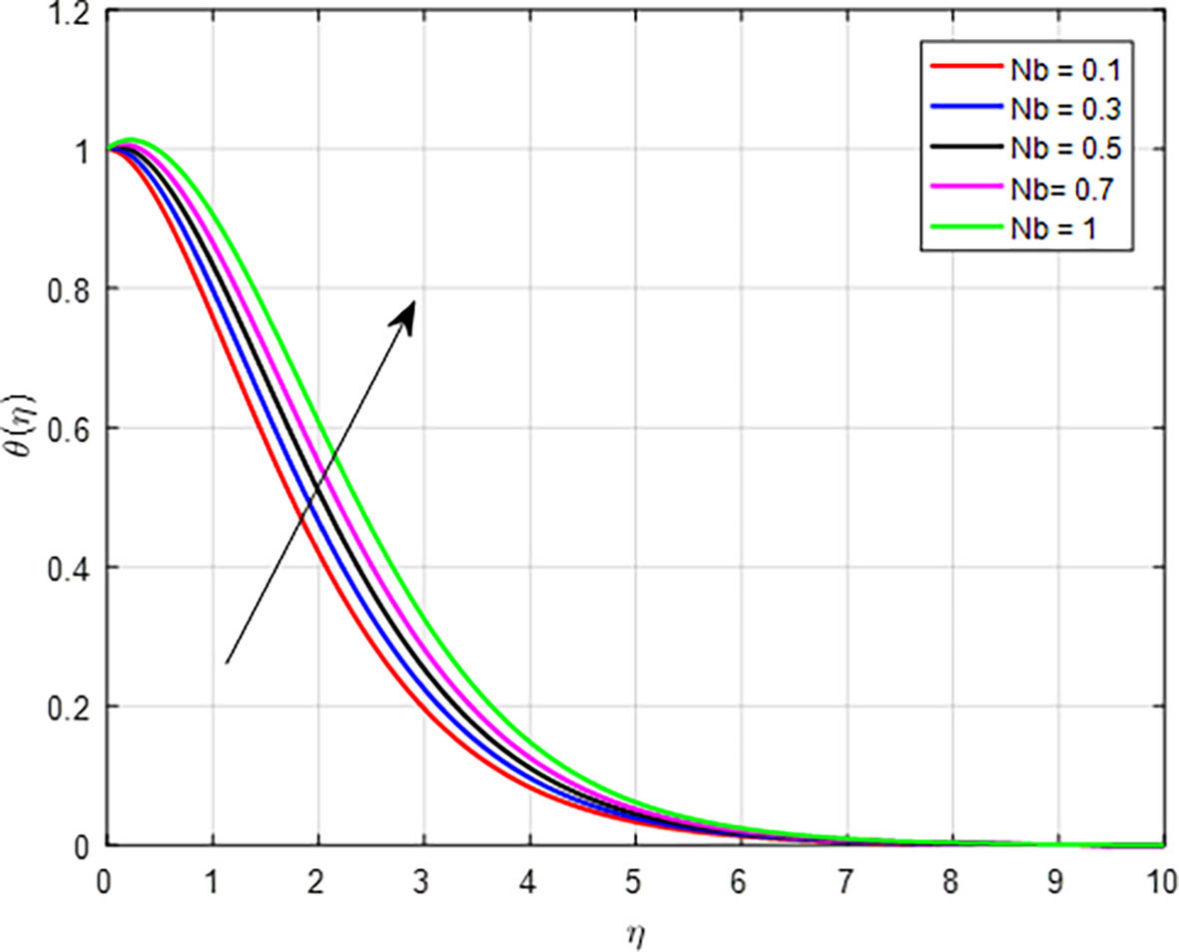
Temperature with *Nb*.

**Figure 17.**
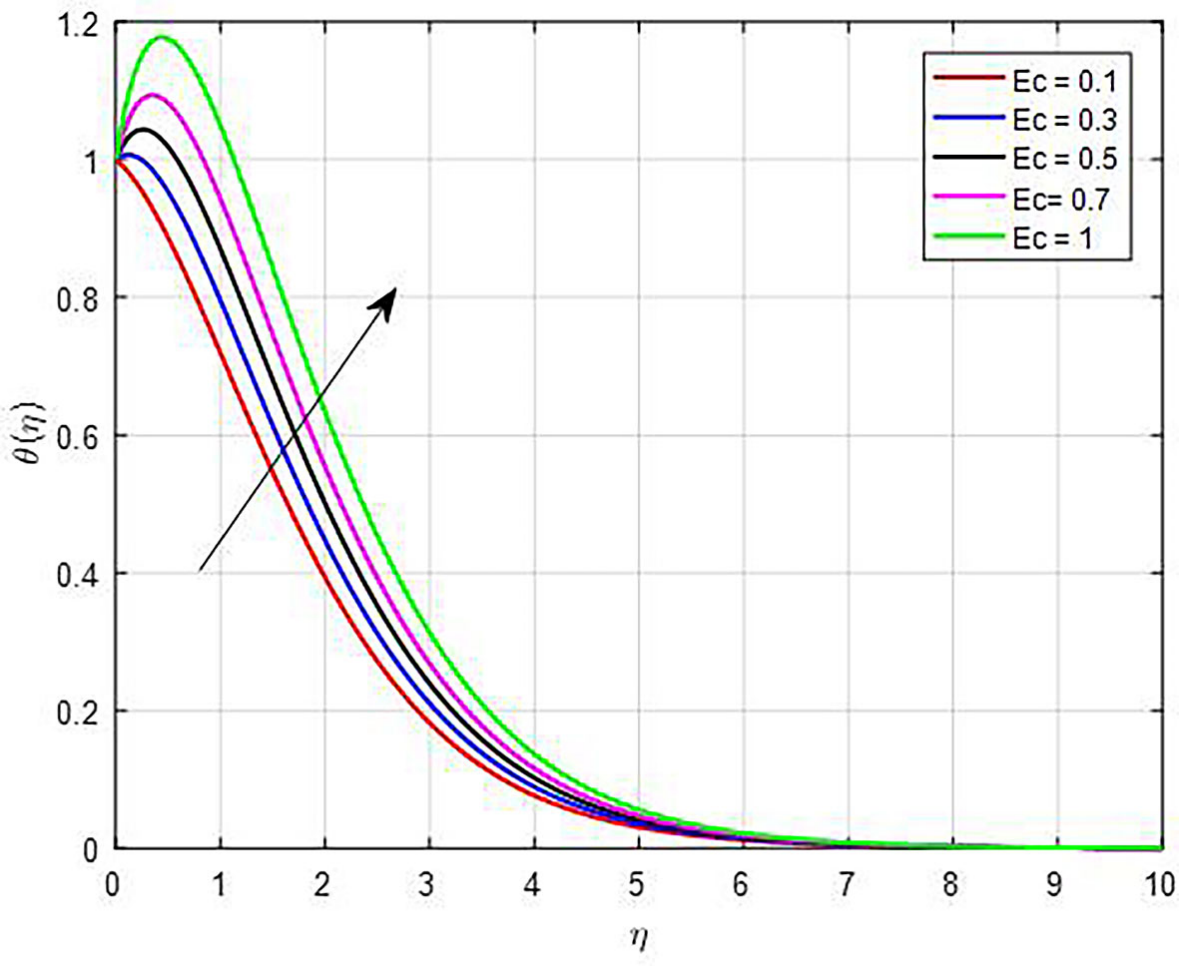
Temperature with *Ec*.

**Figure 18.**
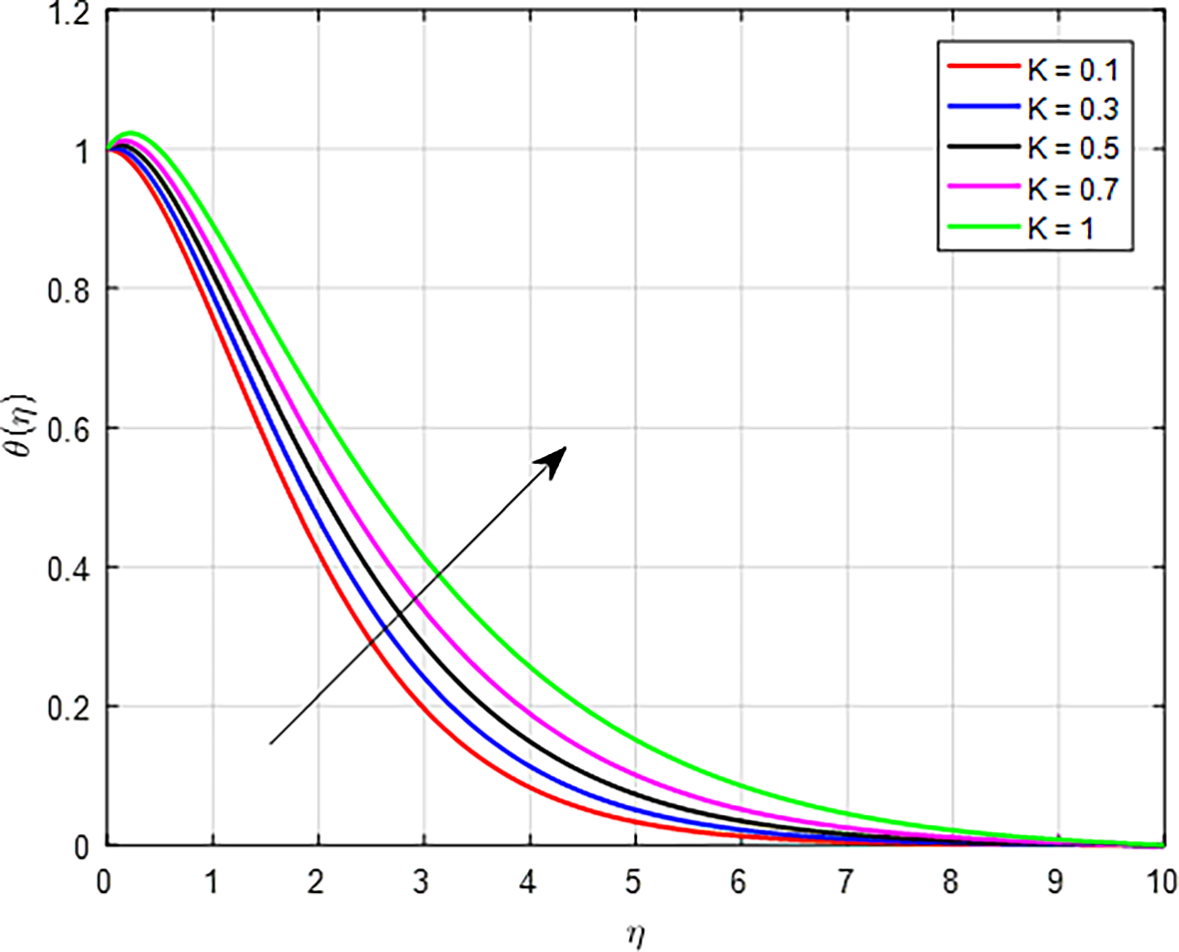
Temperature with *K*.

**Figure 19.**
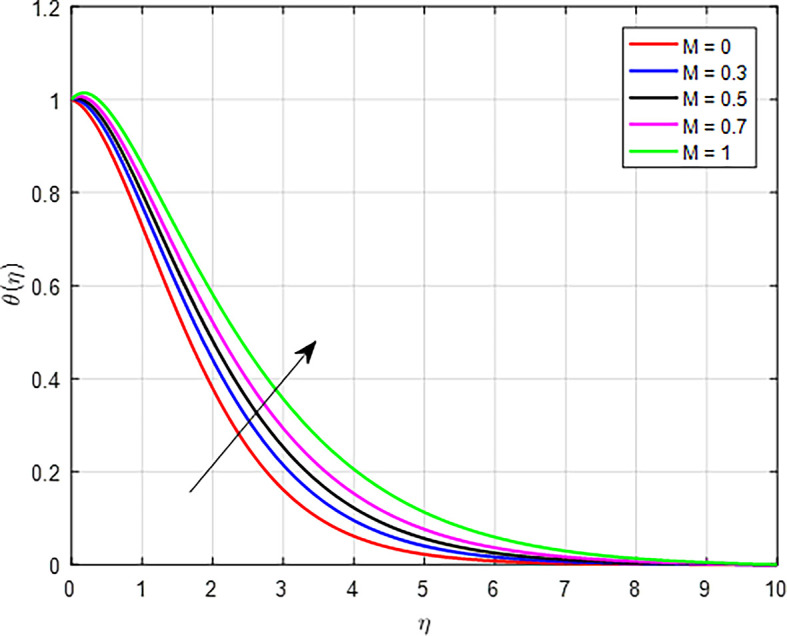
Temperature with *M*.

**Figure 20.**
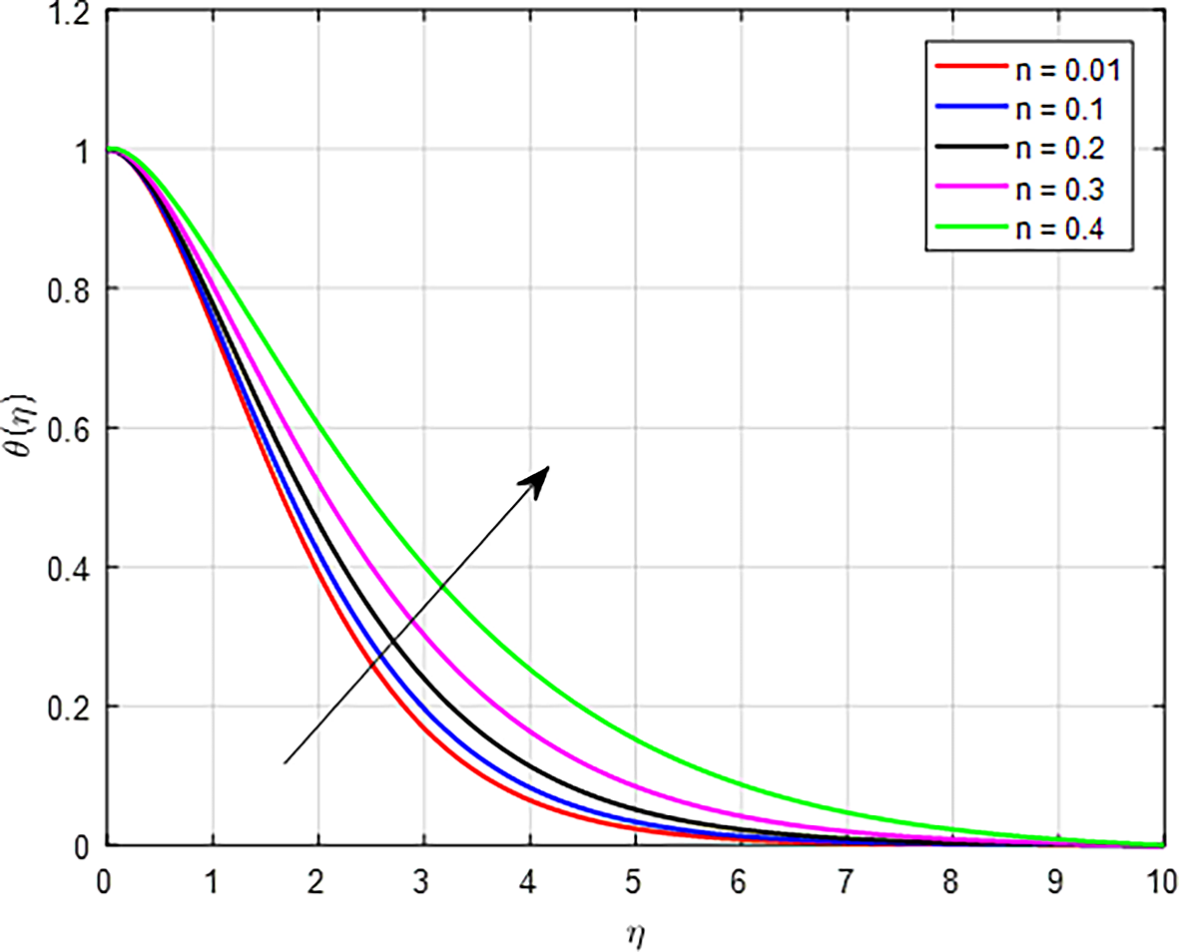
Temperature with *n*.

**Figure 21.**
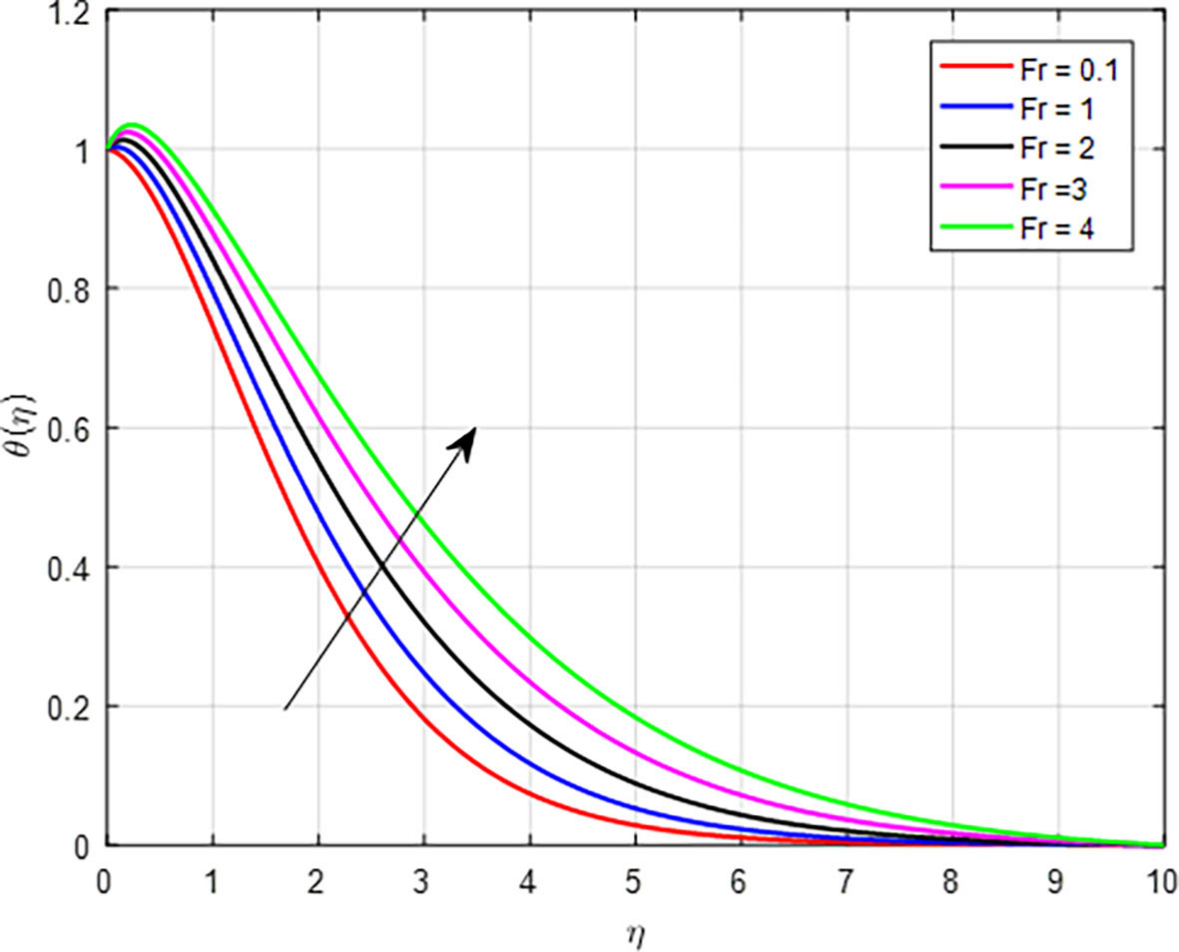
Temperature with *Fr*.

**Figure 22.**
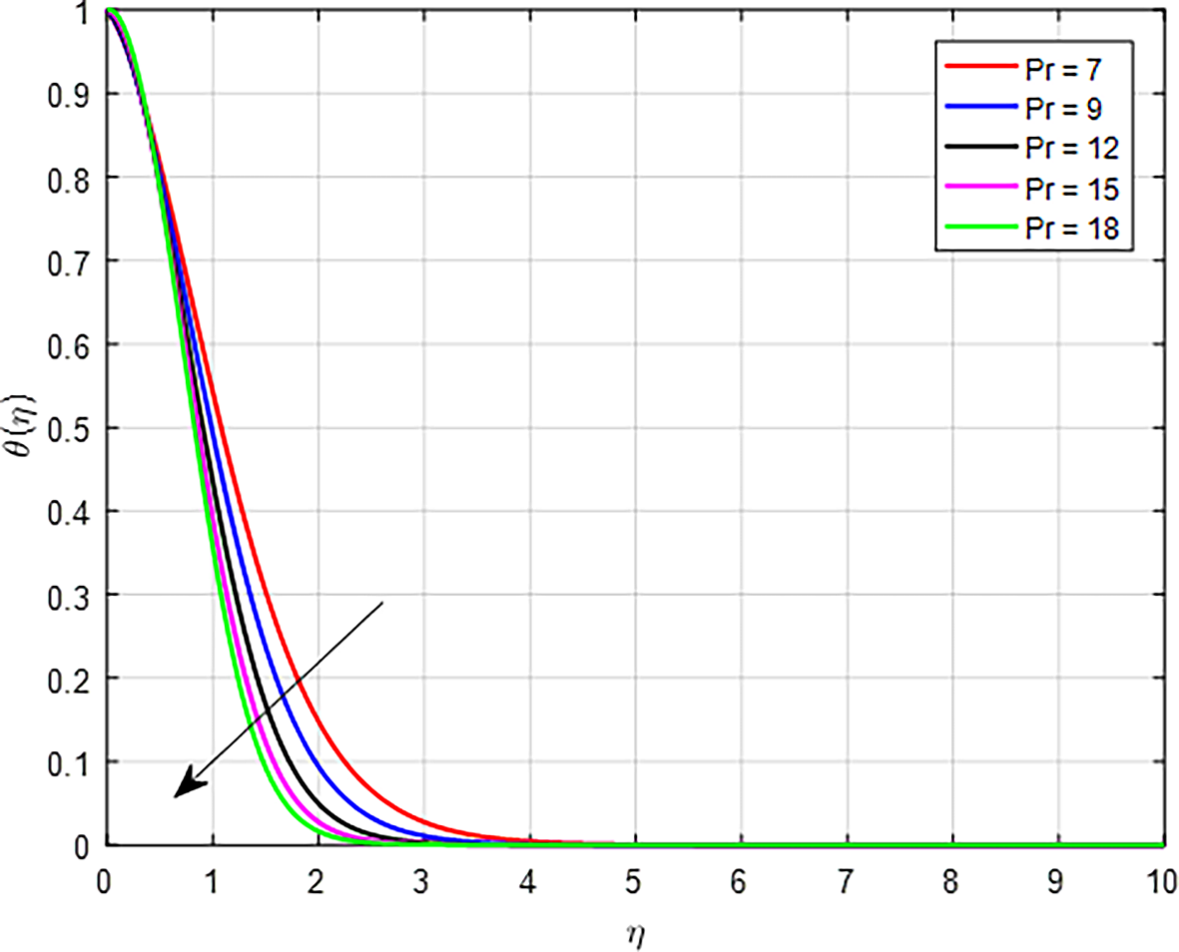
Variation of temperature with *Pr*.

**Figure 23.**
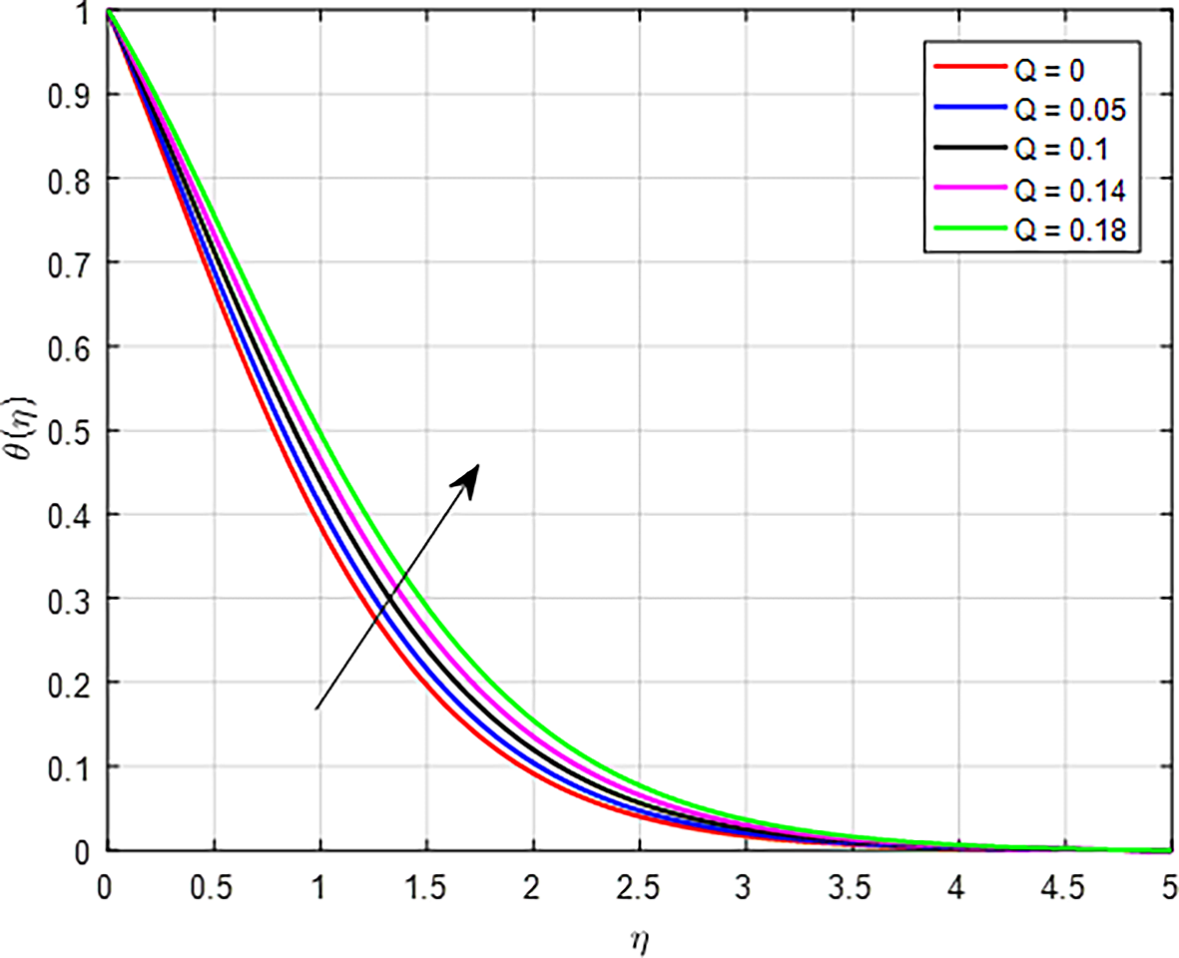
Temperature with *Q*.

**Figure 24.**
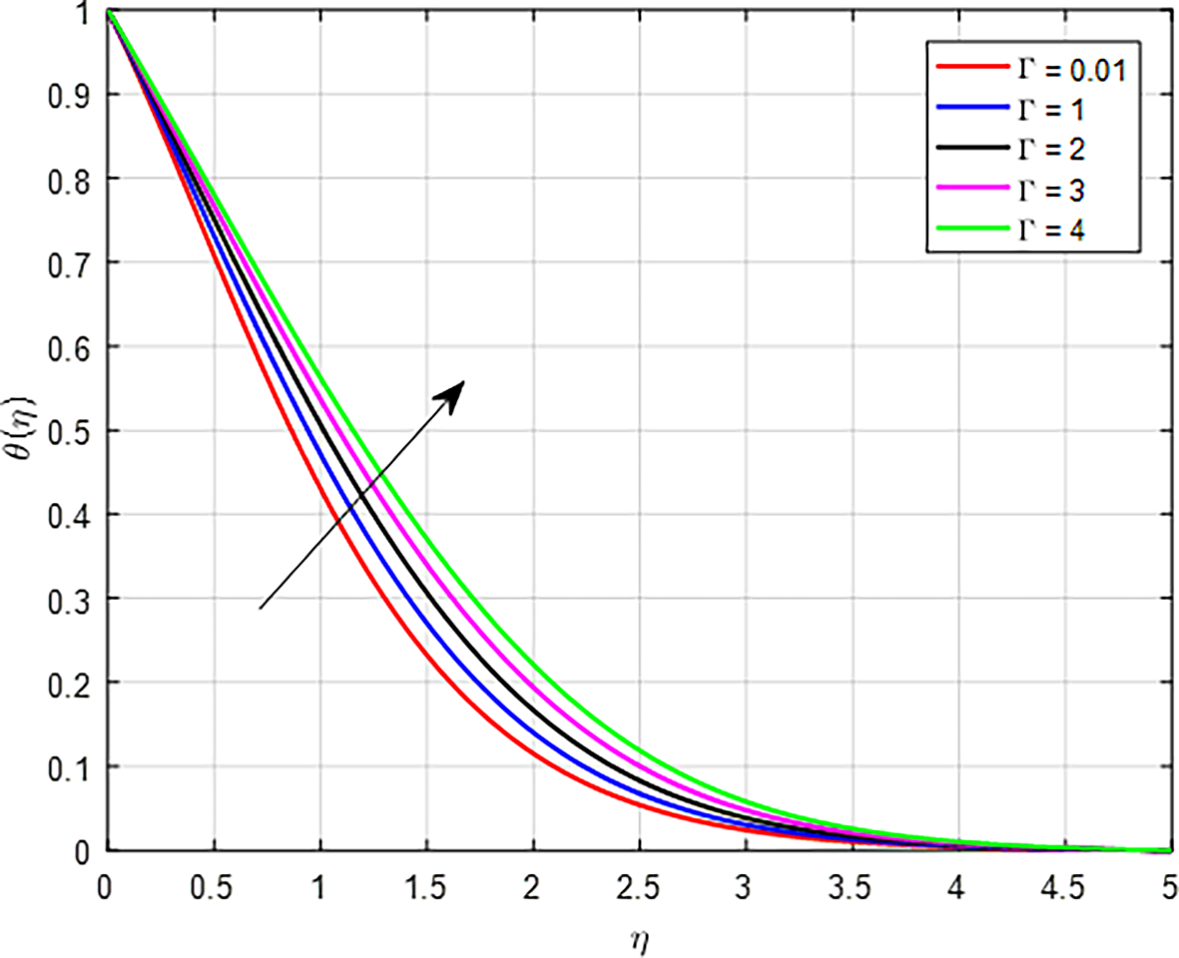
Temperature with Γ.

**Figure 25.**
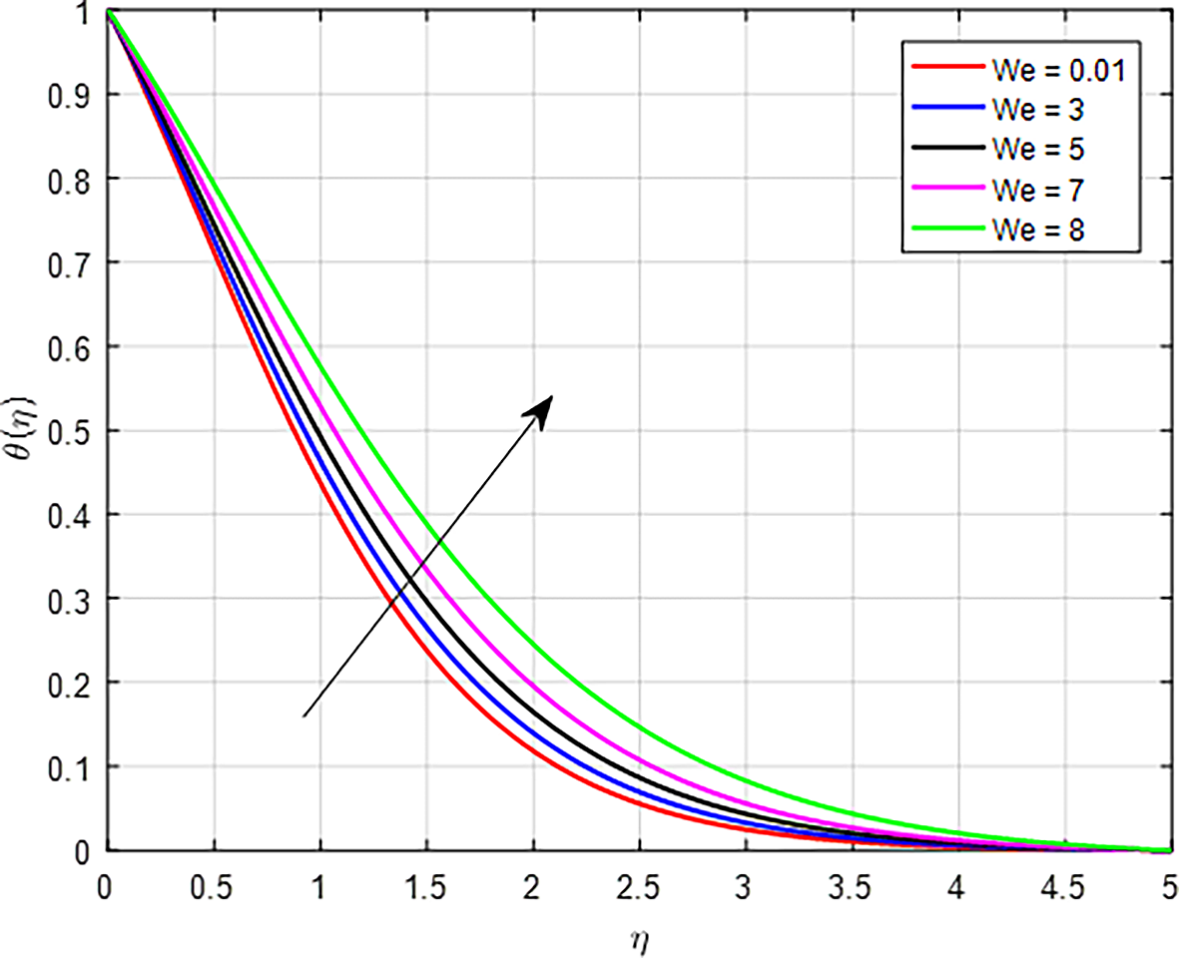
Temperature with *We*.

**Figure 26.**
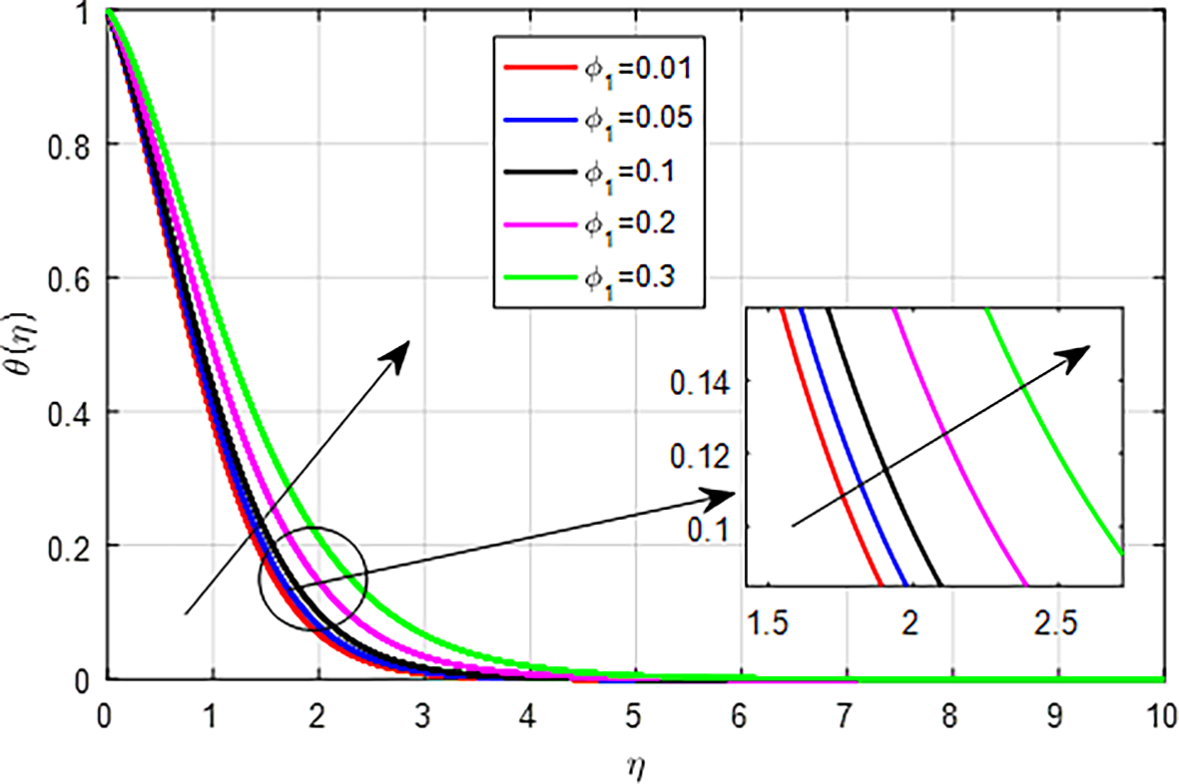
Temperature with *ϕ*_1_.

**Figure 27.**
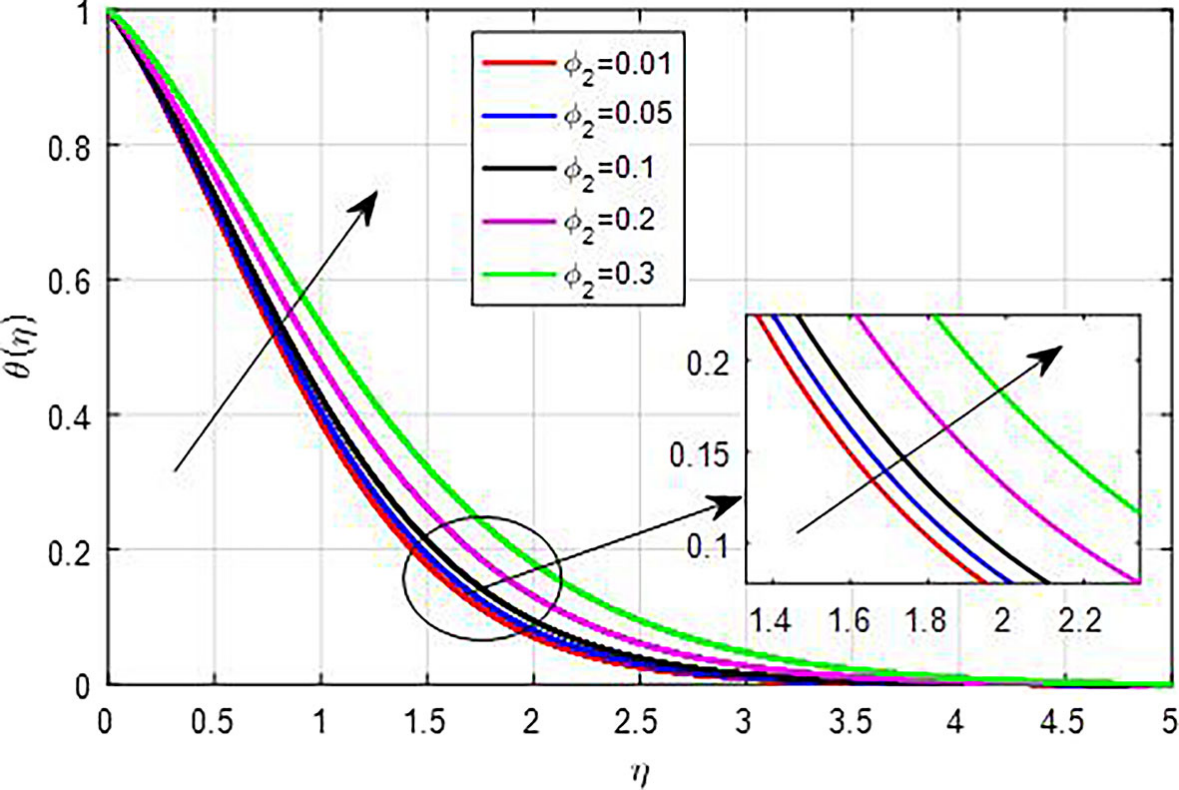
Temperature with *ϕ*_2_.

**Figure 28.**
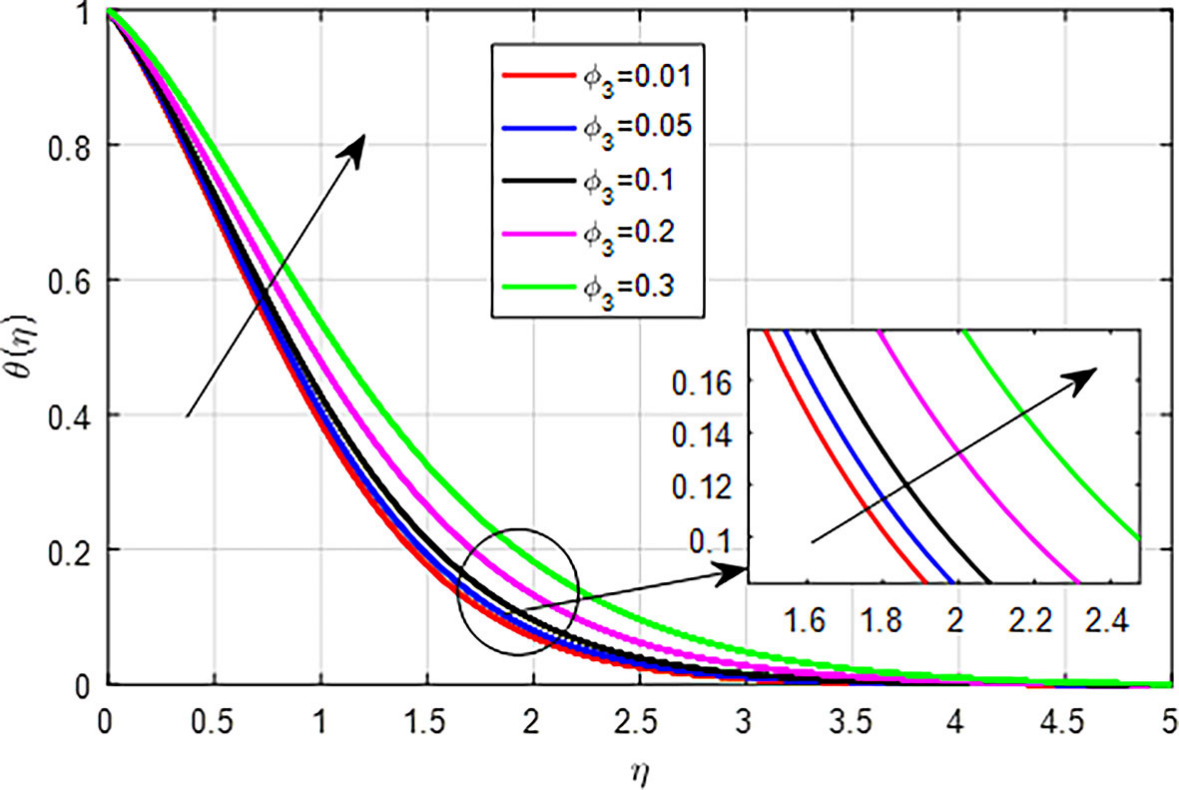
Variation of temperature with *ϕ*_3_.

**Figure 29.**
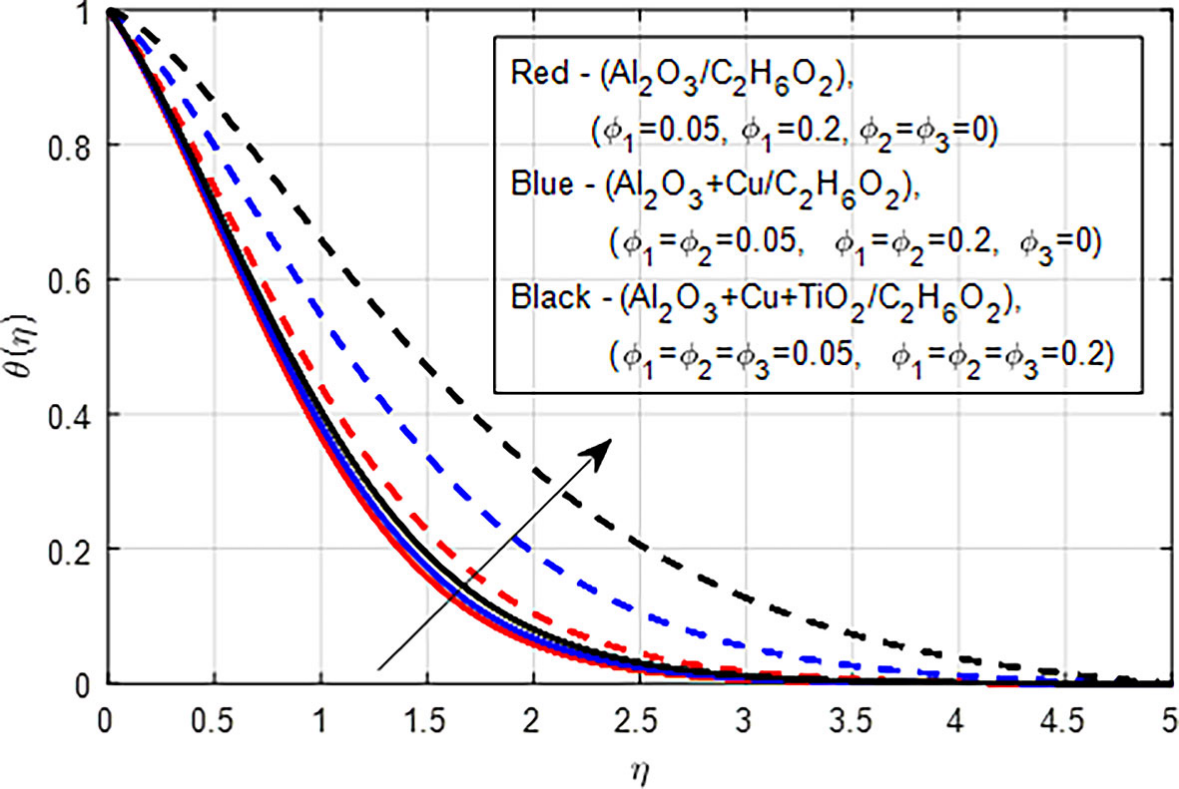
Temperature with *ϕ*_1_, *ϕ*_2_, *ϕ*_3_.

The effects of the nonlinear thermal radiation parameter ( *R*) on fluid temperatures are illustrated in Figure 12. When the values of nonlinear thermal radiation increase, the temperature profile in a fluid or material can also increase. In materials where radiation is a primary mode of heat transfer, an increase in nonlinear thermal radiation means that more energy is available to heat the material. This can lead to higher thermal gradients within the material, resulting in an overall increase in the temperature profile. Figure 13 depicts the thermal distribution against the temperature ratio $\left(\theta_{w}\right)$ . The temperature ratio often refers to the ratio of the temperature of a fluid to that of a boundary or a reference temperature. An increased temperature ratio implies a greater difference between the fluid temperature and a reference or surrounding temperature. This enhanced temperature gradient can lead to more efficient heat transfer from the hotter regions to the cooler ones, resulting in higher temperatures throughout the fluid or material. When the values of the unsteady parameter increase, the temperature profile in a fluid or material can also increase due to several interrelated mechanisms, as shown in Figure 14. The unsteady parameter often reflects the importance of transient effects relative to steady-state conditions, such as in heat transfer or fluid dynamics. An increase in the unsteady parameter can lead to a higher temperature profile through enhanced energy transport, rapid response to thermal gradients, and increased effective thermal conductivity. These factors illustrate the critical role that unsteady conditions play in influencing the thermal behavior of fluids and materials. Figure 15 present the effect of the thermophoresis diffusion on the temperature profile. As the thermophoresis diffusion parameter increases, particles within the fluid experience a stronger thermophoresis force, which causes them to migrate from regions of lower temperature to regions of higher temperature. This movement can lead to a concentration of particles in hotter areas, effectively increasing the local temperature in those regions. The influence of Brownian motion on the temperature distribution, as shown in Figure 16. As the Brownian diffusion parameter increases, the microscopic motion of particles (due to Brownian motion) becomes more significant. This enhanced movement leads to improved mixing within the fluid, promoting more uniform temperature distribution and potentially raising the average temperature profile by allowing hotter regions to mix with cooler ones more effectively. As we can see from the temperature distribution graphs in Figure 17, the temperature functions get better as the Eckert number ( *Ec*) increases in value. The Eckert number ( *Ec*) is a dimensionless parameter that characterizes the relative importance of kinetic energy to thermal energy in a fluid flow, particularly in situations involving heat transfer. As the Eckert number rises, the kinetic energy associated with the flow increases. This kinetic energy can contribute to the overall thermal energy of the fluid, leading to higher temperatures, especially if the kinetic energy is converted into internal energy during viscous dissipation. The significance of the porous permeability media parameter ( *K*) on temperature profiles is depicted in Figure 18. When the porous permeability parameter ( *K*) increases, the temperature profile in a fluid flowing through a porous medium can also increase. In porous media, higher permeability often means a more interconnected pore structure. This can provide a larger surface area for heat exchange between the fluid and the solid matrix of the medium, enhancing heat transfer efficiency and contributing to a higher temperature profile. As Figure 19 illustrate, the temperature increases as the magnetic field parameter ( *M)* increases. As the magnetic field strength increases, it can exert a damping effect on the fluid flow. This damping can reduce the velocity of the fluid, leading to increased viscous heating. The kinetic energy of the fluid being converted into thermal energy due to viscous dissipation results in a higher temperature profile. Figure 20 shows a graph of the temperature profile as a result of the increasing the power law index (n) . An increase in the power law index parameter (n) , can lead to a higher temperature profile through increased viscosity and viscous heating, reduced flow velocity resulting in enhanced energy dissipation, altered heat transfer characteristics, greater thermal energy retention, improved mixing and flow stability, and interactions with temperature gradients. These factors collectively illustrate how non-Newtonian fluid behavior influences thermal dynamics within the flow. The effect of the Forchheimer number ( *Fr*) on the temperature distribution, demonstrated in Figure 21. When the Forchheimer number increases, it indicates that inertial forces are becoming more significant compared to viscous forces. As inertial forces become more dominant, the mixing and transport of thermal energy are enhanced. The increased movement of the fluid helps distribute heat more uniformly, resulting in a higher average temperature within the porous medium. As in Figure 22, an increase in the Prandtl number indicates that the kinematic viscosity is relatively high compared to thermal diffusivity. This means that momentum diffuses more slowly than heat. Higher viscosity can lead to reduced flow velocities, which can decrease the fluid’s ability to carry heat away from hot regions, resulting in a less effective temperature distribution. Figure 23 show the effect of the heat generation on the temperature profile. Increasing the rate of heat generation results in a direct increase in thermal energy, leading to higher temperatures in the fluid or material. This effect is reinforced by heat transfer mechanisms, thermal gradients, and material properties, all contributing to a rising temperature profile. As a result, regions closer to the heat source experience significant temperature increases, influencing the overall thermal behavior of the system. As in Figure 24 when the variable thermal conductivity parameter (Γ) increases, the temperature profile generally increases due to enhanced heat transfer capabilities of the material or fluid. As heat is conducted more effectively, the temperature can rise in regions further away from the heat source, resulting in an increased overall temperature profile. As the Weissenberg number ( *We*) increases for a tangent hyperbolic fluid, the fluids viscoelastic properties become more pronounced, leading to increased energy storage and viscous dissipation, as seen Figure 25. This results in enhanced heat generation, steeper temperature gradients, and improved heat transfer characteristics, collectively contributing to an increase in the temperature profile. The balance between heat generation and dissipation is crucial in determining the extent of this temperature rise. As the nanoparticle volume fraction $\left(\phi_{1},\phi_{2},\phi_{3}\right)$ increases, the overall temperature profile of the nanofluid tends to rise due to enhanced thermal conductivity, increased heat generation through viscous dissipation, reduced specific heat capacity, improved thermal boundary layer effects, and enhanced convection, as seen Figures 26, 27, 28. Collectively, these factors contribute to more effective heat transfer and distribution, resulting in higher temperatures within the nanofluid. Figure 29 show a graph of the comparison of the nanofluid ( *Al*_2_ *O*_3_ */C*_2_ *H*_6_ *O*_2_), hybrid nanofluid ( *Al*_2_ *O*_3_ − *Cu/C*_2_ *H*_6_ *O*_2_), and ternary hybrid nanofluid ( *Al*_2_ *O*_3_ − *Cu* − *TiO*_2_ */C*_2_ *H*_6_ *O*_2_) on the temperature distribution. As the volume fraction of nanoparticles $\left(\phi_{1},\phi_{2},\phi_{3}\right)$ increases in nanofluids, hybrid nanofluids, and ternary hybrid nanofluids, the temperature profile increases due to improved thermal conductivity, enhanced viscous dissipation, and better convective heat transfer. The combination of these factors leads to higher localized and overall average temperatures in the fluid. Generally, while all three types of fluids (nanofluids, hybrid nanofluids, and ternary hybrid nanofluids) show increased temperature profiles and enhanced heat transfer capabilities with higher nanoparticle concentrations, ternary hybrid nanofluids generally provide the best performance in terms of thermal conductivity and heat transfer efficiency. This is due to their ability to leverage the synergistic effects of multiple types of nanoparticles, leading to superior thermal management in various applications.

### Concentration characteristic

**Figure 30.**
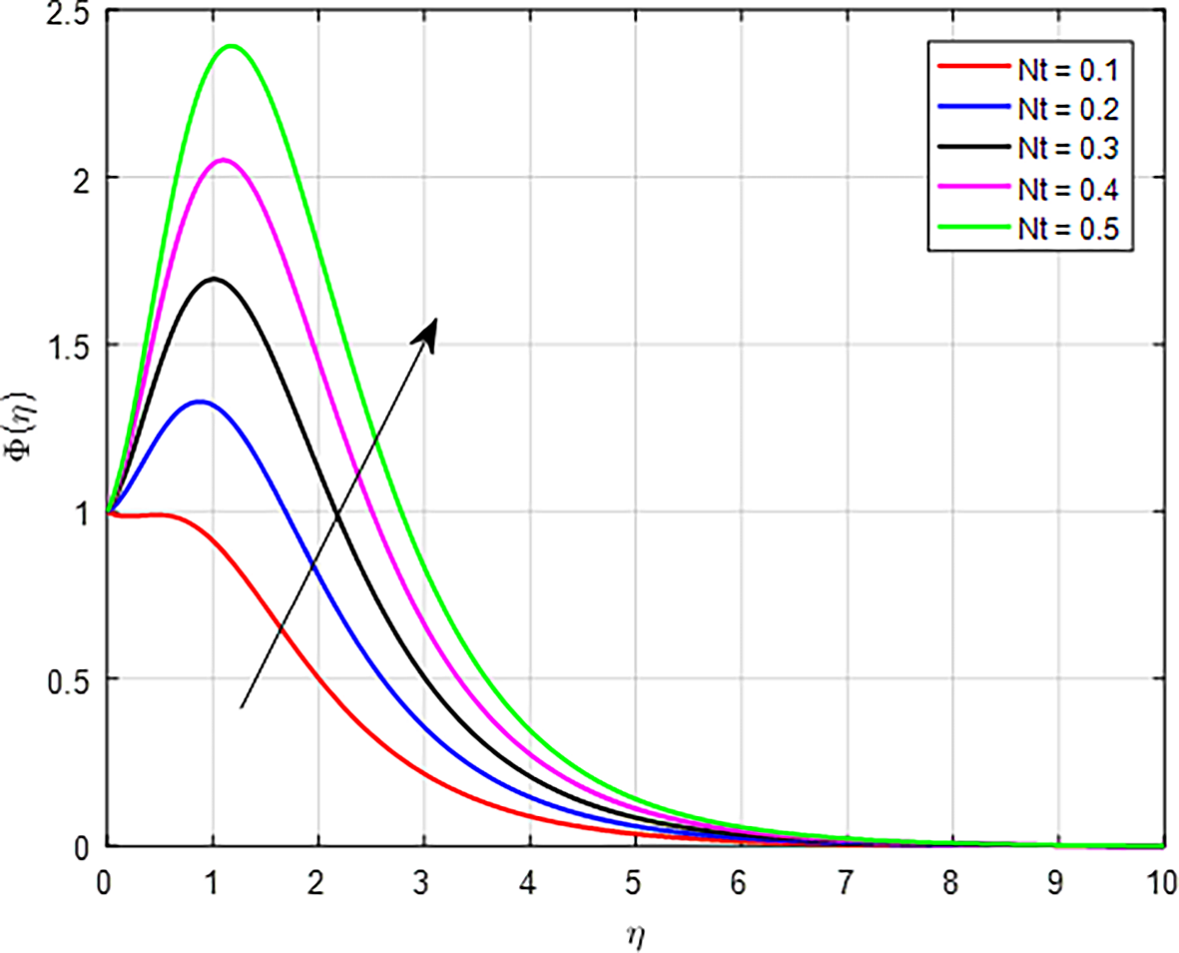
Concentration with *Nt*.

**Figure 31.**
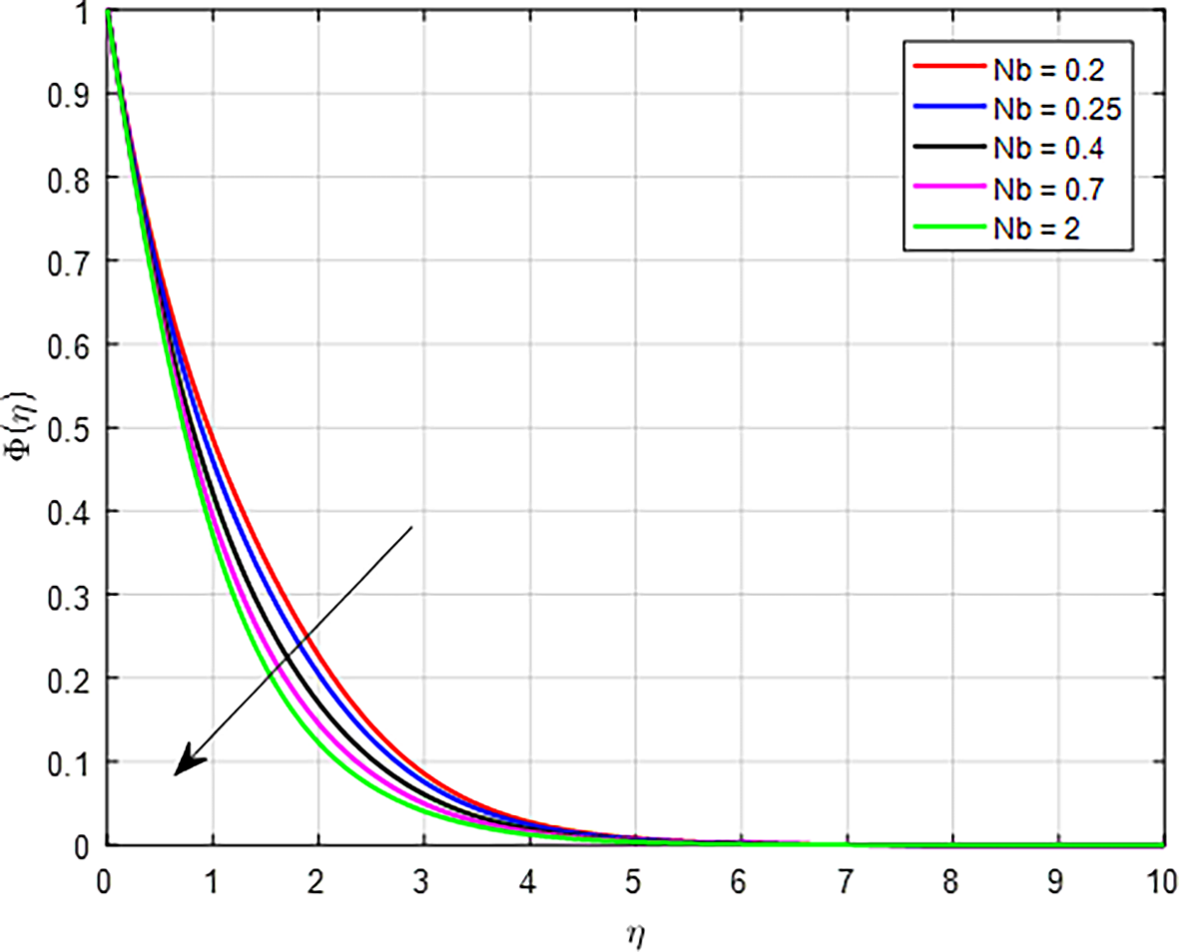
Concentration with *Nb*.

**Figure 32.**
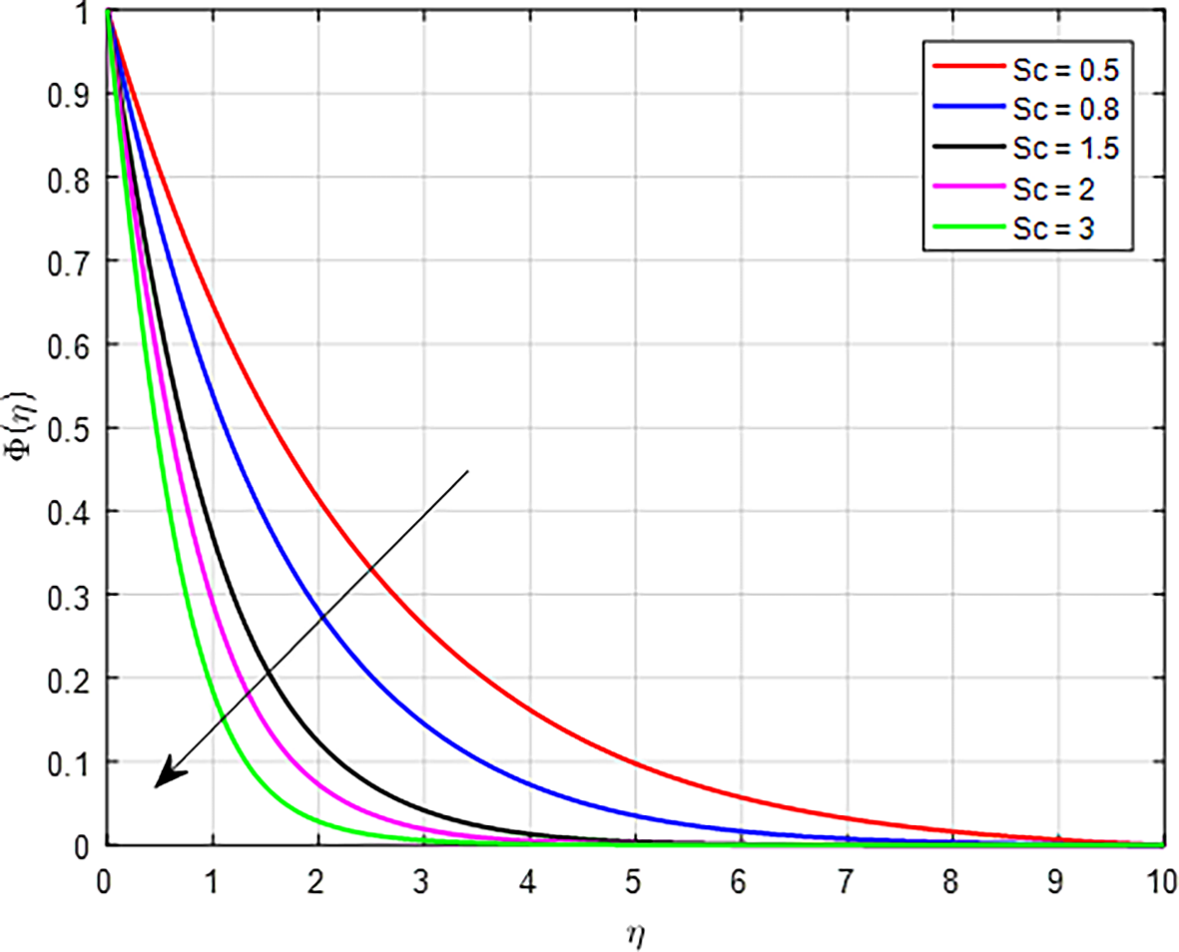
Concentration with *Sc*.

**Figure 33.**
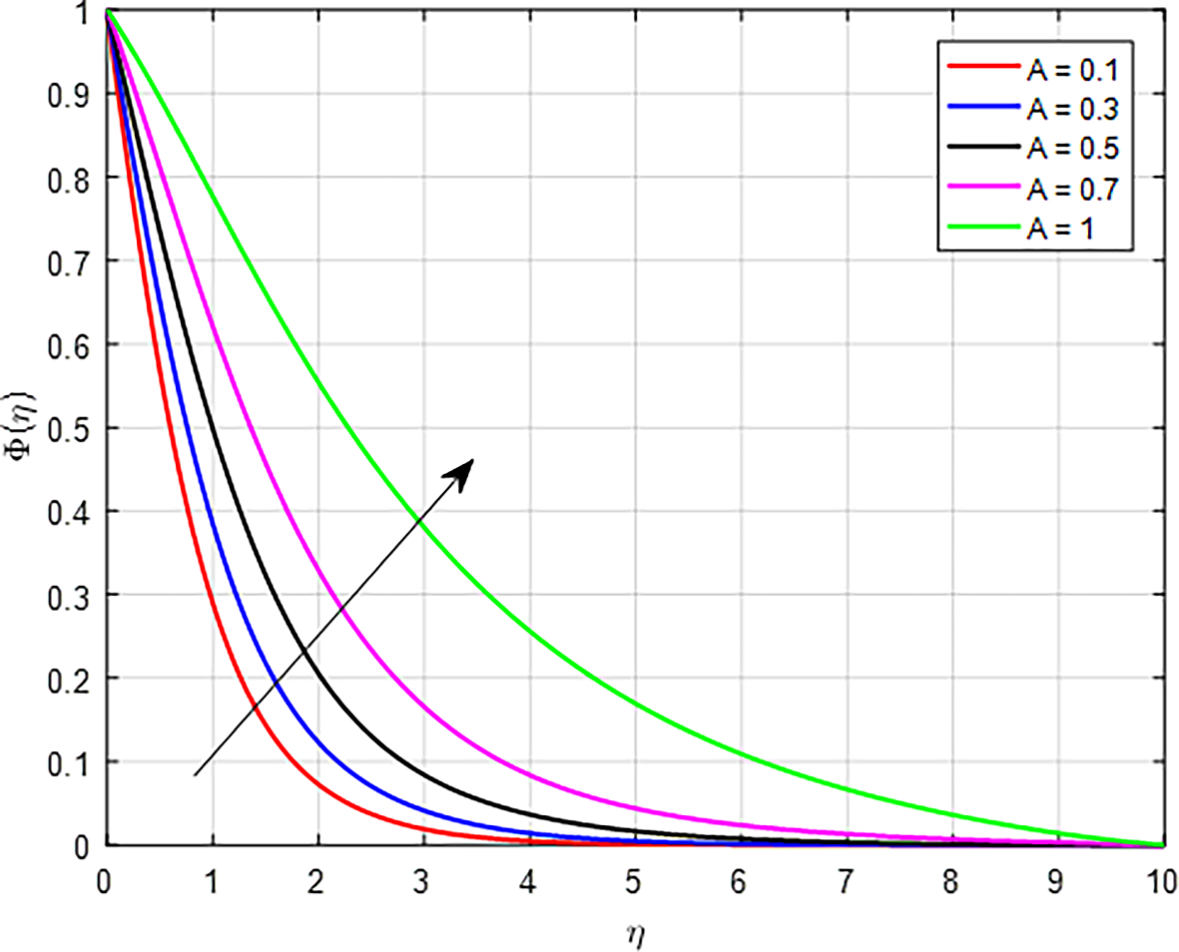
Concentration with *A*.

**Figure 34.**
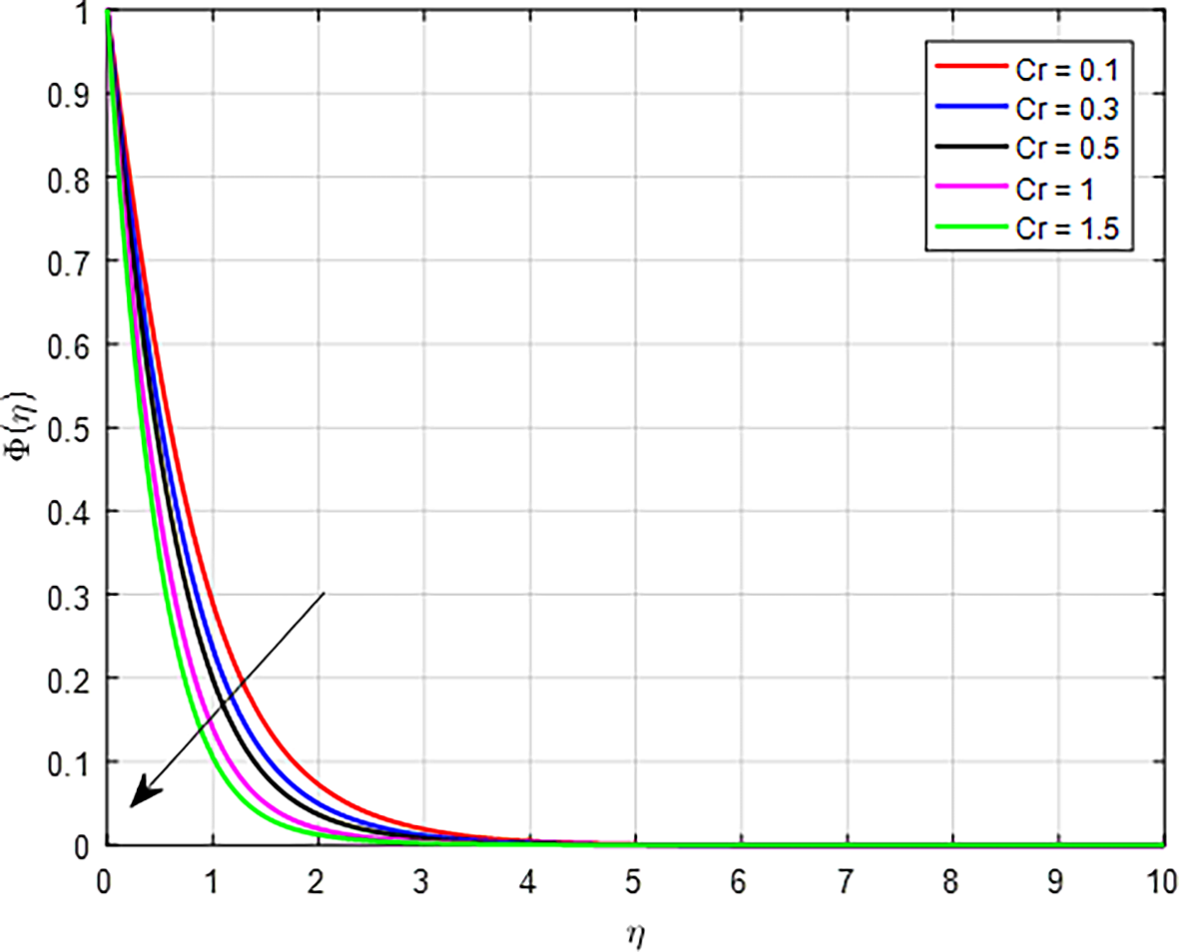
Concentration with *Cr*.

**Figure 35.**
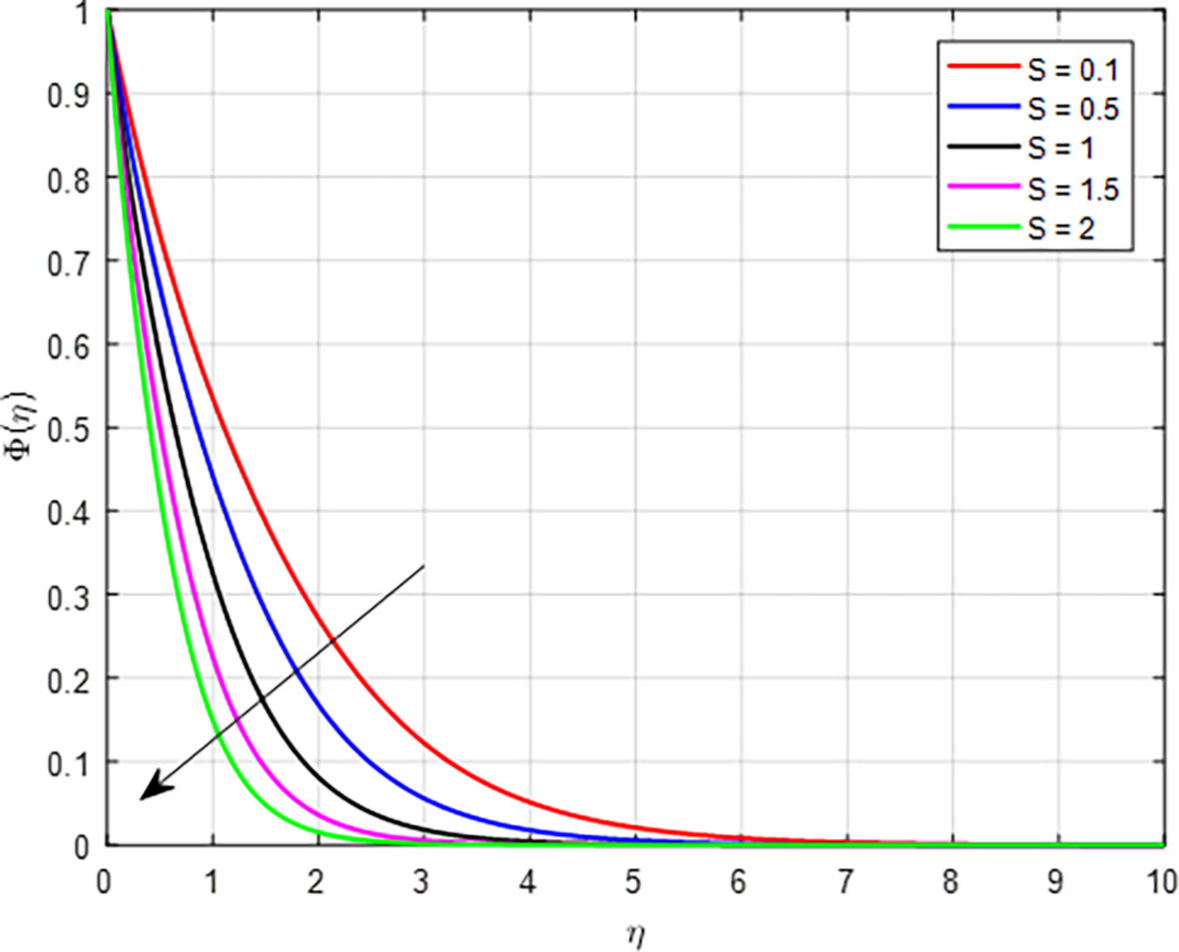
Concentration with *S*.

The effect of the thermophoresis diffusion parameter ( *Nt*) on the concentration profile, as illustrated in Figure 30. As the thermophoresis parameter increases, the transport of particles within the fluid becomes more effective. This enhanced transport allows for a greater number of particles to move towards and concentrate in regions of higher temperature, further increasing the concentration profile. Figure 31 illustrate the impact of the Brownian diffusion Parameter ( *Nb*) on the concentration profile. When the Brownian diffusion parameter ( *Nb*) increases, it indicates a stronger influence of Brownian motion on the transport of particles suspended in a fluid. As Brownian diffusion increases, particles undergo more vigorous random motion. This enhanced diffusion can cause particles to spread out more rapidly within the fluid, leading to a decrease in local concentration as particles move away from regions of higher concentration. Figure 32 depict the effect of the Schmidt number ( *Sc*) on the concentration profile. The Schmidt number ( *Sc*) is a dimensionless parameter that characterizes the relative importance of momentum diffusion (viscous effects) to mass diffusion (concentration effects) in a fluid. When the Schmidt number increases, it typically indicates that momentum diffusion is more significant compared to mass diffusion. When momentum diffusion dominates, it can lead to smaller concentration gradients because the particles are less able to respond to local variations in concentration. This smaller gradient reduces the driving force for diffusion, causing a decrease in the concentration profile over time as the particles do not accumulate effectively. Figure 33 show the effect of the unsteady parameter ( *A*) on the concentration profile. When the unsteady parameter ( *A*) increases, it typically indicates that transient effects in the system are becoming more significant compared to steady-state effects. The unsteady parameter affects how particles are transported in the fluid. With higher values of the unsteady parameter, the transport mechanisms (like diffusion and convection) can operate differently, potentially leading to increased mixing or accumulation of particles in certain areas, thereby increasing the concentration profile. Figure 34 present that when the chemical reaction parameter increases, the solute’s boundary layer thickness decreases. In reactions where the concentration of a reactant decreases as it transforms into products, an increase in the chemical reaction parameter means that the reactants are being converted to products more rapidly. This results in a lower concentration of the reactant remaining in the system, causing the overall concentration profile to decline. Figure 35 depict the effect of suction parameter ( *S*) on the concentration profile. As the values of the suction parameter increase, the concentration profile decreases due to the removal of fluid that carries solutes, leading to a reduction in local concentration. This effect is further amplified by the dynamics of the concentration boundary layer and enhanced mass transfer away from the surface that prioritize the extraction of fluid over the retention of solutes. Consequently, the overall concentration profile in the fluid becomes lower with increased suction.

### Impacts of parameters on the coefficient of skin friction, local Nusselt and Sherwood number

In Table 3, we present the numerical results for the skin friction (CfxRe12), the Nusselt number (NuxRe−12) , and the Sherwood number (ShxRe−12) . These key parameters are evaluated using various dimensionless parameters for ternary hybrid nanofluids. As the Weissenberg number increases, the skin friction coefficient decreases due to the dominance of elastic effects leading to lower wall shear rates and potentially more stable flow and the Sherwood number decreases as the flow becomes less turbulent and diffusion rates drop, resulting in less effective mass transfer. Also, the Nusselt number increases because the tangent hyperbolic fluid properties can still enhance heat transfer through improved convective mechanisms, allowing for better thermal management despite the reduced mixing and diffusion. The increase in the unsteady parameter leads to a reduction in the effectiveness of both mass and heat transfer, resulting in lower values of the skin friction coefficient, Sherwood number, and Nusselt number. Skin friction coefficient tends to increase with higher magnetic field parameter ( *M*), Forchheimer number ( *Fr*), and velocity ratio parameter ( *λ*), while it decrease with Weissenberg number ( *We*), unsteady parameter ( *A*), and power law index ( *n*). Nusselt numbers decrease as unsteady parameter ( *A*), magnetic field parameter ( *M*), Forchheimer number ( *Fr*), Eckert number ( *Ec*), and velocity ratio parameter ( *λ*) values increase, whereas they increase with the increase in the values of Weissenberg number ( *We*), power law index ( *n*), variable thermal conductivity (Γ), and nonlinear thermal radiation ( *R*). Increases with enhanced thermal conductivity, allowing for better heat transfer. Also, increases as nonlinear thermal radiation enhances heat transfer, particularly at high temperatures. Sherwood numbers increase with the increased values of chemical reaction ( *C _r_*) and velocity ratio parameter ( *λ*), whereas they decrease with the increasing values of Weissenberg number ( *We*), unsteady parameter ( *A*), and power law index (n) . Increases as the chemical reaction can enhance concentration gradients, improving mass transfer.

**Table 3.** Mathematical data of skin friction coefficient, Nusselt number and Sherwood number of some values of parameters for *A* = 0 *.*1 *, Nb* = 0 *.*5 *, Nt* = 0 *.*1 *,* $\theta_{w}$ = 1 *.*2 *, Q* = 0 *.*01 *, K* = 0 *.*1 *, λ* = 1 *,* Γ = 0 *.*3 *, We* = 0 *.*5 *, Cr* = 0 *.*1 *, R* = 1 *.*5 *, Ec* = 0 *.*2 *, Pr* = 7 *, M* = 0 *.*2 *, Sc* = 2 *, n* = 0 *.*1 *, S* = 0 *.*1 *, Fr* = 0 *.*3, $\phi_{1}=\phi_{2}=\phi_{3}=0.05$ .

*We*	A	M	ER	n	*λ*	*R*	Γ	*Ec*	*Cr*	CfxRe12	NuxRe−12	ShxRe−12
0.1										1.762244	0.590423	1.062417
1.5										1.688485	0.593556	1.054830
3										1.591519	0.595070	1.043963
	0.1									1.742281	0.591422	1.060418
	0.3									1.703993	0.288018	0.848307
	0.5									1.671153	0.046494	0.662183
		0								1.662063	0.691278	1.060752
		0.7								1.930985	0.358756	1.060005
		1.5								2.203555	0.028154	1.060372
			0.1							1.673295	0.652511	1.060683
			0.5							1.808894	0.532523	1.060228
			1							1.966347	0.393666	1.060034
				0.1						1.742281	0.591422	1.060418
				0.3						1.443509	0.634711	1.031621
				0.5						1.709855	0.634365	0.971353
					1					1.742281	0.591422	1.060418
					1.1					1.980330	0.469779	1.100296
					1.3					2.482471	0.154472	1.182047
						0.1				1.742281	0.019870	1.091851
						1				1.742281	0.392378	1.067107
						2				1.742281	0.788465	1.055787
							0.1			1.742281	0.530495	1.064099
							1			1.742281	0.758082	1.050365
							2			1.742281	0.917032	1.040816
								0.1		1.742281	0.957248	1.037816
								0.2		1.742281	0.591422	1.060418
								0.3		1.742281	0.226086	1.083006
									0.1	1.742281	0.591422	1.060418
									0.3	1.742281	0.577866	1.237084
									0.5	1.742281	0.567410	1.394140

Table 4 explores the effects of $\phi_{1}$ , $\phi_{2}$ , and $\phi_{3}$ on the skin friction coefficient, Nusselt number, and Sherwood number for ternary hybrid nanofluids, hybrid nanofluids, and mono nanofluids. The results indicate that increasing the nanoparticle volume fractions $\left(\phi_{1},\phi_{2},\phi_{3}\right)$ leads to enhancements in the skin friction coefficient, local Nusselt number, and local Sherwood number. The skin friction coefficient is a measure of the frictional resistance experienced by a fluid flowing over a surface. With increased nanoparticle concentration, the skin friction coefficient generally increases, indicating greater resistance due to higher fluid density and viscosity. As nanoparticle concentration rises, both the Nusselt and Sherwood numbers rise, indicating improved heat and mass transfer efficiency, with ternary hybrid nanofluids being the most effective in enhancing heat transfer and thermal conductivity, along with an increase in skin friction coefficient.

**Table 4.** Mathematical data of skin friction coefficient, Nusselt number and Sherwood number of some values of parameters for *A* = 0 *.*1 *, Nb* = 0 *.*5 *, Nt* = 0 *.*1 *,* $\theta_{w}$ = 1 *.*2 *, Q* = 0 *.*01 *, K* = 0 *.*1 *, λ* = 1 *,* Γ = 0 *.*3 *, We* = 0 *.*5 *, Cr* = 0 *.*1 *, R* = 1 *.*5 *, Ec* = 0 *.*2 *, Pr* = 7 *, M* = 0 *.*2 *, Sc* = 2 *, n* = 0 *.*1 *, S* = 0 *.*1 *, Fr* = 0 *.*3.

$\phi_{1}$	$\phi_{2}$	$\phi_{3}$	CfxRe12	NuxRe−12	ShxRe−12
0.01	0	0	1.234185	0.802721	1.034962
0.08	0	0	1.486944	0.862325	1.035621
0.15	0	0	1.821320	0.921726	1.036584
0.01	0.01	0	1.261670	0.814131	1.035321
0.08	0.08	0	1.811703	0.964584	1.037424
0.15	0.15	0	2.715110	1.133760	1.039626
0.01	0.01	0.01	1.291004	0.824010	1.035627
0.08	0.08	0.08	2.213728	1.074752	1.039276
0.15	0.15	0.15	4.041513	1.403616	1.043548

The present study has significant real-world applications in various engineering and industrial fields. The enhanced heat transfer properties of tangent hyperbolic ternary hybrid nanofluids make them highly effective for cooling systems, such as electronic device cooling, heat exchangers, and thermal management in power plants. Additionally, their superior thermal conductivity and stability are beneficial in biomedical applications, including targeted drug delivery and hyperthermia treatments. The findings also have implications in polymer processing industries, where precise thermal control is required for extrusion and casting. Furthermore, these nanofluids can be utilized in aerospace and automotive industries for efficient thermal regulation and improved fuel efficiency. The insights gained from this research contribute to optimizing fluid flow and heat transfer processes in advanced engineering systems.

## Conclusion

This study investigates the unsteady MHD flow, heat transfer, and mass transfer characteristics of a tangent hyperbolic ternary hybrid nanofluid over a permeable stretching sheet in a Darcy-Forchheimer porous medium with variable thermal conductivity. The combined effects of viscous dissipation, nonlinear thermal radiation, Joule heating, Brownian motion, thermophoresis, and chemical reactions on the fluid’s thermal and flow behavior are analyzed. The governing equations are transformed into a system of ordinary differential equations using similarity transformations and solved numerically via the BVP5C method in MATLAB. The **BVP5C** solver provides high accuracy for stiff boundary value problems, leveraging adaptive mesh refinement and efficiently handling nonlinearities. Compared to finite difference methods, it offers faster convergence with minimal dependence on grid size. Graphical analysis is employed to illustrate the effects of these parameters on flow, heat, and mass transfer, providing valuable insights for researchers and engineers working on similar thermal management challenges. The results reveal that increasing the Weissenberg and Forchheimer numbers reduces the velocity profile, while thermal conductivity variation enhances heat transfer. The ternary hybrid nanofluid demonstrates superior heat transfer characteristics compared to conventional and binary hybrid nanofluids, highlighting its potential for applications in cooling systems, biomedical engineering, and industrial thermal management.

From the current analysis, we can draw the following conclusions:

• As the magnetic field effect intensifies, the interaction between the Lorentz force and the fluid’s motion results in a reduction in the velocity distribution. As the Forchheimer parameter increases, the combination of enhanced inertial resistance, nonlinear flow behavior, increased energy losses, and complex flow distributions results in a reduced velocity profile in porous media.
• As the Weissenberg number rises in a tangent hyperbolic fluid, the resulting interplay of greater elastic resistance, nonlinear flow behavior, thicker boundary layers, heightened energy dissipation, and flow stabilization leads to a reduced velocity profile.
• When the variable thermal conductivity parameter (Γ) increases, the temperature profile typically rises, reflecting the improved heat transfer capabilities of the material or fluid.
• When the non-linear thermal radiation parameter, temperature ratio parameter $\left(\theta_{w}\right)$ , heat generation ( *Q*_0_ *>* 0), Brownian motion parameter, thermophoresis parameter, magnetic field parameter, Forchheimer number ( *Fr*), and Eckert number increases, the temperature profile rises. The temperature profile diminishes, when the Prandtl number increases.
• As the volume fraction of nanoparticles $\left(\phi_{1},\phi_{2},\phi_{3}\right)$ increases in nanofluids, hybrid nanofluids, and ternary hybrid nanofluids, the temperature profile increases due to improved thermal conductivity, enhanced viscous dissipation, and better convective heat transfer.
• The concentration profile decreases as the Schmidt number, chemical reaction parameter, Brownian diffusion parameter, and suction parameter increase. Conversely, the concentration profile increases with higher values of the unsteady parameter and thermophoresis diffusion.
• In general, higher concentrations of nanoparticles in nanofluids, hybrid nanofluids, and ternary hybrid nanofluids result in increased skin friction coefficient, Nusselt number, and Sherwood number. Ternary hybrid nanofluids are often the most effective choice for enhancing heat transfer and thermal conductivity.

This study provides valuable theoretical insights but has some limitations. Simplified assumptions, such as treating the nanofluid as a single-phase fluid and neglecting higher-order effects, may not fully capture real-world complexities. The **BVP5C** numerical method, while effective, has constraints in handling highly nonlinear systems. The findings are based on a specific parameter range, and extrapolation beyond this range requires further validation. Additionally, the assumption of uniform nanoparticle dispersion overlooks possible aggregation or sedimentation effects. To enhance validity and generalizability, future research should incorporate experimental validation and more advanced modeling approaches.

## CRediT authorship contribution statement

**Asfaw Tsegaye Moltot** contributed to Writing review & editing, conceptualization, methodology, formal analysis, validation, and writing the original draft.

**Eshetu Haile** contributed to Writing the review, editing, supervision conceptualization, and resources. **Gurju Awgichew** contributed to Writing the review, editing, supervision and resources. **Hunegnaw Dessie** contributed to Writing the review, editing, supervision and resources.

Corresponding author: Asfaw Tsegaye Moltot ( asfawmat@gmail.com)

Institutional email: bdu1501759@bdu.edu.et

Affiliation: Bahir Dar University Department of Mathematics, Bahir Dar, Amhara, Ethiopia

Author name: Dr. Eshetu Haile: Email: eshetuhg@gmail.com

Research gate page: https://scholar.google.com/citations?user=GRVFCVAAAAAJ&hl=en

AD Scientific Index ID: 4494034

Affiliation: Bahir Dar University Department of Mathematics, Bahir Dar, Amhara, Ethiopia

Dr. Gurju Awgichew: Email: Fevenjerry@gmail.com

Research gate page: https://www.researchgate.net/profile/Gurju-Zergaw

Affiliation: Bahir Dar University Department of Mathematics, Bahir Dar, Amhara, Ethiopia

Dr. Hunegnaw Dessie: Email: hunegnawd@yahoo.com

Affiliation: Bahir Dar University Department of Mathematics, Bahir Dar, Amhara, Ethiopia

## Ethics and consent

Ethics and consent were not required.

## Data Availability

A list of detailed material properties used for algorithms and thermophysical properties of nanoparticle model analysis was taken from: https://github.com/asfawmat/BVP-MATLAB-Implementation BVP-MATLAB-Implementation and Thermophysical properties: https://github.com/asfawmat/BVP-MATLAB-Implementation This project contains the following data:
- Values of physical parameter and Matlab Code License: Data is available under license CC BY 4.0

## Software availability

All the presented results can be reproduced using the codes available in the GitHub links ( https://github.com/asfawmat/BVP-MATLAB-Implementation) for Matlab implementation.

Source code available from: ( https://github.com/asfawmat/BVP-MATLAB-Implementation) for Matlab implementation.

License: Data is available under license MIT License and CC-by 4.0

Archived source code available from: https://doi.org/10.5281/zenodo.14480542

Data is available under license Creative Commons Attribution 4.0 International
