# Supplementary material for: Unsteady MHD flow of tangent hyperbolic ternary hybrid nanofluid in a darcy-forchheimer porous medium over a permeable stretching sheet with variable thermal conductivity
Source: F1000Res. 2025 Mar 10;14:152. Originally published 2025 Feb 4. [Version 2] doi: 10.12688/f1000research.158629.2 (PMC11897691; doi:10.12688/f1000research.158629.2)
Supplement: Peer review history

# Reviewer report: Reviewer response for version 2

**DOI:** 10.5256/f1000research.178621.r370297

**Published:** 2025-03-22

**Authors:** Sohail Rehman

**Affiliations:** 1 Qurtuba University of Science and Information Technology, Peshawar, Pakistan

Accept in present form

Is the work clearly and accurately presented and does it cite the current literature?

No

If applicable, is the statistical analysis and its interpretation appropriate?

Partly

Are all the source data underlying the results available to ensure full reproducibility?

No

Is the study design appropriate and is the work technically sound?

Partly

Are the conclusions drawn adequately supported by the results?

Partly

Are sufficient details of methods and analysis provided to allow replication by others?

Partly

Reviewer Expertise:

Fluid dynamics, heat and mass transfer, Nanofluid

I confirm that I have read this submission and believe that I have an appropriate level of expertise to confirm that it is of an acceptable scientific standard.

# Reviewer report: Reviewer response for version 2

**DOI:** 10.5256/f1000research.178621.r370296

**Published:** 2025-03-11

**Authors:** Asra Anjum

**Affiliations:** 1 University of Technology and Applied Sciences, Salalah, Oman

Approved for acceptance.

Is the work clearly and accurately presented and does it cite the current literature?

Yes

If applicable, is the statistical analysis and its interpretation appropriate?

Yes

Are all the source data underlying the results available to ensure full reproducibility?

Yes

Is the study design appropriate and is the work technically sound?

Yes

Are the conclusions drawn adequately supported by the results?

Yes

Are sufficient details of methods and analysis provided to allow replication by others?

Yes

Reviewer Expertise:

fluid dynamics, nanofluids and hybrid nanofluids

I confirm that I have read this submission and believe that I have an appropriate level of expertise to confirm that it is of an acceptable scientific standard.

# Reviewer report: Reviewer response for version 2

**DOI:** 10.5256/f1000research.178621.r370298

**Published:** 2025-03-11

**Authors:** Prasun Choudhary, Dr. Kavita Jat

**Affiliations:** 1 Chennai Institute of Technology, Chennai, India; 2 Centre for Computational Modeling, Chennai Institute of Technology, Chennai, Tamil Nadu, India

I have reviewed the revised version of the manuscript and I appreciate the authors' efforts in incorporating the suggested comments and changes. The present version is well-structured, clear and significantly improved in terms of readability.

 As all necessary revisions have been properly addressed, I find the manuscript suitable for acceptance in its current form. I, therefore, accept and recommend it.

Is the work clearly and accurately presented and does it cite the current literature?

Yes

If applicable, is the statistical analysis and its interpretation appropriate?

Yes

Are all the source data underlying the results available to ensure full reproducibility?

Yes

Is the study design appropriate and is the work technically sound?

Yes

Are the conclusions drawn adequately supported by the results?

Yes

Are sufficient details of methods and analysis provided to allow replication by others?

Yes

Reviewer Expertise:

Fluid Flow, Heat Transfer, Mass Transfer, Distinct Geometries, Analytical Methods, Numerical Methods

We confirm that we have read this submission and believe that we have an appropriate level of expertise to confirm that it is of an acceptable scientific standard.

# Reviewer report: Reviewer response for version 1

**DOI:** 10.5256/f1000research.174254.r366057

**Published:** 2025-02-21

**Authors:** Sohail Rehman

**Affiliations:** 1 Qurtuba University of Science and Information Technology, Peshawar, Pakistan

*The main observations are listed below. The acceptance of the manuscript would depend on the revision. The author needs to provide a point by point response or provide a rebuttal.*

1. *The manuscript need a through correction of grammatical errors. The title of the manuscript need to be revised. *
2. *The authors have not explained, what motivation for selecting this fluid model is, and what are the real world applications of this fluid under the conditions studied in this paper.*
3. *Provide mathematical expression for fluid volume.*
4. * Check your boundary conditions and similarity variables. As they are dimensionally correct?*
5. *As the study considers the constitutive modeling of a virtual fluid dynamics phenomena without validating the results from any physical experimental data. While modeling such virtual fluid dynamics phenomena, the authors take many assumptions to formulate the mathematical model, which may not be real and practical. Therefore, further explanation about physical configuration of problem should be included and the results should be discussed with relation to physical parameters involved in the model. The authors should also explain the physical situation is being modeled in the manuscript.*
6. *Explain the physical significance of boundary conditions and the reasoning behind their transformations.*
7. *Detailed comparison of the current results with results from other literature or commercial (CFD) codes are preferred to show the advantages and disadvantages of the presented model?*
8. *Please enrich the introduction section with related studies *

Hashim, et al., 2023 (Ref 1)

 Rehman S, Et al., 2022 (Ref 2)

 Boujelbene M, et al., 2023 (Ref 3)

Is the work clearly and accurately presented and does it cite the current literature?

No

If applicable, is the statistical analysis and its interpretation appropriate?

Partly

Are all the source data underlying the results available to ensure full reproducibility?

No

Is the study design appropriate and is the work technically sound?

Partly

Are the conclusions drawn adequately supported by the results?

Partly

Are sufficient details of methods and analysis provided to allow replication by others?

Partly

Reviewer Expertise:

Fluid dynamics, heat and mass transfer, Nanofluid

I confirm that I have read this submission and believe that I have an appropriate level of expertise to confirm that it is of an acceptable scientific standard, however I have significant reservations, as outlined above.

## References

[1] : Coupled energy and mass transport for non-Newtonian nanofluid flow through non-parallel vertical enclosure. *Ain Shams Engineering Journal* .2023;14(8) : 10.1016/j.asej.2022.102023 10.1016/j.asej.2022.102023

[2] : Modeling a non-Newtonian nanofluid flow between intersecting planes with slip mechanism. *Continuum Mechanics and Thermodynamics* .2023;35(1) : 10.1007/s00161-022-01162-z 61-80 10.1007/s00161-022-01162-z

[3] : Optimizing thermal characteristics and entropy degradation with the role of nanofluid flow configuration through an inclined channel. *Alexandria Engineering Journal* .2023;69: 10.1016/j.aej.2023.01.026 85-107 10.1016/j.aej.2023.01.026

# Response

**Published:** 2025-02-23

**Authors:** Asfaw Tsegaye

**Affiliations:** Mathematics, Bahir Dar University, Bahir Dar, Amhara, Ethiopia

We sincerely appreciate the valuable and constructive feedback provided by the reviewer. Below, we have provided a point-by-point response, detailing the revisions made to improve the manuscript.

 1. The manuscript need a thorough correction of grammatical errors. The title of the manuscript need to be revised.

**Author’s Response: **In the revised manuscript, we have carefully reviewed and edited the entire document to correct grammatical and typographical errors.  We have revised the title to improve clarity and readability. The corrected title is:

**"Unsteady MHD Flow of Tangent Hyperbolic Ternary Hybrid Nanofluid in a Darcy-Forchheimer Porous Medium over a Permeable Stretching Sheet with Variable Thermal Conductivity."**

 2. The authors have not explained, what the motivations for selecting this fluid model is, and what are the real-world applications of this fluid under the conditions studied in this paper.

**Author’s Response:** We have added a detailed discussion in the introduction section and formulation of the method section, outlining the motivation for selecting the tangent hyperbolic ternary hybrid nanofluid model. This model is chosen due to its ability to accurately describe non-Newtonian fluid behaviors, making it suitable for engineering applications such as:

- **Cooling Systems** – Efficient heat dissipation in electronic devices.
- **Biomedical Applications** – Targeted drug delivery and hyperthermia treatments.
- **Polymer Processing** – Used in extrusion and casting processes.
- **Energy Systems** – Utilized in heat exchangers and power plants, etc.

These points are now explicitly stated in the revised introduction.

 3. Provide a mathematical expression for the fluid volume.

**Author’s Response: **The fluid volume is not considered in our study since the analysis focuses on 2D flow, boundary-layer formulation, or similarity transformation, where volume is not a key variable.

 4. Check whether the boundary conditions and similarity variables are dimensionally correct.

**Author’s Response:** We have thoroughly reviewed the boundary conditions and similarity variables to ensure dimensional correctness. The necessary corrections have been made, and a justification for each transformation is provided in the revised manuscript.

 5. As the study considers the constitutive modeling of a virtual fluid dynamics phenomena without validating the results from any physical experimental data. While modeling such virtual fluid dynamics phenomena, the authors take many assumptions to formulate the mathematical model, which may not be real and practical. Therefore, further explanation about physical configuration of problem should be included and the results should be discussed with relation to physical parameters involved in the model. The authors should also explain the physical situation is being modeled in the manuscript.

**Author’s Response: **We have expanded the discussion on the physical configuration in results and discussion section. The justification for key assumptions is also included, emphasizing:

        -The relevance of the mathematical model in practical scenarios.

        -The role of each governing parameter in defining the flow behavior.

        -The impact of approximations made to simplify the problem while maintaining accuracy, etc.

 6. Explain the physical significance of boundary conditions and the reasoning behind their transformations.

**Author’s Response: **The boundary conditions define the behavior of **unsteady MHD flow** of a tangent hyperbolic ternary hybrid nanofluid over a **permeable stretching sheet**, ensuring the model accurately represents real-world fluid dynamics. At the **sheet surface (** ***y→0*** **)**, the velocity conditions account for **fluid suction (** ***V*** ***w*** ***>*** ***0*** **)**, which enhances cooling and stabilizes the flow, and **fluid injection (** ***V*** ***w*** ***<0*** **)**, which thickens the boundary layer. The **stretching sheet condition (** ***u*** ***=*** ***λ*** ***u*** ***w*** ***(x,t)*** **)** governs flow behavior, with *λ>0* representing stretching (common in polymer processing) and *λ<0* ****representing shrinking (relevant to flow separation studies). The **temperature (** ***T*** ***=*** ***T*** ***w*** **)** and **concentration (** ***C*** ***=*** ***C*** ***w*** **​** **)** conditions define thermal and mass transfer at the surface, impacting **heat exchangers, coatings, and drug delivery applications**. Far from the sheet ( ***y→∞***), the fluid reaches **zero velocity**, **thermal equilibrium (** ***T→*** ***T*** ***∞*** **)**, and **concentration equilibrium (** ***T→*** ***T*** ***∞*** **​** **)**, ensuring a physically realistic model. These conditions are essential for accurately predicting nanofluid behavior in **industrial, biomedical, and energy systems**.

 7. Detailed comparison of the current results with results from other literature or commercial (CFD) codes are preferred to show the advantages and disadvantages of the presented model?

**Author’s Response: **We have enriched the results section by comparing our findings with existing literature, including computational studies where available. Additionally, we have included benchmark comparisons in Table 2, demonstrating the reliability of our numerical method. ****The MATLAB bvp5c solver was used with a self-developed code (GitHub link provided). The algorithm adapts the shooting method with a tolerance of 10⁻⁷, validated against Gorla & Sidawi [41], Waini et al.[40], Priyadlarshtni et al. [18], and Jamrus et al. [38].

 8. Please enrich the introduction section with related studies

- Hashim, et al., 2023 (Ref 1)
- Rehman S, Et al., 2022 (Ref 2)
- Boujelbene M, et al., 2023 (Ref 3)

**Author’s Response: **We have incorporated the suggested references in the introduction and literature review, explaining their relevance to our work. These studies provide context for our analysis and strengthen the foundation of the research.

 All revisions have been incorporated into the manuscript, enhancing clarity, technical rigor, and alignment with reviewer feedback. We appreciate the opportunity to improve our work.

# Reviewer report: Reviewer response for version 1

**DOI:** 10.5256/f1000research.174254.r366062

**Published:** 2025-02-20

**Authors:** Prasun Choudhary, Dr. Kavita Jat

**Affiliations:** 1 Chennai Institute of Technology, Chennai, India; 2 Centre for Computational Modeling, Chennai Institute of Technology, Chennai, Tamil Nadu, India

Title of the Article: Unsteady MHD flow of tangent hyperbolic ternary hybrid nanofluid in a darcy-forchheimer porous medium over a permeable stretching sheet with variable thermal conductivity

 This paper is interesting. This study examines the combined effects of viscous dissipation, Darcy-Forchheimer flow, variable thermal conductance, nonlinear thermal radiation, Joule heating, chemical reactions, heat generation/absorption, Brownian motion, and thermophoresis on the electrical conductivity of unsteady flow in tangent hyperbolic ternary hybrid nanofluids. These nanofluids consist of *Al*_2_ *O*_3_, *Cu* and *TiO*_2_ nanoparticles suspended in ethylene glycol, flowing over a stretching sheet. This study is significant as it combines theoretical analysis and practical application, providing valuable insights into the behavior of tangent hyperbolic ternary hybrid nanofluids under complex flow conditions. It has good number of applications in engineering and technology.

 It may be accepted subject to the following major modifications/corrections:

 1. Title is mostly well-structured and conveys the research topic clearly. However, "Darcy-Forchheimer" should be capitalized because it refers to a specific model.

 2. How do the thermal conductivity and viscosity of ethylene glycol-based nanofluids vary with the dispersion of aluminum oxide (Al₂O₃), copper (Cu) and titanium oxide (TiO₂) nanoparticles?

 3. What are the nonlinearities in the governing equations and how do they influence the similarity transformations?

 4. Describe advantages and limits of ternary hybrid nanofluids in high-performance cooling systems.

 5. Mention the potential biomedical implications of ternary hybrid nanofluids in drug delivery applications?

 6. Literature review is also to be enriched with recent articles.Add the below articles in your introduction:

 MHD thermal and solutal stratified stagnation flow of tangent hyperbolic fluid induced by stretching cylinder with dual convection;

 Impacts of unsteady MHD hybrid nanofluid over a non-linear stretchable porous sheet with thermal radiation and gyrotatic microorganisms;

 Tangent hyperbolic fluid flow under condition of divergent channel in the presence of porous medium with suction/blowing and heat source: Emergence of the boundary layer;

 Significances of melting heat transfer and bio-convection phenomena in nanofluid flow over a three different geometries;

 Computational study of the magnetohydrodynamic copper–water nanofluid flow through concentric cylinders immersed in a porous medium;

 Bio-convection of a radiating and reacting nanofluid flow past a nonlinear stretchable permeable sheet in a porous medium.

 7. Authors use unsteady magnetic field B _0_=B/(1−ct) ^1/2^. Can this field induce an electric field according to Maxwell’s equations and what are its implications? Justify and mention it in revised version.

 8. Explanation related to boundary condition (9) needs more clarity.

 9. Velocity plots with We and *ϕ*_1 _show very less variations. Recheck and justify the reasons for these less variations.

 10. What are the benchmarks of your study?

 11. How are convergence and stability ensured in the computational analysis?

 12. The flow is in a permeable stretching sheet, what will be the role of the magnetic field?

 13. Add novel results in the conclusion section.

 I have cited few papers because they are closely aligned with the author's manuscript. The cited papers provide a detailed explanation of the tangent hyperbolic fluid model, which is only partially covered in the author's paper. Additionally, the topics of MHD and porous media are extensively discussed in the cited works which offers valuable insights that complement the author's discussion.

Is the work clearly and accurately presented and does it cite the current literature?

Yes

If applicable, is the statistical analysis and its interpretation appropriate?

Yes

Are all the source data underlying the results available to ensure full reproducibility?

Yes

Is the study design appropriate and is the work technically sound?

Yes

Are the conclusions drawn adequately supported by the results?

Yes

Are sufficient details of methods and analysis provided to allow replication by others?

Yes

Reviewer Expertise:

Fluid Flow, Heat Transfer, Mass Transfer, Distinct Geometries, Analytical Methods, Numerical Methods

We confirm that we have read this submission and believe that we have an appropriate level of expertise to confirm that it is of an acceptable scientific standard, however we have significant reservations, as outlined above.

## References

[1] : MHD Thermal and Solutal Stratified Stagnation Flow of Tangent Hyperbolic Fluid Induced by Stretching Cylinder with Dual Convection. *Mathematics* .2023;11(9) : 10.3390/math11092182 10.3390/math11092182

[2] : Impacts of unsteady MHD hybrid nanofluid over a non-linear stretchable porous sheet with thermal radiation and gyrotatic microorganisms. *International Journal of Thermofluids* .2024;23: 10.1016/j.ijft.2024.100788 10.1016/j.ijft.2024.100788

[3] : Tangent Hyperbolic Fluid Flow under Condition of Divergent Channel in the Presence of Porous Medium with Suction/Blowing and Heat Source: Emergence of the Boundary Layer. *International Journal of Mathematics and Mathematical Sciences* .2023;2023(1) : 10.1155/2023/6282130 10.1155/2023/6282130

[4] : Significances of melting heat transfer and bioconvection phenomena in nanofluid flow over a three different geometries. *International Journal of Thermofluids* .2024;24: 10.1016/j.ijft.2024.100855 10.1016/j.ijft.2024.100855

[5] : Computational study of the magnetohydrodynamic copper–water nanofluid flow through concentric cylinders immersed in a porous medium. *Journal of Thermal Analysis and Calorimetry* .2025; 10.1007/s10973-024-13900-3 10.1007/s10973-024-13900-3

[6] : Novel features of radiating hybrid nanofluid flow past a nonlinear stretchable porous sheet with different nanoparticles shape. *Numerical Heat Transfer, Part B: Fundamentals* .2024; 10.1080/10407790.2024.2386584 1-25 10.1080/10407790.2024.2386584

# Response

**Published:** 2025-02-23

**Authors:** Asfaw Tsegaye

**Affiliations:** Mathematics, Bahir Dar University, Bahir Dar, Amhara, Ethiopia

**Authors’ Response to Reviewers’ Comments**

 We sincerely appreciate the reviewers for their insightful and constructive comments, which have significantly improved the quality of our manuscript. Below, we provide our responses to each of the reviewers’ concerns and outline the corresponding revisions made in the manuscript.

 1. The title is mostly well-structured and conveys the research topic clearly. However, "Darcy-Forchheimer" should be capitalized because it refers to a specific model.

**Authors' Response:** We have corrected the capitalization in the title as suggested. The revised title now reads:

**"Unsteady MHD Flow of Tangent Hyperbolic Ternary Hybrid Nanofluid in a Darcy-Forchheimer Porous Medium over a Permeable Stretching Sheet with Variable Thermal Conductivity."**

 2. How do the thermal conductivity and viscosity of ethylene glycol-based nanofluids vary with the dispersion of aluminum oxide (Al₂O₃), copper (Cu), and titanium oxide (TiO₂) nanoparticles?

**Authors' Response: **The thermal conductivity and viscosity of ethylene glycol-based nanofluids are significantly influenced by the dispersion of nanoparticles such as Al₂O₃, Cu, and TiO₂. In particular, ternary hybrid nanofluids—those containing a combination of three different nanoparticles—exhibit unique thermophysical behaviors.

 The dispersion of Al₂O₃, Cu, and TiO₂ nanoparticles in ethylene glycol-based ternary hybrid nanofluids leads to enhanced thermal conductivity and increased viscosity. The extent of these changes depends on nanoparticle concentration and operating temperature.  Viscosity tends to decrease with rising temperatures. Notably, while thermal conductivity improves with elevated temperatures, viscosity decreases, indicating better flow characteristics at higher temperatures. Balancing these factors is essential for optimizing the performance of such nanofluids in practical applications. While viscosity changes are not explicitly addressed, it is generally understood that nanoparticle addition can increase fluid viscosity, potentially impacting flow behavior.

 3. What are the nonlinearities in the governing equations and how do they influence the similarity transformations?

**Authors' Response: **The governing equations exhibit nonlinearities due to factors such as the tangent hyperbolic fluid model, where the shear stress follows a hyperbolic tangent function, introducing nonlinearity in the momentum equation. Nonlinear thermal radiation further complicates the energy equation with temperature-dependent radiative heat flux, while variable thermal conductivity leads to additional nonlinear terms. Viscous dissipation and Joule heating contribute quadratic velocity and temperature-dependent effects, and the Darcy–Forchheimer drag forces in the porous medium introduce nonlinear velocity terms. These nonlinearities make the transformation of partial differential equations (PDEs) into ordinary differential equations (ODEs) via similarity transformations more complex. The resulting nonlinear ODEs require numerical solutions, such as the BVP5C algorithm in MATLAB, where stability, convergence, and accuracy depend on careful parameter tuning for computational efficiency.

 4. Describe advantages and limits of ternary hybrid nanofluids in high-performance cooling systems.

**Authors' Response: **Ternary hybrid nanofluids enhance high-performance cooling systems by combining nanoparticles like Al₂O₃, Cu, and TiO₂, leading to superior thermal conductivity, improved stability, and controlled viscosity for efficient heat transfer. Their adaptability makes them ideal for applications in electronics cooling, automotive radiators, and industrial heat exchangers. By optimizing nanoparticle composition, properties like specific heat and viscosity can be tailored to meet cooling demands. However, these benefits come with challenges such as higher synthesis costs, increased viscosity that may raise pumping power requirements, and potential issues with particle agglomeration, sedimentation, and system compatibility. Additionally, their performance varies with temperature, shear rate, and composition, necessitating extensive testing and optimization. Despite these limitations, ternary hybrid nanofluids remain a promising solution for enhancing cooling efficiency, provided their formulation and application challenges are carefully managed.

 5. Mention the potential biomedical implications of ternary hybrid nanofluids in drug delivery applications.

**Authors' Response: **Ternary hybrid nanofluids hold significant potential in drug delivery applications due to their enhanced thermal and rheological properties. Their **high thermal conductivity** allows for precise temperature control, making them suitable for **targeted hyperthermia treatments** in cancer therapy. The **improved stability and dispersion** of multiple nanoparticles ensure better drug encapsulation and controlled release, enhancing bioavailability. Their **adjustable viscosity** enables efficient flow through micro capillaries, improving circulation and targeted delivery to specific tissues. Additionally, the presence of multiple nanoparticles (e.g., Al₂O₃, Cu, TiO₂) can enhance **cellular uptake and bioactivity**, increasing drug efficacy. However, challenges such as **biocompatibility, toxicity, and long-term stability** must be addressed to ensure their safe and effective use in biomedical applications.

 6. The literature review should be enriched with recent articles.

**Authors' Response: **We have incorporated the suggested references into the introduction and literature review sections, specifically discussing how they relate to our study. The following references have been added:

     - MHD thermal and solutal stratified stagnation flow of tangent hyperbolic fluid induced by stretching cylinder with dual convection;

      - Impacts of unsteady MHD hybrid nanofluid over a non-linear stretchable porous sheet with thermal radiation and gyrotatic microorganisms;  

       -Tangent hyperbolic fluid flow under condition of divergent channel in the presence of porous medium with suction/blowing and heat source: Emergence of the boundary layer;  

       - Significances of melting heat transfer and bio-convection phenomena in nanofluid flow over a three different geometries;  

       - Computational study of the magnetohydrodynamic copper–water nanofluid flow through concentric cylinders immersed in a porous medium; 

        - Bio-convection of a radiating and reacting nanofluid flow past a nonlinear stretchable permeable sheet in a porous medium.

 7. Authors use unsteady magnetic field B0=B/(1−ct)1/2. Can this field induce an electric field according to Maxwell’s equations and what are its implications? Justify and mention it in revised version.

**Authors' Response: **We have addressed this concern in the mathematical formulation section by discussing Maxwell’s equations in the context of our study.  This justification has been added to the revised manuscript.

 8. Explanation related to boundary condition (9) needs more clarity.

**Authors' Response: **The boundary conditions define the behavior of **unsteady MHD flow** of a tangent hyperbolic ternary hybrid nanofluid over a **permeable stretching sheet**, ensuring the model accurately represents real-world fluid dynamics. At the **sheet surface (** ***y→0*** **)**, the velocity conditions account for **fluid suction (** ***V*** ***w*** ***>*** ***0*** **)**, which enhances cooling and stabilizes the flow, and **fluid injection (** ***V*** ***w*** ***<0*** **)**, which thickens the boundary layer. The **stretching sheet condition (** ***u*** ***=*** ***λ*** ***u*** ***w*** ***(x,t)*** **)** governs flow behavior, with *λ>0* representing stretching (common in polymer processing) and *λ<0* ****representing shrinking (relevant to flow separation studies). The **temperature (** ***T*** ***=*** ***T*** ***w*** **)** and **concentration (** ***C*** ***=*** ***C*** ***w*** **​** **)** conditions define thermal and mass transfer at the surface, impacting **heat exchangers, coatings, and drug delivery applications**. Far from the sheet ( ***y→∞***), the fluid reaches **zero velocity**, **thermal equilibrium (** ***T→*** ***T*** ***∞*** **)**, and **concentration equilibrium (** ***T→*** ***T*** ***∞*** **​** **)**, ensuring a physically realistic model. These conditions are essential for accurately predicting nanofluid behavior in **industrial, biomedical, and energy systems**.

 9. Velocity plots with We and ϕ1 show very less variations. Recheck and justify the reasons for these less variations.

**Authors' Response: **The velocity plots with respect to the Weissenberg number ( *We*) and nanoparticle volume fraction ( *ϕ* *1*​) exhibit minimal variation. This limited influence can be attributed to the dominance of viscous effects over elasticity in the given flow regime, the relatively small effect of nanoparticle volume fraction on velocity, and the constraints imposed by the boundary conditions. If *ϕ* *1*​ ​ is varied within a narrow range, the increase in viscosity may not be substantial enough to noticeably affect the velocity profile.

 10. What are the benchmarks of your study?

**Authors' Response: **The numerical results have been validated against previously published studies, as shown in Table 2. Our findings show strong agreement with benchmark solutions, confirming the accuracy of our computational approach.

 11. How are convergence and stability ensured in the computational analysis?

**Authors' Response: **We have explicitly discussed the convergence criteria of the BVP5C algorithm and verified that the residual errors remain within acceptable limits. The computational analysis employs BVP5C, a MATLAB solver for boundary value problems, ensuring convergence and stability through adaptive mesh refinement, in our study, error control (tolerance *10* *-7*), step size regulation, and a well-posed problem formulation. A grid independence test verifies numerical stability, ensuring accurate and reliable results while minimizing numerical artifacts.

 12. The flow is in a permeable stretching sheet, what will be the role of the magnetic field?

**Authors' Response: **We have elaborated on the role of the magnetic field in modifying velocity and temperature profiles. The Lorentz force acts as a resistive force, reducing velocity and increasing temperature due to Joule heating.

 13. Add novel results in the conclusion section.

**Authors' Response: **We have updated the conclusion to explicitly highlight the novel contributions of this study, including:

- The interplay between ternary hybrid nanoparticles and non-Newtonian fluid properties.
- The effect of variable thermal conductivity on heat transfer performance.
- The impact of nonlinear radiation on temperature enhancement, etc.

These additions strengthen the conclusion and emphasize the novelty of our findings.

 All revisions have been incorporated into the manuscript, enhancing clarity, technical rigor, and alignment with reviewer feedback. We appreciate the opportunity to improve our work.

 Sincerely,

 Asfaw Tsegaye Moltot[Corresponding Author]

# Reviewer report: Reviewer response for version 1

**DOI:** 10.5256/f1000research.174254.r366061

**Published:** 2025-02-18

**Authors:** Asra Anjum

**Affiliations:** 1 University of Technology and Applied Sciences, Salalah, Oman

**Title: Unsteady MHD flow of tangent hyperbolic ternary hybrid nanofluid in a darcy-forchheimer porous medium over a permeable stretching sheet with variable thermal c** **onductivity**

 The paper has made a promising contribution and fresh results. However, the article needs modifications, and **I'd like to ask the authors to revise it due to the following comments, which can be accepted.**

 Review comments;

 1. Please briefly state more about salient findings in the abstract.

 2. Nomenclature should be mentioned along with SI units. Similarly, subscripts and superscripts can be explained under their subtitles.

 3. It is recommended the authors try to explain the novelty of the paper as clearly as possible and explain the research gap they are trying to fill.

 4. The introduction section requires a comprehensive survey of recent literature. The authors should conduct a detailed literature review, clearly outlining the contributions of each relevant study. Additionally, the introduction should explain how the findings from previous works support and inform the current research, making it clear how these studies contribute to the development of the present study's objectives and approach. The following articles will help to enhance the quality of the introduction section:

 5. The author needs to mention briefly the application of the present work in the real world.

 6. Why are such boundary conditions used? What is the physical meaning?

 7. A correlation must be suggested based on the computed results (You can use statistical tools or methods for correlation calculation).

 8. What software is used for the simulations? Was the code implemented by the authors or a function already existing in the software was used? If the code for the numerical method was taken from another publication or is part of the software used, please cite the resource.

 9. Write the advantages of the used numerical methods in detail.

 10. The author should give a deep analysis of the results shown in the figures.

 11. Revise the article's conclusion to make it more impactful and ensure it effectively reinforces the main points and leaves a lasting impression on the reader.

 12. Does the conclusion sufficiently address the study's limitations and explain how these constraints affect the findings' overall validity and generalizability?

**13. **What are the key physical parameters analyzed in the study, and how do they influence the Nusselt number, Sherwood number, skin friction?

 14. What potential applications are suggested for the mathematical framework developed in the study, particularly concerning nanofluid behavior?

 15. What unique aspects of the study contribute to its novelty in investigating nanofluid convection and heat and mass transfer phenomena?

**16** **. **You should describe the abbreviations while using for first time.

**17** **. **You should add major findings in abstract.

**18** **. **Write the references in correct way. There are some recent references which is important for the manuscript:

 19 **.** Explain the physical significance of boundary conditions.

**20. **You should add a figure for validation of your code.

 21. You should rewrite the first paragraph of Conclusions section.

  22 **. **There are a lot of errors in typing and word spacing. It must be corrected in the revised manuscript.

**23** **. **What is the impact of this article? What are the future directions?

Is the work clearly and accurately presented and does it cite the current literature?

Yes

If applicable, is the statistical analysis and its interpretation appropriate?

Yes

Are all the source data underlying the results available to ensure full reproducibility?

Yes

Is the study design appropriate and is the work technically sound?

Yes

Are the conclusions drawn adequately supported by the results?

Yes

Are sufficient details of methods and analysis provided to allow replication by others?

Yes

Reviewer Expertise:

fluid dynamics, nanofluids and hybrid nanofluids

I confirm that I have read this submission and believe that I have an appropriate level of expertise to confirm that it is of an acceptable scientific standard, however I have significant reservations, as outlined above.

# Response

**Published:** 2025-02-23

**Authors:** Asfaw Tsegaye

**Affiliations:** Mathematics, Bahir Dar University, Bahir Dar, Amhara, Ethiopia

​​​​ **Author’s Response to Reviewers’ Comments**

 We sincerely appreciate the reviewers for their insightful and constructive comments, which have significantly improved the quality of our manuscript. Below, we provide our responses to each of the reviewer’s concerns and outline the corresponding revisions made in the manuscript.

 1. Please briefly state more about salient findings in the abstract.

**Authors' reply: **The abstract has been revised to explicitly highlight key findings, including:

 - The velocity profile decreases with increasing Weissenberg and Forchheimer numbers.

 - Ternary hybrid nanofluids exhibit superior heat transfer enhancement compared to mono/hybrid nanofluids.

  - Variable thermal conductivity (Γ) and nanoparticle volume fractions significantly improve temperature distribution and skin friction.

 - Applications in cooling systems, biomedical drug delivery, and polymer processing are emphasized.

 2. Nomenclature should be mentioned along with SI units. Similarly, subscripts and superscripts can be explained under their subtitles.

**Authors' reply: **The nomenclature section has been updated to include SI units for all parameters. Subscripts and superscripts are now clearly defined under their respective sections.

 3. It is recommended the authors try to explain the novelty of the paper as clearly as possible and explain the research gap they are trying to fill.

**Authors' reply: **A new paragraph was added to the introduction (last paragraph) to clarify the novelty:

 "To the authors' knowledge, no prior study combines viscous dissipation, Darcy-Forchheimer flow, variable thermal conductivity, and ternary hybrid nanofluids (Al₂O₃–Cu–TiO₂/EG) under unsteady MHD conditions. This work addresses the gap by analyzing synergistic effects of 12+ parameters, offering insights into optimizing thermal systems."

 4. The introduction section requires a comprehensive survey of recent literature. The authors should conduct a detailed literature review, clearly outlining the contributions of each relevant study. Additionally, the introduction should explain how the findings from previous works support and inform the current research, making it clear how these studies contribute to the development of the present study's objectives and approach. The following articles will help to enhance the quality of the introduction section:

**Authors' reply: **We have not yet received your suggested references. However, we have added recent studies (2023–2024) on ternary hybrid nanofluids and tangent hyperbolic fluids. Additionally, using your Google Scholar profile, we have incorporated some of your references into the introduction and literature review sections, highlighting their relevance to our study. The following references have been added:

 - Computation Of Convective Magnetohydrodynamic Buongiorno Nanofluid Transport From An Inclined Plane With Ion Slip And Hall Current Effects

- Influence of Darcy-Forchheimer hybrid nanofluid flow with magnetohydrodynamic and viscous dissipation effects past a permeable stretching sheet: a numerical contribution

 5. The author needs to mention briefly the application of the present work in the real world.

**Authors' reply: **The study titled "Unsteady MHD flow of tangent hyperbolic ternary hybrid nanofluid in a Darcy-Forchheimer porous medium over a permeable stretching sheet with variable thermal conductivity" investigates the unsteady magnetohydrodynamic (MHD) flow, heat, and mass transfer of ternary hybrid nanofluids over a permeable stretching sheet. The study considers three types of nanoparticles—aluminum oxide (Al₂O₃), copper (Cu), and titanium oxide (TiO₂)—dispersed in a base fluid of ethylene glycol (C₂H₆O₂). This ternary hybrid nanofluid (Al₂O₃–Cu–TiO₂/C₂H₆O₂) has potential applications in cooling systems, biomedical uses for targeted drug delivery and hyperthermia treatments, heat exchangers, and polymer processing techniques like extrusion and casting.

 6. Why are such boundary conditions used? What is the physical meaning?

**Authors' reply: **In the study titled "Unsteady MHD flow of tangent hyperbolic ternary hybrid nanofluid in a Darcy-Forchheimer porous medium over a permeable stretching sheet with variable thermal conductivity," the authors employ specific boundary conditions to accurately model the physical phenomena under investigation.

 These conditions are essential for representing real-world scenarios and ensuring the validity of the mathematical model. These boundary conditions are crucial for accurately modeling the unsteady MHD flow of ternary hybrid nanofluids over a permeable stretching sheet with variable thermal conductivity. They ensure that the mathematical model reflects the physical realities of the system, including the interaction between the fluid and the surface, the development of boundary layers, and the transition to ambient conditions at a distance from the surface.

 7. A correlation must be suggested based on the computed results (You can use statistical tools or methods for correlation calculation).

**Authors' reply: **In our study **, **the combined effects of several factors on the unsteady MHD flow of ternary hybrid nanofluids over a permeable stretching sheet. Specifically, the research examines the influence of viscous dissipation, Darcy-Forchheimer flow, variable thermal conductivity, nonlinear thermal radiation, Joule heating, chemical reactions, heat generation/absorption, Brownian motion, and thermophoresis on the velocity profile, temperature distribution, concentration profile, skin friction, Nusselt number, and Sherwood number. In this study, correlation were not suggested. ****We considering the further direction.

 8. What software is used for the simulations? Was the code implemented by the authors or a function already existing in the software was used? If the code for the numerical method was taken from another publication or is part of the software used, please cite the resource.

**Authors' reply:** The MATLAB bvp5c solver was used with a self-developed code (GitHub link provided). The algorithm adapts the shooting method with a tolerance of 10⁻⁷, validated against Gorla & Sidawi [41], Waini et al.[40], Priyadlarshtni et al. [18], and Jamrus et al. [38].

 9. Write the advantages of the used numerical methods in detail.

**Authors' reply:** BVP5C is a powerful tool for solving boundary value problems in various scientific and engineering applications. Its versatility, efficiency, and user-friendly nature make it an essential component in numerical analysis.  The **BVP5C** solver provides high accuracy for stiff boundary value problems, leveraging adaptive mesh refinement and efficiently handling nonlinearities. Compared to finite difference methods, it offers faster convergence with minimal dependence on grid size.

 10. The author should give a deep analysis of the results shown in the figures.

**Authors' reply:** The detailed analysis of the figures in this study provides a comprehensive understanding of how various parameters influence the flow, heat, and mass transfer characteristics of ternary hybrid nanofluids. These insights are valuable for optimizing the performance of nanofluids in applications such as cooling systems, heat exchangers, and polymer processing.

 11. Revise the article's conclusion to make it more impactful and ensure it effectively reinforces the main points and leaves a lasting impression on the reader.

**Authors' reply:** It is modified in the conclusion section in the revised manuscript.

 12. Does the conclusion sufficiently address the study's limitations and explain how these constraints affect the findings' overall validity and generalizability?

**Authors' reply:** This study provides valuable theoretical insights but has some limitations. Simplified assumptions, such as treating the nanofluid as a single-phase fluid and neglecting higher-order effects, may not fully capture real-world complexities. The **BVP5C** numerical method, while effective, has constraints in handling highly nonlinear systems. The findings are based on a specific parameter range, and extrapolation beyond this range requires further validation. Additionally, the assumption of uniform nanoparticle dispersion overlooks possible aggregation or sedimentation effects. To enhance validity and generalizability, future research should incorporate experimental validation and more advanced modeling approaches.

 13. What are the key physical parameters analyzed in the study, and how do they influence the Nusselt number, Sherwood number, skin friction?

**Authors' reply:** The study demonstrates that the key physical parameters—especially the nanoparticle concentration, Weissenberg number, Forchheimer number, and variable thermal conductivity—have significant effects on the heat and mass transfer characteristics. These parameters enhance the Nusselt number, Sherwood number, and skin friction, contributing to the improved performance of ternary hybrid nanofluids in heat exchange and mass transfer applications. We have addressed this in the results and conclusion sections. Please refer to page 26.

 14. What potential applications are suggested for the mathematical framework developed in the study, particularly concerning nanofluid behavior?

**Authors' reply:** The mathematical framework developed for unsteady MHD flow of tangent hyperbolic ternary hybrid nanofluids over a permeable stretching sheet with variable thermal conductivity provides valuable insights into nanofluid behavior under various conditions. It has potential applications in **cooling systems**, optimizing thermal management in electronics, power plants, and automotive systems. In **biomedical applications**, it aids in understanding nanofluid flow for targeted drug delivery and hyperthermia treatments. The framework also supports the **design of heat exchangers**, enhancing industrial heat transfer efficiency. Additionally, it improves **polymer processing** by predicting material flow and temperature control in extrusion and casting. In **energy systems**, it contributes to advanced solar thermal collectors and photovoltaic/thermal (PV/T) systems, promoting sustainable energy solutions. By leveraging this model, researchers can enhance the efficiency of various thermal and industrial applications.

 15. What unique aspects of the study contribute to its novelty in investigating nanofluid convection and heat and mass transfer phenomena?

**Authors' reply:** This study introduces several novel aspects in investigating unsteady MHD flow of tangent hyperbolic ternary hybrid nanofluids over a permeable stretching sheet with variable thermal conductivity. It uniquely integrates multiple effects, including viscous dissipation, Darcy-Forchheimer flow, nonlinear thermal radiation, Joule heating, and thermophoresis, providing a comprehensive understanding of nanofluid behavior. Unlike previous studies on binary hybrid nanofluids, this research explores ternary hybrid nanofluids (Al₂O₃–Cu–TiO₂ in ethylene glycol), offering superior thermal properties. The study’s focus on permeable stretching sheets enhances its relevance for real-world applications such as cooling systems and polymer processing. By incorporating **variable thermal conductivity**, it improves heat transfer accuracy, while an extensive **parametric analysis** further clarifies the effects of key influencing factors. These contributions make the study a significant advancement in nanofluid convection and heat transfer research.

 16. You should describe the abbreviations while using for first time.

**Authors' reply:** It is modified in the revised manuscript.

 17. You should add major findings in abstract.

**Authors' reply:** We incorporate the major finding in the abstract.

 18. Write the references in correct way. There are some recent references which is important for the manuscript:

**Authors' reply:** Modified

 19. Explain the physical significance of boundary conditions.

**Authors reply:** The boundary conditions define the behavior of **unsteady MHD flow** of a tangent hyperbolic ternary hybrid nanofluid over a **permeable stretching sheet**, ensuring the model accurately represents real-world fluid dynamics. At the **sheet surface (** ***y→0*** **)**, the velocity conditions account for **fluid suction (** ***V*** ***w*** ***>*** ***0*** **)**, which enhances cooling and stabilizes the flow, and **fluid injection (** ***V*** ***w*** ***<*** ***0***  **)**, which thickens the boundary layer. The **stretching sheet condition (** ***u*** ***=*** ***λ*** ***u*** ***w*** ***(x,t)***  **)** governs flow behavior, with *λ>0*  representing stretching (common in polymer processing) and *λ<0*  ****representing shrinking (relevant to flow separation studies). The **temperature (** ***T*** ***=*** ***T*** ***w***  **)** and **concentration (** ***C*** ***=*** ***C*** ***w***  **​** **)** conditions define thermal and mass transfer at the surface, impacting **heat exchangers, coatings, and drug delivery applications**. Far from the sheet ( ***y→*** ***∞*** ), the fluid reaches **zero velocity**, **thermal equilibrium (** ***T→*** ***T*** ***∞***  **)**, and **concentration equilibrium (** ***T→*** ***T*** ***∞***  **​** **)**, ensuring a physically realistic model. These conditions are essential for accurately predicting nanofluid behavior in **industrial, biomedical, and energy systems**.

 20. You should add a figure for validation of your code.

**Authors' reply:** This study provides sufficient validation through comprehensive comparisons with existing literature and numerical benchmarks

 .

 21. You should rewrite the first paragraph of conclusions section.

**Authors' reply:** Modified in the revised manuscript.

 22. There are a lot of errors in typing and word spacing. It must be corrected in the revised manuscript.

**Authors' reply:** Corrected

 23. What is the impact of this article? What are the future directions?

**Authors' reply:** This study provides a detailed analysis of unsteady MHD flow in ternary hybrid nanofluids ( **Al₂O₃–Cu–TiO₂ in ethylene glycol**) over a permeable stretching sheet with variable thermal conductivity. The findings have significant applications in **cooling systems**, improving heat transfer efficiency, **biomedical applications**, aiding in drug delivery and hyperthermia treatments, and **polymer processing**, optimizing thermal conditions in extrusion and casting. Future research should focus on **experimental validation** to confirm numerical results, **optimization studies** to refine nanoparticle concentrations for maximum efficiency, and **long-term stability analysis** to ensure reliability in industrial applications. These advancements will enhance the practical utility of ternary hybrid nanofluids across various engineering and technological fields.

 All revisions have been incorporated into the manuscript, enhancing clarity, technical rigor, and alignment with reviewer feedback. We appreciate the opportunity to improve our work.

 Sincerely,

 Asfaw Tsegaye Moltot[Corresponding Author]

 Email: asfawmat@gmail.com
